# Polyvalent Glycomimetic-Gold Nanoparticles Revealing Critical Roles of Glycan Display on Multivalent Lectin–Glycan Interaction Biophysics and Antiviral Properties

**DOI:** 10.1021/jacsau.4c00610

**Published:** 2024-08-15

**Authors:** Xinyu Ning, Darshita Budhadev, Sara Pollastri, Inga Nehlmeier, Amy Kempf, Iain Manfield, W. Bruce Turnbull, Stefan Pöhlmann, Anna Bernardi, Xin Li, Yuan Guo, Dejian Zhou

**Affiliations:** † School of Chemistry and Astbury Centre for Structural Molecular Biology, University of Leeds, Leeds LS2 9JT, United Kingdom; ‡ Dipartimento di Chimica, Universita′ Degli Studi di Milano, via Golgi 19, Milano 20133, Italy; § Infection Biology Unit, German Primate Center—Leibniz Institute for Primate Research, 37077 Göttingen, Germany; ∥ Faculty of Biology and Psychology, University of Göttingen, 37073 Göttingen, Germany; ⊥ School of Food Science & Nutrition and Astbury Centre for Structural Molecular Biology, University of Leeds, Leeds LS2 9JT, United Kingdom; # Building One, Granta Centre, G ranta Park, Sphere Fluidics Ltd, Great Abington, Cambridge CB21 6AL, United Kingdom; ∇ School of Molecular and Cellular Biology and Astbury Centre for Structural Molecular Biology, University of Leeds, Leeds LS2 9JT, United Kingdom

**Keywords:** gold nanoparticle, glycoconjugate, multivalent lectin–glycan interaction, glycomimetic, fluorescence quenching, binding thermodynamics, virus inhibition

## Abstract

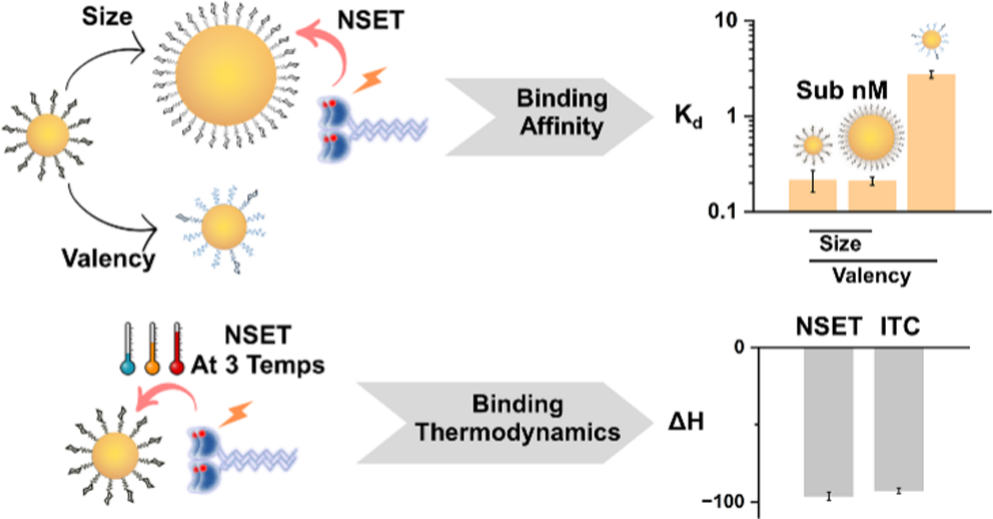

Multivalent lectin–glycan interactions (MLGIs) are widespread and vital for biology, making them attractive therapeutic targets. Unfortunately, the structural and biophysical mechanisms of several key MLGIs remain poorly understood, limiting our ability to design spatially matched glycoconjugates as potential therapeutics against specific MLGIs. We have recently demonstrated that natural oligomannose-coated nanoparticles are powerful probes for MLGIs. They can provide not only quantitative affinity and binding thermodynamic data but also key structural information (*e.g*, binding site orientation and mode) useful for designing glycoconjugate therapeutics against specific MLGIs. Despite success, how designing parameters (*e.g*., glycan type, density, and scaffold size) control their MLGI biophysical and antiviral properties remains to be elucidated. A synthetic pseudodimannose (psDiMan) ligand has been shown to selectively bind to a dendritic cell surface tetrameric lectin, DC-SIGN, over some other multimeric lectins sharing monovalent mannose specificity but having distinct cellular functions. Herein, we display psDiMan polyvalently onto gold nanoparticles (GNPs) of varying sizes (*e.g*., ∼5 and ∼13 nm, denoted as G5- and G13 psDiMan hereafter) to probe how the scaffold size and glycan display control their MLGI properties with DC-SIGN and the closely related lectin DC-SIGNR. We show that G5/13 psDiMan binds strongly to DC-SIGN, with sub-nM *K*_d_s, with affinity being enhanced with increasing scaffold size, whereas they show apparently no or only weak binding to DC-SIGNR. Interestingly, there is a minimal, GNP-size-dependent, glycan density threshold for forming strong binding with DC-SIGN. By combining temperature-dependent affinity and Van’t Hoff analyses, we have developed a new GNP fluorescence quenching assay for MLGI thermodynamics, revealing that DC-SIGN-G*x*-psDiMan binding is enthalpy-driven, with a standard binding Δ*H*^0^ of ∼ −95 kJ mol^–1^, which is ∼4-fold that of the monovalent binding and is comparable to that measured by isothermal titration calorimetry. We further reveal that the enhanced DC-SIGN affinity with G*x*-psDiMan with increasing GNP scaffold size is due to reduced binding entropy penalty and not due to enhanced favorable binding enthalpy. We further show that DC-SIGN binds tetravalently to a single G*x*-psDiMan, irrespective of the GNP size, whereas DC-SIGNR binding is dependent on GNP size, with no apparent binding with G5, and weak cross-linking with G13. Finally, we show that G*x*-psDiMans potently inhibit DC-SIGN-dependent augmentation of cellular entry of Ebola pseudoviruses with sub-nM EC_50_ values, whereas they exhibit no significant (for G5) or weak (for G13) inhibition against DC-SIGNR-augmented viral entry, consistent to their MLGI properties with DC-SIGNR in solution. These results have established G*x*-psDiMan as a versatile new tool for probing MLGI affinity, selectivity, and thermodynamics, as well as GNP–glycan antiviral properties.

## 1 Introduction

Multivalent lectin–glycan interactions (MLGIs) are widespread and vital for many important biological events, such as infection, cell–cell communication, and the regulation of immune response.^1−6^ For example, pathogens often employ specific glycan patterns to target host cell lectin receptors (or vice versa) to initiate contact and infection, while immune cells often employ lectins to recognize specific pathogen-associated glycan patterns to differentiate pathogens and to instruct immune responses.^4−10^ Therefore, it is unsurprising that constructing glycan structures to target specific MLGIs has been a very active and attractive therapeutic approach against a wide range of viral infections, cancer, and other immune dysregulation diseases.^1−5,11−18^ Strategies employed often include the design of monovalent glycans against specific structures of individual carbohydrate recognition domains (CRDs) and displaying glycans multi/polyvalently onto various nanoscale scaffolds.^1,2,11−26^ This is mainly because most monovalent glycan–CRD interactions are too weak to produce high enough therapeutical effects. Displaying multiple glycans on a suitable scaffold to create a perfect spatial and orientation match to the target lectin’s multiple CRDs will greatly enhance not only their binding affinity but also specificity.^11,25^ The latter is of great importance for potential applications *in vivo* due to the overlapping glycan specificity, at the monovalent levels, of various multimeric lectins.^4^

A wide variety of nanostructures, *e.g*., polymers, dendrimers, liposomes, polymersomes, proteins, and inorganic nanoparticles, have been employed as scaffolds to construct multi/polyvalent glycoconjugates to enhance their MLGI affinity and specificity.^1−3,11−17,19−28^ The biophysical parameters of binding to target lectins are mainly evaluated by conventional biophysical techniques, such as surface plasmon resonance (SPR),^29^ and isothermal titration calorimetry (ITC).^30,31^ While these traditional biophysical methods are powerful in obtaining quantitative binding affinity, kinetic, and thermodynamic data, they cannot provide key structural information, *e.g*., binding site organization, binding mode, interbinding site distances, etc., which are of critical importance for designing spatial matched glycoconjugates against a particular MLGI for therapeutic interventions. In addition, each of these techniques also suffers from its own limitations. For example, while ITC can provide accurate measure of binding enthalpy changes (Δ*H*s), it cannot directly provide accurate binding affinities for very strong interactions (low- to sub-nM *K*_d_s).^32,33^ Whereas, SPR measures binding interactions happening on surfaces, which is a very different environment from that happening in solution. As a result, the binding kinetic and thermodynamic data obtained in SPR may not reflect what happens in solution.^28^ Moreover, most previous studies have employed nanoparticles only as passive scaffolds to display polyvalent glycans to enhance MLGI affinity and/or specificity.^1−3,12,13^ However, their unique, size-dependent optical properties, the cornerstones of many nanomaterials, were not exploited as readout signals for MLGI affinity quantitation.

To address the above-stated limitations, we have recently demonstrated that small nanoparticles (*e.g*., ∼4 nm CdSe/ZnS core/shell quantum dots, QDs,^20,27,28^ and a ∼5 nm gold nanoparticle, GNP^14^) densely glycosylated with fragments of the natural high mannose structures are powerful probes for MLGIs. By harnessing the unique, size-dependent strong fluorescence (for QDs), or fluorescence quenching (for GNPs) properties, we have developed a robust and sensitive method for MLGI affinity quantitation based on the Förster resonance energy transfer (FRET, with QD) or fluorescence quenching (with GNP).^14,20^ We have further dissected the exact binding modes of the target MLGIs by analyzing the hydrodynamic size and capturing binding-induced nanoparticle–lectin assemblies under their native dispersion state by exploiting the nanoparticle’s size and high contrast under transmission electron microscopy (TEM) imaging.^14,20^ Using a pair of critically important, closely related tetrameric lectin viral receptors, DC-SIGN^34^ and DC-SIGNR,^35^ as model lectins, we have revealed that each DC-SIGN binds simultaneously to a single glycan-nanoparticle *via* all four of its CRDs, giving rise to strong affinities (low to sub-nM *K*_d_s) and forms small, isolated nanoparticle–lectin assemblies. In contrast, DC-SIGNR cross-links with multiple glycan-nanoparticles, resulting in extended large-scale nanoparticle–lectin assemblies and markedly weaker affinities compared to DC-SIGN.^14,20^ Moreover, we have found that these glycan-nanoparticles only potently and robustly block DC-SIGN-, but not DC-SIGNR-, mediated augmentation of cell entry of Ebola glycoprotein pseudotyped viruses, thus demonstrating the critical role of the MLGI binding mode of glycoconjugates in their ability to block cell surface lectin receptor-mediated viral infections.^14^

**Figure 1:**
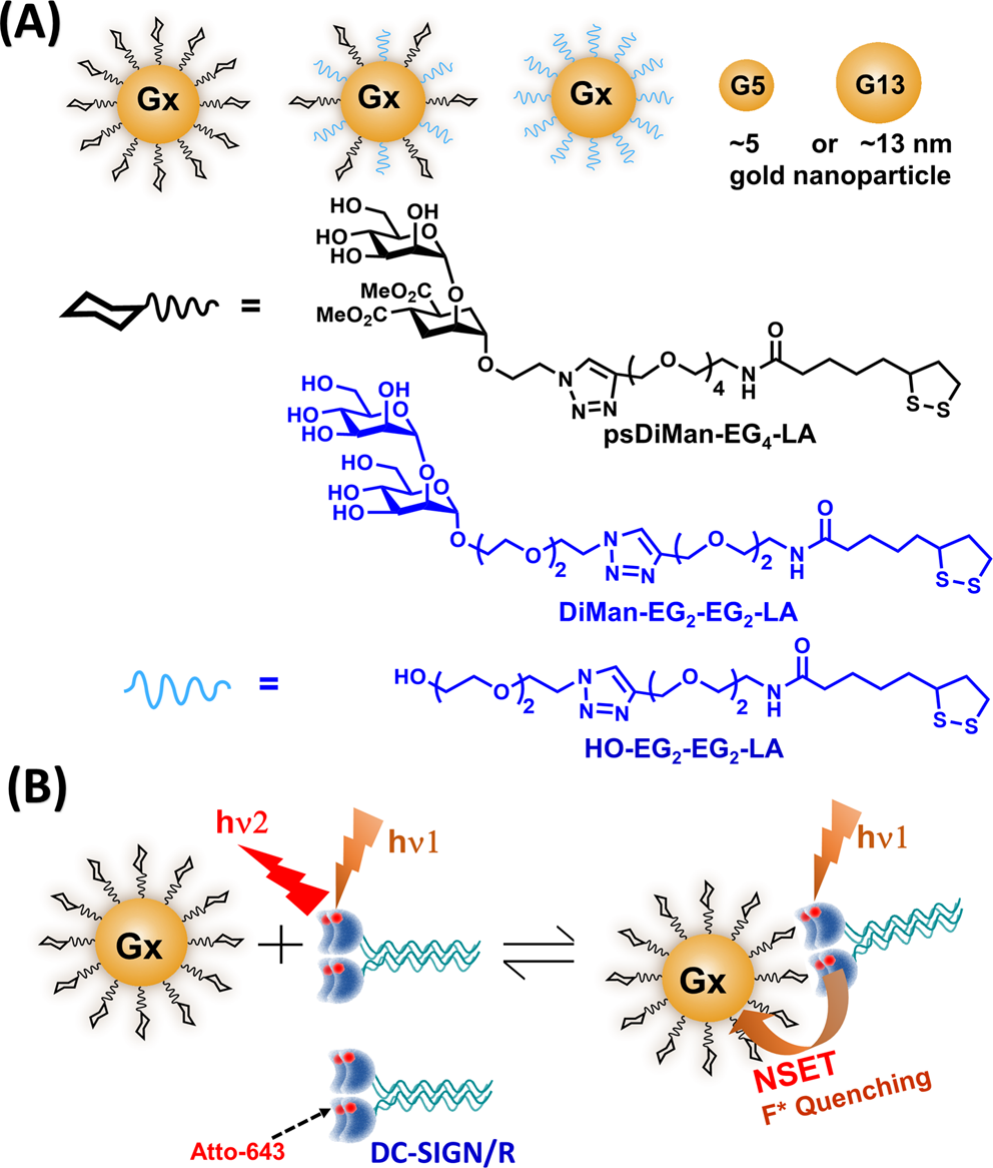
(A) Schematic of G*x*-psDiMan conjugates with varying glycan densities and GNP scaffold sizes. Chemical structures of LA–G_4_-psDiMan and LA-EG_2_-EG_2_-OH spacer ligands. The LA–EG_2_–EG_2_–DiMan ligand used in our previous studies is also included for easy structure comparison. (B) Schematic of the principle of the GNP fluorescence quenching assay for DC-SIGN/R-based MLGIs. Before binding, the Atto-643 labels on DC-SIGN/R give strong fluorescence upon excitation at 630 nm. Upon binding to G*x*-psDiMan, the Atto-643 fluorescence is efficiently quenched by GNP in proximity *via* the nano-surface energy transfer (NSET) mechanism, where the quenching efficiency is directly proportional to the percentage of DC-SIGN/R bound to G*x*-psDiMan. The strongly hydrophilic, bright, and red-emitting Atto-643 was selected as the fluorescent reporter to reduce any possible interference arising from GNP’s inner filter effect. Moreover, its fluorescence is insensitive to pH over the range of 2–11, allowing for robust measurement of binding-induced quenching while minimizing any interferences from environmental factors.

Despite these advances, how designing parameters, *e.g*., scaffold size, glycan type, and density, control their MLGI affinity, selectivity, and other key biophysical parameters remains to be revealed. To answer these questions, herein, we have displayed a synthetic glycomimetic, a pseudo-α-1,2-mannobioside (psDiMan) showing a different binding mode on DC-SIGN CRD from the natural high mannose fragment counterpart, α-manno-α-1,2-biose (DiMan),^36−38^ onto two different-sized GNP scaffolds (*e.g*., ∼5 and ∼13 nm in diameter, abbreviated as G5 psDiMan and G13 psDiMan, respectively) under systematically varying densities. psDiMan is designed by replacing the reducing end mannose of DiMan with a cyclohexanediol scaffold locked in a diaxial conformation by two carbomethoxy groups (see Figure 1 for structure comparison).^39^ The cyclohexane framework was found to offer specific hydrophobic interactions with Val351 in DC-SIGN, resulting in moderate selectivity toward DC-SIGN CRD over that of langerin, despite their sharing glycan specificity.^36^ We have quantified the apparent binding affinities between G*x*-psDiMan (*x* = 5 or 13) and DC-SIGN *via* GNP’s strong fluorescence quenching properties,^40−43^ revealing that G*x*-psDiMan binds strongly, with sub-nM apparent *K*_d_s, to DC-SIGN, which is enhanced with increasing GNP scaffold size. There is a minimal, GNP-size-dependent, glycan density threshold to form strong binding with DC-SIGN. In contrast, G*x*-psDiMans show apparently no (for G5) or very weak (for G13) binding to DC-SIGNR under the same conditions. A hydrodynamic diameter (*D*_h_) analysis of binding-induced G*x*-psDiMan-lectin assemblies reveals that each DC-SIGN molecule binds to a single G*x*-psDiMan *via* all four CRDs, irrespective of the GNP scaffold size, while DC-SIGNR binding is scaffold size-dependent: it shows no apparent binding with the smaller G5 psDiMan but weak cross-linking interactions with the larger G13 psDiMan. By applying the Van’t Hoff analysis of the temperature-dependent MLGI affinities between DC-SIGN and G*x*-psDiMan measured by fluorescence quenching, we reveal that DC-SIGN binding with both G5-/G13 psDiMan is enthalpy-driven, with comparable standard binding enthalpy changes (Δ*H*^0^s) being approximately four times that of the monovalent binding, suggesting that all four CRDs in each DC-SIGN molecule are engaged in binding. Their binding Δ*H*^0^ values also match those obtained from ITC. Finally, by employing vesicular stomatitis virus (VSV) particles pseudotyped with the glycoprotein (GP) of Ebola virus (EBOV_pp_), we have investigated the ability of G*x*-psDiMan to block DC-SIGN/R-promoted cellular entry of EBOV-GP_pp_.^14,20^ We reveal that G*x*-psDiMan can potently and robustly block DC-SIGN-, but not DC-SIGNR-, augmented viral entry to host cells, consistent with their different MLGI properties observed from GNP fluorescence quenching and dynamic light scattering. Together, these results have established G*x*-psDiMan as a powerful new biophysical tool for probing MLGI affinity, specificity, and thermodynamical mechanisms, allowing us to reveal the critically important role of the GNP scaffold size and glycan display in determining glycan-nanoparticles’ MLGI affinity, specificity, and antiviral properties against lectin receptors with distinct binding modes.

## 2 Results and Discussion

### 2.1 Preparation and Characterization of Essential Materials

#### 2.1.1 Design and Synthesis of LA-EG*_m_*-Based Ligands

A lipoic acid-tetra(ethylene glycol)-based multifunctional glycan ligand, LA-EG_4_-psDiMan, was designed. It contains three unique functional domains, a LA group for strong anchoring on the GNP surface by forming two strong Au–S bonds;^14,44^ a flexible tetra(ethylene glycol) linker to afford the terminal glycan with some flexibility and impose high water solubility, stability, and resisting nonspecific interactions;^45,46^ and a terminal pseudo-α-1,2-mannobioside (psDiMan) for specific binding with DC-SIGN.^36^ In addition, a LA-EG_2_ ligand containing an EG_2_-OH terminal group, abbreviated as LA-EG_2_-EG_2_-OH, was also synthesized as an inert spacer ligand to tailor the GNP surface glycan density (see Figure 1). This is because self-assembled monolayers terminated with oligo(ethylene glycol) groups are well-known for their excellent resistance against nonspecific adsorptions and nonspecific interactions with biomolecules.^45,47^ Both the LA-EG_4_-psDiman and LA-EG_2-_EG_2_-OH ligands contain the same LA-based GNP surface anchoring group with the same overall EG-linker length; therefore, they should have the same GNP anchoring and surface display properties. As a result, the ligand contents anchored onto the GNP surfaces should be the same as those used in solution self-assembly, allowing us to readily tune GNP surface glycan content by simply varying the glycan/spacer ligand ratio (but under a fixed total ligand:GNP ratio) used in the GNP–glycan preparation.

**Scheme 1:**
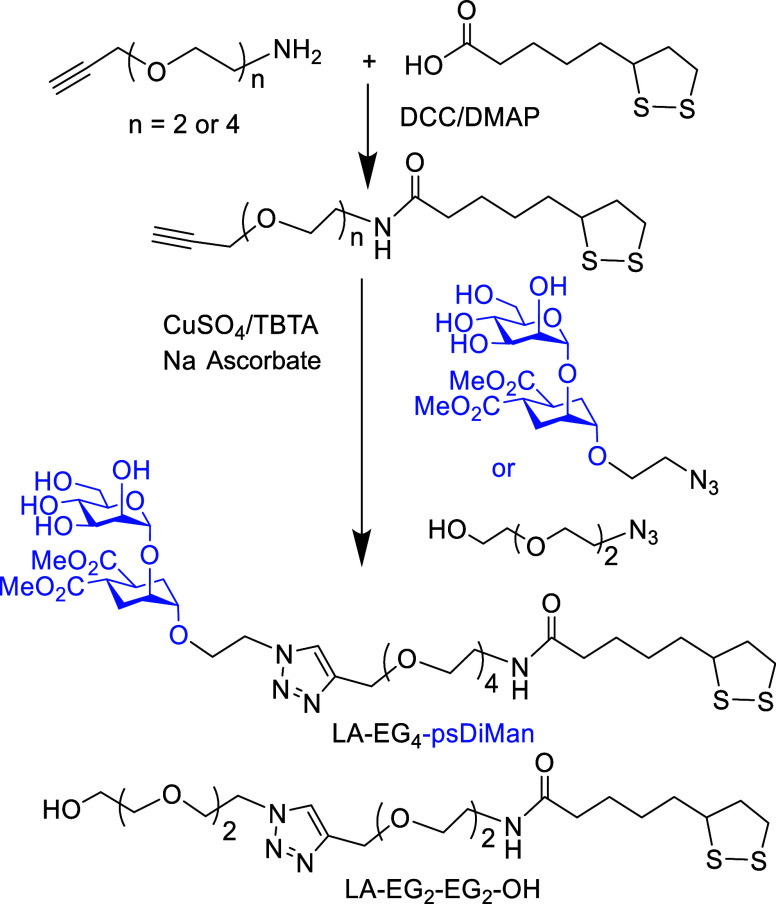
Synthetic Route to LA-EG_4_-psDiMan and LA-EG_2_-EG_2_-OH Ligands Used in This Study

The LA-EG_4_–psDiMan glycan and LA-EG_2_-EG_2_-OH spacer ligands were synthesized using the route shown schematically in Scheme 1. Briefly, lipoic acid was first coupled to the commercial H_2_N-EG*_m_*-C≡CH (*m* = 2 or 4) *via* dicyclohexylcarbodiimide/4-*N*,*N*-dimethylaminopyridine-mediated amide coupling to give the LA-EG*_m_*-C≡CH linker molecules in good yields, *e.g*., 72% for *m* = 2 and 85% for *m* = 4.^14,48^ psDiMan appending an α-(CH_2_)_2_-N_3_ linker in the pseudoanomeric position (psDiMan-C_2_-N_3_) was synthesized as described previously.^49^ Finally, LA-EG*_m_*-C≡CH was coupled to psDiMan-(CH_2_)_2_-N_3_ (*m* = 4) or commercial HO-EG_2_-N_3_ (*m* = 2) *via* the copper-catalyzed click reaction in the presence of CuSO_4_, sodium ascorbate (for reducing Cu^2+^ to Cu^+^), and tris(benzyltriazolylmethyl)amine (TBTA, for stabilizing the formed Cu^+^ catalyst).^14^ The crude products were purified by size exclusion chromatography using a Biogel P2 column *via* our established protocols^14,48^ to give the desired LA-EG_4_-psDiMan or LA-EG_2_-EG_2_-OH ligand in ∼80 or 85% yield, respectively. Their chemical structures were confirmed by their ^1^H/^13^C NMR and liquid chromatography–mass spectrometry (LC–MS) spectra (see Figures S1 and S2).

#### 2.1.2 Preparation and Characterization of G*x*-psDiMan

Two different-sized GNPs, with diameters of ∼5 and ∼13 nm (denoted as G5 and G13, respectively) were prepared by citrate reduction of H[AuCl_4_] in the absence (for G13) or presence of a small amount of tannic acid (for G5) by following the literature methods.^50−52^ Their core sizes were confirmed by TEM (Figure S3). They were then incubated with the LA-EG_4_-psDiMan ligand in an aqueous solution under total ligand:GNP molar ratios of 1000 and 3000 for G5 and G13, respectively, used to prepare the desired G*x*-psDiMan conjugates (*x* = 5 or 13). We have found previously that treating G5 with 1000 mol equiv of LA-EG*_n_*-glycan ligand produced highly stable and densely glycosylated G5-glycans.^14^ Here, there is a higher ligand/GNP molar ratio of 3000:1, about 2.4 times the ligand ratio required to coat the G13 surface with a full self-assembled monolayer of LA-EG*_n_*-ligand, ensuring that G13 was fully coated with the desired glycan ligands (see Supporting Information, Section 2.3).

To investigate how GNP surface glycan density affects their MLGI properties with DC-DIGN/R, the GNPs were further incubated with mixed LA-EG_4_-psDiMan and LA-EG_2_-EG_2_-OH ligands of varying ratios (but under a fixed total ligand/GNP molar ratio as above). In this way, a series of G*x*-psDiMan conjugates with the psDiMan content being systematically varied from 0, 6.3, 12.5, 25, 50, 75 to 100% were prepared (see Table 1). Each LA-based ligand can form two Au–S bonds upon self-assembly on the GNP surface, which would yield a total bond enthalpy of ∼90 kcal·mol^–1^,^53^ comparable to that of a typical C–C covalent single bond (83 kcal·mol^–1^). As a result, the LA-based ligands self-assembled on the GNP surface are expected to be nonmobile. Therefore, all of the LA-glycan and LA-spacer ligands should be randomly distributed on the GNP surface without phase separation. Moreover, given the fact that LA-EG_4_-psDiMan and LA-EG_2_-EG_2_-OH spacer ligands have the same surface anchoring group and the same overall EG_4_-linker length, the GNP surface glycan contents can be readily tuned by varying the glycan and spacer ligand molar ratio used in solution self-assembly.

**Table 1:** Summary of the Key Parameters of G*x*-psDiMan Conjugates under Different Glycan Densities^a^

G*x*	psDiMan (%)	*D*_h_ (nm)	*N*	*X* (nm)	apparent *K*_d_ (nM)	hill coefficient, *n*	β	β/*N*
G5	0	12.9 ± 2.4	0	-	-	-	-	-
	6.3	9.3 ± 2.4	30 ± 3	3.4 ± 0.2	-	-	-	-
	12.5	9.6 ± 2.1	60 ± 5	2.5 ± 0.1	-	-	-	-
	25	11.8 ± 2.5	119 ± 11	2.2 ± 0.1	2.74 ± 0.24	0.47 ± 0.02	400,000	3400
	50	11.8 ± 2.4	238 ± 22	1.53 ± 0.07	1.38 ± 0.26	0.54 ± 0.04	800,000	3300
	75	12.9 ± 2.2	357 ± 32	1.37 ± 0.06	0.68 ± 0.07	0.46 ± 0.07	1,600,000	4500
	100	11.4 ± 2.3	476 ± 43	1.05 ± 0.05	0.22 ± 0.05	0.37 ± 0.02	5,000,000	10,500
G13	0	16.7 ± 3.2	0	-	-	-	-	-
	6.3	18.1 ± 2.8	124 ± 12	3.3 ± 0.2	11.5 ± 0.4	0.74 ± 0.02	96,000	770
	12.5	19.5 ± 3.3	245 ± 23	2.5 ± 0.1	0.55 ± 0.03	0.54 ± 0.02	2,000,000	8200
	25	18.1 ± 2.9	491 ± 47	1.6 ± 0.1	0.30 ± 0.02	0.57 ± 0.03	3,700,000	7500
	50	18.9 ± 3.7	982 ± 93	1.21 ± 0.06	0.13 ± 0.02	0.55 ± 0.04	8,500,000	8700
	75	18.2 ± 3.0	1472 ± 140	0.95 ± 0.04	0.15 ± 0.02	0.47 ± 0.02	6,700,000	4500
	100	18.4 ± 3.6	1963 ± 186	0.83 ± 0.04	0.21 ± 0.02	0.59 ± 0.02	5,200,000	2700

a *D*_h_ = Hydrodynamic diameter (mean ± 1/2 FWHM); *N* = glycan valency per GNP; and *X* = average interglycan distance. Apparent *K*_d_ and *n* values were obtained by fitting the QE–concentration plots using eq 2 with a fixed Q*E*_max_% = 100 (*R*^2^ > 0.995 for all fits); “-” indicates binding curves not fitted due to binding being too weak; multivalent enhancement factor, β = *K*_d_^mono^/*K*_d_, where *K*_d_^mono^ = 1.1 mM (see Figure S13).

The successful preparation of G*x*-psDiMan conjugates was supported by a small increase of the hydrodynamic diameters (*D*_h_s) compared to their respective parent, citrate-stabilized G5 (*D*_h_ = ∼8.7 nm) and G13 (*D*_h_ = ∼15.3 nm) and the formation of monodispersed particles in water with narrow *D*_h_ distributions (see Figure S4 and data are summarized in Table 1). The resulting G*x*-psDiMan conjugates were found to be highly stable, and no changes of solution color or precipitation were observed after extended storage at 4 °C for >2 years. They exhibited a small red shift (*ca.* 4–6 nm) of plasmon absorption peak over their respective parent citrate-coated G*x* particles, due to a change of refractive index upon thiolated ligand-coating (Figure S4) and is fully consistent with the literature. No changes in plasmon absorption peak position or shape were observed for G*x*-psDiMan after dispersion to a standard binding buffer (20 mM HEPES, 100 mM NaCl, 10 mM CaCl_2_, pH 7.8) compared to those in pure water (Figure S4A), indicating that coating of the LA-psDiMan ligands greatly enhanced GNP’s colloidal stability against salt-induced aggregation (citrate-stabilized GNPs aggregate readily upon addition of moderate salt contents, due to effective screening of electrostatic repulsions among the negatively charged citrate-stabilized GNPs). Furthermore, both G5- and G13 psDiMan exhibited the same plasmon absorption peaks and *D*_h_s in water after extended storage in a fridge for >2 years (Figure S5), demonstrating excellent long-term stability for G*x*-psDiMan conjugates. The concentrations of G*x*-psDiMans were estimated by the Beer–Lambert law using their maximal plasmon absorbance at ∼515 (for G5) and ∼520 nm (for G13) using extinction coefficients of 6.3 × 10^6^ (for G5) and 2.32 × 10^8^ M^–1^ cm^–1^ (for G13), respectively.^14,51^

The glycan valency on the G*x* surface was estimated from the difference in glycan ligand amounts between that added and that remained unbound in the postincubation supernatant *via* a phenol-sulfuric acid carbohydrate quantifying method as described previously (Figure S6),^14,20,54^ and the results are summarized in Table 1. By using the *D*_h_ and glycan valency of G*x*-psDiMan conjugates under different glycan contents, the average glycan footprint, deflection angle, and interglycan distance were estimated *via* the method first reported by the Mirkin group^55^ and summarized in Tables 1 and S1. By diluting the glycan content on the G*x* surface with increasing amount of the LA-EG_2_-EG_2_-OH spacer ligand, a systematically increasing glycan footprint, deflection angle, and interglycan distance were obtained, allowing us to probe how these factors control their MLGI properties with DC-SIGN/R. Interestingly, the average interglycan distances for G5 psDiMan-100% (∼1.05 nm) and G13 psDiMan (50–100%, ∼0.8–1.2 nm) are comparable to the majority of interglycan sequon distances (∼0.7–1.3 nm) found on gp160,^56^ the HIV surface heavily glycosylated trimeric glycoprotein, which mediates specific DC-SIGN binding and viral infection.

#### 2.1.3 Protein Production and Labeling with Atto-643

DC-SIGN/R forms stable homotetramers on the cell surface, mediated by the neck region coiled-coil formation. We and others have demonstrated previously that the extracellular domain of DC-SIGN/R faithfully maintains the tetramer structure and MLGI properties of the full-length proteins.^27,57^ Hence, DC-SIGN/R extracellular segments (named as DC-SIGN/R hereafter) were used to study their solution MLGI properties with G*x*-psDiMan. To facilitate sensitive fluorescence-based binding detection, the recombinant mutants DC-SIGN-Q274C and DC-SIGNR-R287C were expressed in *Escherichia coli* and purified using Sepharose–Mannose affinity chromatography as described previously.^14,27^ The purified proteins were site-specifically labeled with a maleimide-modified Atto-643 dye (named as labeled DC-SIGN/R) through the Michael addition between thiol and maleimide as described previously.^14,20^ The dye-labeling sites in both proteins are close to but do not sit in their CRDs’ glycan binding pocket,^37^ and thus dye-labeling does not affect the CRDs’ glycan binding properties as confirmed previously.^14,20,28^ Atto-643 was chosen here due to its high fluorescence quantum yield, excellent photostability, and strong hydrophilicity, thereby minimizing any potential interference with the CRD structure and glycan binding properties. Moreover, its fluorescence emission peaks at the far-red region of the visible spectrum (*e.g*., λ_EX_ = 630, λ_EM_ ∼ 660 nm), which can minimize (but not eliminate) the potential interference with fluorescence readout arising from the GNP’s inner filter effect, due to their strong plasmon absorption in the visible region, especially for large GNPs (GNP’s molar extinction coefficient roughly scales linearly with its volume). The success of protein production and Atto-643 labeling was confirmed from their respective high-resolution mass spectra (HR-MS), where an increase of molecular mass of 935 was observed for both DC-SIGN/R. Using the molecular mass peak areas of the labeled and unlabeled proteins, labeling efficiencies of ∼92 and ∼90% per protein monomer were obtained for DC-SIGN and DC-SIGNR, respectively (see Figures S7 and S8).

The recombinant wild-type DC-SIGN/R (neither cysteine mutation nor dye-labeling) was also expressed and purified to investigate its binding properties with G*x*-psDiMan using dynamic light scattering (DLS) and ITC. Protein concentrations were determined by the Beer–Lambert law using their ultraviolet (UV) absorbance at 280 nm and a tetramer extinction coefficient of 2.82 × 10^5^ M^–1^ cm^–1^ for DC-SIGN or 2.44 × 10^5^ M^–1^ cm^–1^ for DC-SIGNR, as reported previously.^20,27^

### 2.2 Quantifying G*x*-psDiMan-DC-SIGN MLGI Affinity and Thermodynamics *via* GNP-Based Fluorescence Quenching

To investigate how GNP size and glycan density affect their MLGI with DC-SIGN, we quantified their binding affinities using GNP’s strong fluorescence quenching properties.^40−42^ Here, varying concentrations of labeled DC-SIGN and G*x*-psDiMan were mixed under a fixed mole ratio of 1:1 in a binding buffer (20 mM HEPES, 100 mM NaCl, 10 mM CaCl_2_, pH 7.8) containing large excess of a nontarget serum protein, bovine serum albumin (BSA, 1 mg/mL), which serves to minimize any possible nonspecific interactions.^14^ It can also reduce nonspecific adsorption of proteins and/or G*x*-psDiMans on surfaces, which can be a major source of experimental errors for binding assays performed at low concentrations (10 nM or below).^58^ Moreover, serum proteins are of high abundance *in vivo*; therefore, this also makes the binding environments resemble more closely to real biological situations. The G*x*-psDiMan and labeled DC-SIGN samples were incubated in the binding buffer for 20 min at room temperature before their fluorescence spectra (from 650 to 800 nm) were recorded under a fixed λ_EX_ of 630 nm. Labeled DC-SIGN only samples (without G*x*-psDiMan) were also recorded under identical conditions, which serve as controls to determine the quenching efficiency (QE) at each concentration (*C*) *via*eq 1(14)
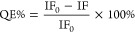
1where IF_0_ and IF are the integrated fluorescence of labeled DC-SIGN in the absence and presence of 1 mol equiv of G*x*-psDiMan, respectively. GNP can efficiently quench a wide range of fluorophores *via* a nano-surface energy transfer (NSET) mechanism (QE is proportional to the inverse fourth power of separation distance, *d*, *i.e*., QE *=* 1/[1 *+* (*d/d*_0_)^4^],^42^ where *d*_0_ is the distance giving 50% quenching). Fluorescence quenching *via* the NSET mechanism is more effective and covers a greater distance range than organic quenchers based on the Förster resonance energy transfer (FRET) mechanism, where QE is proportional to the inverse sixth power of dye–quencher distance, *R*, *QE =* 1/1 *+* (*R/R*_0_)^6^ and *R*_0_ is the Förster radius, under which QE = 50%.^41,42^ Moreover, a GNP has been shown to quench fluorescence by up to 99.97% in a closed DNA hairpin structure.^40^ Therefore, it is safe to assume that all GNP-bound lectins are fully quenched; hence, the measured QE% here represents the percentage of lectins that are bound to G*x*-psDiMan. Thus, the apparent binding equilibrium dissociation constant (*K*_d_) can be derived from the QE–concentration *(C)* relationship by fitting with Hill’s equation (eq 2)^14^
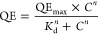
2where *QE*_max_, *K*_d_, *C*, and *n* are the maximum QE (fixed at 100%), apparent binding equilibrium dissociation constant, protein concentration, and Hill coefficient, respectively.

**Figure 2:**
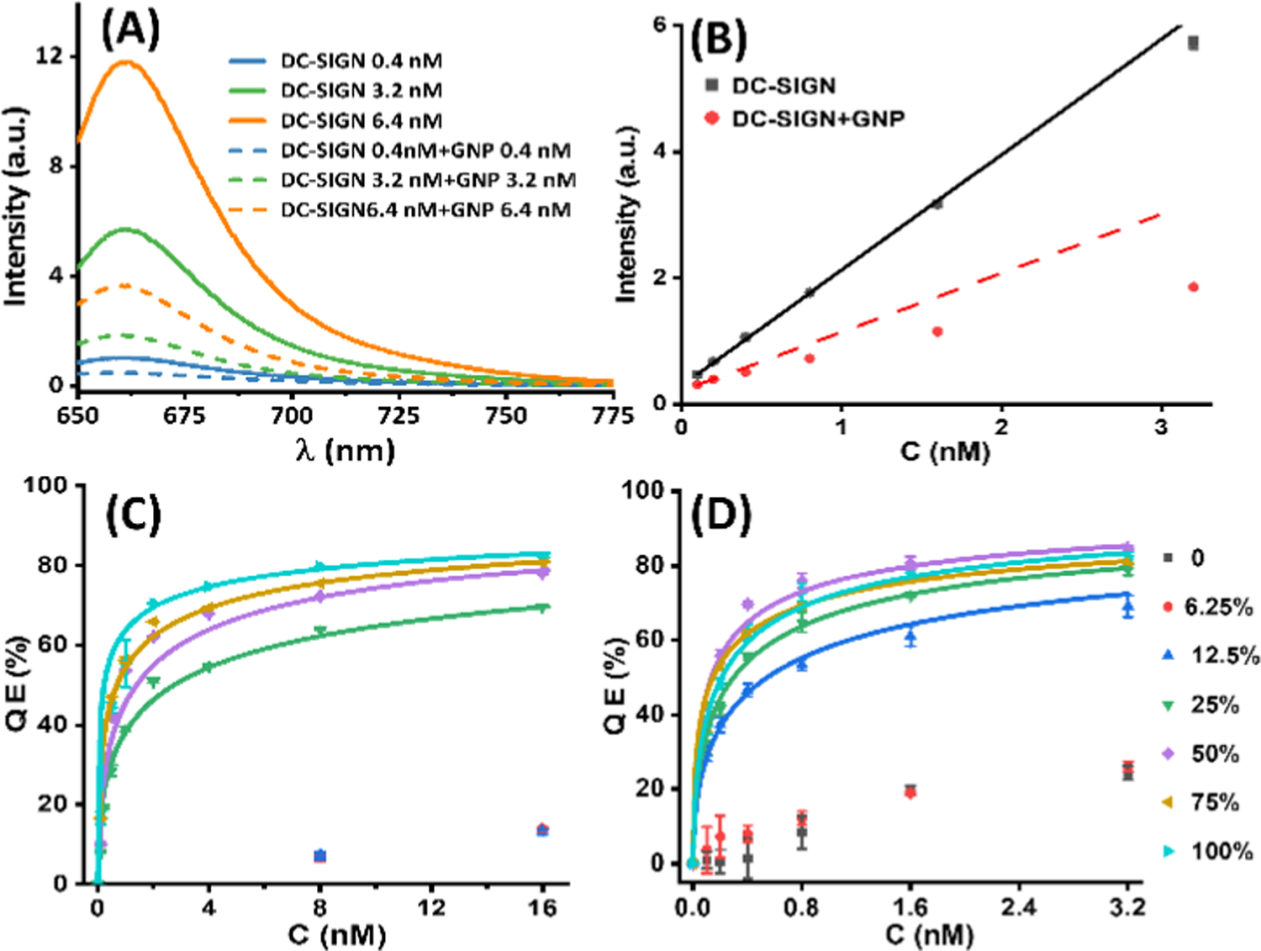
(A) Typical fluorescence spectra of Atto-643 labeled DC-SIGN (varying concentrations) in the absence (solid lines) or presence (broken lines) of 1 mol equivalent of G13 psDiMan-100%. (B) Integrated fluorescence intensity (IF)–concentration (*C*) plots for labeled DC-SIGN in the absence (black dots, with linear fit, *R*^2^ = 0.995) and presence (red dots) of G13 psDiMan-100%. (C, D) Plots of QE% *vs C* for labeled DC-SIGN binding with 1 mol equiv of G5 psDiMan (C) or G13 psDiMan (D) under a variety of glycan densities fitted by Hill’s equation (eq 2). The G5 psDiMan samples with 0, 6.25, and 12.5% glycan contents exhibited unexpected, small negative QEs (<−10%) at *C* ≤ 4 nM. Errors represent the standard experimental errors.

**Figure 3:**
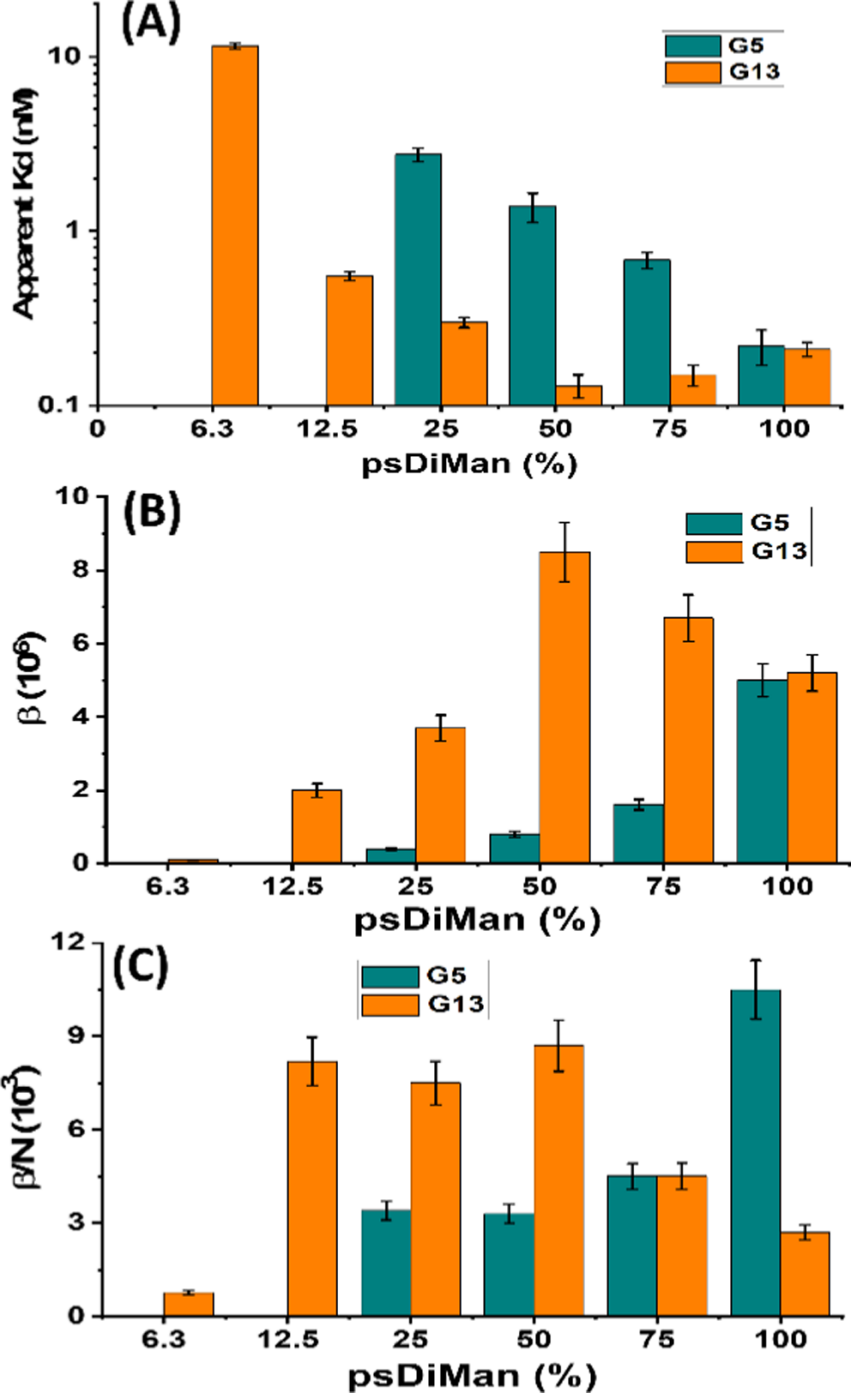
(A) Apparent binding *K*_d_s for DC-SIGN binding with G*x*-psDiMan (*x* = 5, yellow; *x* = 13, blue) as a function of surface psDiMan content%; (B) plots of multivalent affinity enhancement factor (β) or (C) per psDiMan normalized affinity enhancement factor (β/*N*) for G5 psDiMan (blue) or G13 psDiMan (yellow) binding with labeled DC-SIGN as a functional of surface psDiMan content% obtained by fluorescence quenching (DC-SIGN affinities for G5 psDiMan at psDiMan contents of ≤12.5% were too weak to measure accurately).

The representative fluorescence spectra of labeled DC-SIGN before and after mixing with G13 psDiMan (100% glycan density) at 1:1 molar ratio under different *C*s are shown in Figure 2A (fluorescence spectra showing the binding of G5 psDiMan with labeled DC-SIGN are given in Figure S9). The corresponding fluorescence spectra of DC-SIGNR binding with G*x*-psDiMan-50% and 100% or G*x*-OH controls are shown in Figure S10. It is apparent that labeled DC-SIGN fluorescence was greatly reduced in the presence of G13 psDiMan (or G5 psDiMan, Figures S9 and S10), especially at elevated concentrations. A plot of the integrated fluorescence (IF) *vs C* (Figure 2B) further revealed that, in the absence of G13 psDiMan, the fluorescence of DC-SIGN alone increased linearly (*R*^2^ > 0.995) with increasing *C*, while the presence of G13 psDiMan significantly and progressively quenched protein fluorescence, leading to the IF–*C* relationship deviating more and more from linear (Figure 2B). This result is fully consistent with the expectation that an increasing proportion of DC-SIGN would bind to G13 psDiMan and get quenched at elevated concentrations. The resulting QE–*C* relationships for DC-SIGN binding with G5 psDiMan and G13 psDiMan (with a variety of psDiMan contents) were fitted by Hill’s equation (eq 2) and are shown in Figure 2C,D, respectively. The detailed fitting parameters are summarized in Table 1. The relationships of the apparent *K*_d_, MLGI enhancement factor (β, where β = *K*_d_^mono^/*K*_d_ and *K*_d_^mono^ = ∼1.1 mM, obtained from ITC; see Figure S13), and per psDiMan normalized enhancement factor (β/*N*, where *N* is the valency of the psDiMan group on each GNP) as a function of the G*x* surface psDiMan content (%) are shown in Figure 3A–C, respectively.

In sharp contrast to DC-SIGN binding, the quenching of DC-SIGNR fluorescence by G*x*-psDiMan-50% and 100% was found to be minimal and was only observed at relatively high *C*s (*e.g*., 32 nM for G5 and ≥1.5 nM for G13; see Figure S11), where DC-SIGN quenching was already saturated (Figure 2C,D). In fact, quenching of DC-SIGNR fluorescence by G*x*-psDiMans was comparable to that of the corresponding G*x*-OH control showing no apparent binding to both lectins. Therefore, the weak quenching observed for DC-SIGNR here at high *C*s is mainly due to GNP’s inner filter effect rather than specific binding-induced quenching. This result is also consistent with the much lower *C*s for the G13-conjugates (*e.g*., 1.5 *vs* 32 nM) to exhibit observable quenching over their G5 counterparts, due to G13’s much stronger (∼37-fold) absorption extinction coefficient (hence inner filter effect) than G5 (*e.g*., 2.3 × 10^8^*vs* 6.3 × 10^6^ M^–1^cm^–1^). In contrast, we found previously that G5 capped with lectins’ natural DiMan ligand, LA-EG_2_-EG_2_-DiMan, exhibited significant binding (quenching) with DC-SIGNR, albeit still weaker than that with DC-SIGN, under such conditions.^14^ These results suggest that replacing the natural DiMan ligand with psDiMan on the G*x* surface capping significantly enhanced their MLGI selectivity for DC-SIGN over DC-SIGNR, a challenging task due to their close similarity in monovalent glycan binding and overall tetrameric architecture.

Based on the results of Figure 3 and Table 1, four conclusions can be drawn. (1) There is a minimal, GNP-size-dependent, psDiMan content threshold on the GNP surface in order to form strong DC-SIGN binding (*i.e*., sub- to low-nM *K*_d_s). The thresholds are 25 and 12.5% for G5 and G13 (denoted as G5 psDiMan25 and G13 psDiMan12.5%), respectively. (2) Above this threshold, DC-SIGN binding affinity increased gradually with increasing psDiMan content on G5 until reaching 100%. While for G13 psDiMan, the trend was less clear-cut: it gave the strongest DC-SIGN affinity with that capped with 50% psDiMan content (although the differences with those of 75 and 100% psDiMan contents were small and close to the assay detection limit), and further increasing or reducing the psDiMan content on the G13 surface led to slightly reduced affinity. (3) The highest DC-SIGN affinity was obtained with G13 psDiMan-50%, which gave an impressively strong apparent binding *K*_d_ of ∼0.13 nM. This affinity represents a massive, ∼8.5 million-fold MLGI affinity enhancement, β (= *K*_d_^mono^/ *K*_d_^MLGI^) over the corresponding monovalent psDiMan-DC-SIGN binding (*K*_d_^mono^ = 1.1 ± 0.3 mM, determined by ITC; see Figure S13) and per glycan normalized enhancement factor, β/*N*, of ∼8700. Moreover, G5 psDiMan-100% also exhibited an impressively strong MLGI affinity with DC-SIGN, with an apparent *K*_d_ of 0.22 ± 0.05 nM. This is ∼17-fold stronger than that of G5 coated with 100% LA-EG_2_-EG_2_-DiMan (*e.g.*, apparent *K*_d_ ∼ 3.8 nM),^14^ its equivalent natural DiMan ligand with the same total EG_4_ linker length, despite their comparable monovalent affinities (*e.g*., *K*_d_^mono^ 1.1 ± 0.3 *vs* 0.9 mM^59^). This result shows that displaying psDiMan polyvalently on a GNP surface is more effective in enhancing its MLGI affinity with DC-SIGN molecules in solution than that with DiMan, a natural glycan ligand for DC-SIGN, presumably due to their different binding motifs on DC-SIGN CRD.^36,60^ This result implies that we cannot directly use the relative strength of lectin–glycan monovalent affinity to predict their relative solution MLGI strengths involving polyvalent glycoconjugates. (4) The per psDiMan normalized enhancement factor, β/*N*, as a function of GNP surface psDiMan contents was found to depend strongly on the GNP size. For G5 psDiMan, its β/*N* generally increased with increasing psDiMan content and reached the maximum at 100% psDiMan; under which it gave a highly impressive β/*N* of ∼10,000. While for G13 psDiMan, its β/*N* broadly plateaued at ∼8000 as the psDiMan content increased from 12.5 to 50%; further increasing the psDiMan content led to a markedly reduced β/*N* value (Figure 3C). This result reveals a key role of surface curvature (scaffold size) of glycoconjugates in their ability to form strong MLGI with DC-SIGN.

The difference in the psDiMan density threshold for G5- and G13 psDiMan to form strong MLGI with DC-SIGN can be rationalized from the assumption that strong MLGIs are formed only when all four CRDs in DC-SIGN are engaged in binding. While the detailed crystal structure of the DC-SIGN tetramer remains unknown, the results from our group as well as others indicate that all four binding sites in DC-SIGN point upwardly in the same direction, allowing them to bind simultaneously to multiple glycans on the same G*x* surface.^14,20,27^ This was also confirmed from the *D*_h_ measurement of G*x*-psDiMan-100% + DC-SIGN samples under a variety of DC-SIGN: G*x*-psDiMan molar ratios, where only a single *D*_h_ species for both G5- and G13 psDiMan binding with DC-SIGN was observed. Their *D*_h_s initially increased with increasing DC-SIGN/G*x* ratio and then plateaued at a ratio of ∼6:1 or ∼32:1 for G5- or G13 psDiMan, respectively. This result indicates an increasing number of DC-SIGN molecules are bound to each G*x*-psDiMan before surface binding saturation. Moreover, the saturated *D*_h_s were found to be monodisperse and comparable to that expected for a central G*x*-psDiMan particle coated with a monolayer of DC-SIGN molecules (∼50–60 nm), implying that each DC-SIGN molecule must have bound to the central G*x*-psDiMan particle using all four of its CRDs (SI, Section 7 and Figures S15–S17). Given that the terminal psDiMans are displayed on the G*x* surface *via* a flexible EG_4_ linker, it is reasonable to assume that any psDiMan groups within the projected footprint of each CRD on the G*x* surface (∼7 nm^2^, based on a spherical CRD structure of ∼3 nm in diameter)^37^ could adapt and bind to that CRD. Therefore, any G*x*-psDiMan conjugates with a glycan footprint smaller than 7 nm^2^ are expected to be able to bind to all four CRDs in DC-SIGN, giving rise to strong MLGI affinity. This result matches well to the drastic increase of DC-SIGN MLGI affinity observed for G13 psDiMan as psDiMan content increased from 6.3 to 12.5% (*i.e*., average glycan footprint decreased from ∼9.4 to ∼4.9 nm^2^; see Table S1). The former psDiMan content is below the threshold required for all four CRDs in each DC-SIGN to engage in binding. For G5 psDiMan, a higher glycan content threshold is required in order to form strong tetravalent binding with DC-SIGN, presumably because its larger surface curvature has resulted in a glycan deflection angle being twice as large as that in its G13 psDiMan counterpart (*e.g*., 29.7 ± 1.3 *vs* 14.6 ± 0.7° for G5- *vs* G13 psDiMan12.5%; see Table S1), making its surface glycan ligands difficult to rearrange in order to fit all four glycan binding sites in each DC-SIGN molecule required to form strong binding.

### 2.3 Probing MLGI Thermodynamics by GNP Fluorescence Quenching in Comparison with Those Obtained with ITC

**Figure 4:**
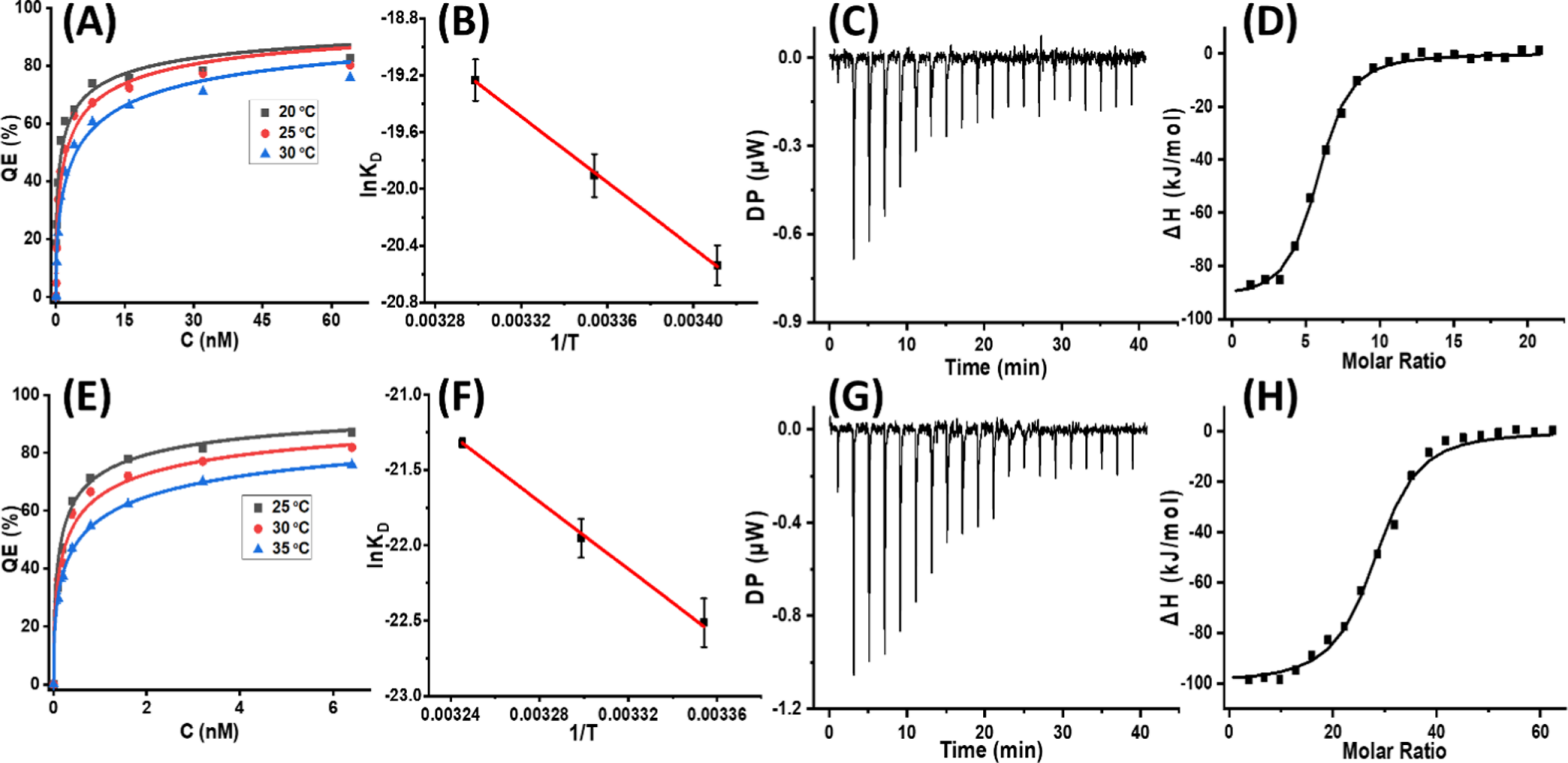
QE%–*C* relationships for DC-SIGN binding with G5 psDiMan-100% (A) or G13 psDiMan-100% (E) under three different temperatures fitted by Hill’s equation, and the corresponding Van’t Hoff plots (ln*K*_d_*vs* 1/*T* plots) for DC-SIGN binding with G5 psDiMan (B) or G13 psDiMan (F). ITC titration curves for wild-type DC-SIGN binding with G5 psDiMan-100% (C) or G13 psDiMan-100% (G) and their respective fitting curves (D, H). Error bars represent experimental errors.

Previously, we have probed the MLGI thermodynamics of DC-SIGN binding with QD-DiMan (∼4 nm CdSe/ZnS QDs coated with DHLA-EG_11_-EG_2_-DiMan ligands) by measuring their temperature-dependent affinities *via* a QD-FRET readout followed by Van’t Hoff analysis of their ln(*K*_d_)–(1/*T*) plots.^28^ We have revealed that DC-SIGN binding with QD-DiMan is enthalpy-driven with a Δ*H*^0^ of ∼ −100 kJ/mol, approximately four times that of the monovalent binding (Δ*H*_mono_^0^ = −25.8 kJ/mol),^59^ indicating that all four CRDs in each DC-SIGN are engaged in binding to QD-DiMan.^28^ To investigate whether the GNP fluorescence quenching assay can be exploited to probe the thermodynamics of high-affinity MLGIs, we further measured the apparent binding *K*_d_s between G5- or G13 psDiMan-100% and labeled DC-SIGN under three different temperatures. Both of their quenching efficiencies (QEs) were found to decrease with increasing temperature, indicating weakened interactions (larger *K*_d_s values). We then applied the Van’t Hoff analysis to derive their binding thermodynamics by combining the two Gibbs free energy equations (eqs 3 and 4).^28^ The changes of the standard binding enthalpy (Δ*H*^0^) and entropy (Δ*S*^0^) were obtained by taking a linear fit of the resulting ln(*K*_*d*_)–(1/*T*) plots, *via*eq 5 (Figure 4). The fitting results are summarized in Table 2.
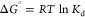
3
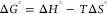
4
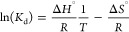
5where *R* is the ideal gas constant, 8.314 J·K^–1^·mol^–1^.

**Table 2:** Summary of the Standard Binding Thermodynamic Parameters (*T* = 298 K) between G*x*-psDiMan and DC-SIGN Obtained *via* the GNP Fluorescence Quenching Assay in Comparison with the Δ*H*^0^ Values Obtained by ITC^a^

	GNP fluorescence quenching assay			ITC
G*x*-psDiMan	Δ*H*^0^ (kJ·mol^–1^)	Δ*S*^0^ (J·K^–1^·mol^–1^)	Δ*G*^0^ (kJ·mol^–1^)	Δ*H*^0^ (kJ·mol^–1^)
G5 psDiMan-100%	–96.4 ± 2.6	–158 ± 9	–49.3 ± 2.7	–92.8 ± 1.6
G13 psDiMan-100%	–93.0 ± 3.2	–125 ± 11	–55.7 ± 3.3	–99.9 ± 1.7

a Errors represent the fitting errors.

In addition, we also measured the binding Δ*H*^0^s between G*x*-psDiMan-100% and wild-type DC-SIGN by isothermal titration calorimetry (ITC). These were performed by titrating concentrated DC-SIGN (30 μM) into concentrated G*x*-psDiMan solutions (*e.g*., 300 nM for G5 and 100 nM for G13) in the ITC cell to measure binding-induced heat changes (see SI Section 6I) and the results are summarized in Table 2. It should be noted that while ITC can provide accurate measurement of the binding Δ*H*^0^ values, it cannot provide direct accurate measurement of the binding Δ*G*^0^ (hence *K*_d_) for very strong interactions (e.g., sub- to low-nM *K*_d_s).^31,33^ This is another limitation of the ITC method in addition to its relatively low sensitivity and hence its requirement of large sample sizes.

The binding Δ*H*^0^ values for DC-SIGN binding with both G5-/G13 psDiMan obtained from the GNP fluorescence quenching assay were found to be in the same range as those obtained by ITC (Table 2), confirming that the GNP fluorescence quenching assay can be harnessed as a reliable method for probing the thermodynamics of high-affinity MLGIs (sub-nM *K*_d_s), thereby addressing a limitation of the ITC. Specifically, both G5- and G13 psDiMan-100% binding with DC-SIGN were found to be enthalpy-driven and exhibited both negative Δ*H*^0^ and Δ*S*^0^ values, indicating favorable binding enthalpy but unfavorable entropy terms (Table 2). Interestingly, the Δ*H*^0^ values of DC-SIGN binding with both G5 and G13 psDiMan are comparable, both at ∼ −95 KJ mol^–1^ (Table 2), suggesting that binding Δ*H*^0^ is not the determining factor for the observed MLGI affinity-GNP size-dependence. Moreover, such MLGI Δ*H*^0^ values are about four times that of psDiMan-DC-SIGN monovalent binding obtained from ITC (*e.g*., −23.4 kJ mol^–1^; see Figure S13), indicating that all four CRDs in each DC-SIGN are engaged in binding to G*x*-psDiMan, the same behavior as that observed for DC-SIGN binding to QD-DiMan.^28^ This result is consistent with the hydrodynamic diameters (*D*_h_s) of the G*x*-psDiMan-lectin complexes measured by dynamic light scattering, which gave saturated *D*_h_s of ∼50 and ∼57 nm for G5- and G13-complexes, respectively (Figures S16 and S17). Such *D*_h_s values roughly match those expected for single G*x*-psDiMan particles bound with a monolayer of DC-SIGN molecules, indicating that each DC-SIGN molecule binds tetravalently, *via* its all four CRDs, to a single G*x*-psDiMan, *i.e*., the same binding mode as that observed for DC-SIGN binding with G5-DiMan, previously.^14^

ITC studies on DC-SIGN binding to Gx-psDiMan-50% also gave similar binding Δ*H*^0^ values (*e.g*., −99.4 ± 2.7 and −93.6 ± 1.5 kJ/mol for G5 and G13, respectively; see Figure S14) to those of G*x*-psDiMan-100%, indicating the same tetravalent binding mode for DC-SIGN binding to G*x*-psDiMan-50% and 100%. This result is fully consistent with the glycan-content-dependent DC-SIGN binding affinity studies described in the previous section (Figure 2 and Table 1). Since the psDiMan contents in both G*x*-psDiMan-50% and 100% are higher than the minimal glycan density threshold, all four binding sites in each DC-SIGN molecule should be able to engage in binding with psDiMan groups from the same G*x*-psDiMan particle to yield the maximal binding valency.

Further analysis of Δ*S*^0^s for DC-SIGN-G*x*-psDiMan-100% binding revealed an interesting GNP scaffold size-dependence. The smaller G5 gave a larger negative Δ*S*^0^ than its larger G13 counterpart, *e.g*., −158 ± 9 *vs* −125 ± 11 J·K^–1^·mol^–1^, suggesting that increasing the GNP scaffold size reduces its DC-SIGN binding entropy penalty. Thus, the enhanced MLGI affinity obtained with the larger GNP scaffold originates from a reduced binding entropy penalty and not from an enhanced binding enthalpy. This is reasonable because our thermodynamic assays were performed under a lectin/G*x*-psDiMan molar ratio of 1:1, which is far below the surface binding saturation for G*x*-psDiMan. The larger GNPs thus have more unbound free glycan ligands on their surfaces than do the smaller ones. Such free glycan ligands still retain their native freedom of movement and hydration states after DC-SIGN binding, leading to a smaller binding entropic penalty for the larger GNPs over their smaller counterparts. Given that their binding Δ*H*^0^ values are comparable, this would yield a higher negative binding Δ*G*^0^ value and hence a stronger MLGI affinity for the larger G*x*-psDiMan over its smaller counterpart. To improve the potency of drugs and/or therapeutic interventions, it is important to enhance the drug–target binding affinities, which can be achieved by maximizing their favorable binding enthalpy terms while reducing unfavorable entropic terms. Our results show that creating a suitable multivalent display on a large scaffold could provide a potentially suitable solution.

### 2.4 G*x*-psDiMan Inhibition of DC-SIGN/R-Promoted EBOV_pp_ Cell Entry

To investigate whether the binding between DC-SIGN/R and G*x*-psDiMan in solution faithfully replicates their binding at the cell surface, we further investigated the ability of G*x*-psDiMan to block DC-SIGN/R-promoted cellular entry of vesicular stomatitis virus (VSV) particles pseudotyped with the Ebola virus glycoprotein (EBOV_pp_). The specific binding of the Ebola virus glycoprotein (EBOV-GP) to cell surface DC-SIGN/R receptors promotes viral attachment and entry into host cells, which ultimately leads to infection. Binding of high-affinity G*x*-psDiMan to cell surface DC-SIGN/R should prevent them from being able to bind EBOV-GP, thereby blocking virus cellular entry and infection.^13,14,21^ Compared to other antiviral strategies, the use of entry inhibitors to block viral infection can be advantageous since this can minimize virus developing resistance.^13,14,21^ Here, HEK293T cells transfected to express full-length DC-SIGN/R and single-cycle EBOV_pp_ encoding the luciferase gene were employed to evaluate the antiviral properties of G*x*-psDiMan-50% and 100% (*x* = 5, 13) as described previously.^14,20^ The experiments were performed in DMEM medium supplemented with 10% feta bovine serum (FBS) as before.^14,20^ The unprocessed inhibition data (luciferase activities) for each experiment together with their negative controls are given in Figures S21 and S22. The normalized inhibition data (after correction of the background from control VSV particles encoding no viral glycoprotein, named as Mock in Figures S21 and S22) were fitted by a modified inhibition model as shown in eq 6(14,48)
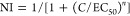
6where NI, *C*, EC_50_, and *n* are the normalized infection, G*x*-psDiMan concentration, concentration giving 50% apparent inhibition, and inhibition coefficient (with *n* >, =, and < 1 indicating positive-, non-, and negative-inhibiting cooperativity, respectively).^48^ While EC_50_ is a key indicator and widely used to assess the potency of antivirals, the inhibition coefficient “*n*” is much less mentioned in the literature. However, “*n*” is also of great importance for antivirals: it indicates how quickly an inhibitor can achieve complete inhibition by increasing the concentration. For example, three inhibitors have the same EC_50_ but different “*n*” values, and the theoretical concentration required to inhibit 99% infection would be 9801, 99, and 9.9 times the EC_50_ value for *n* = 0.5, 1, and 2, respectively.^48^ Therefore, antivirals displaying “*n”* ≥ 1 (with *n* = 1 being the most widely observed in the literature) are much more effective inhibitors than those having *n* < 1, allowing them to achieve complete inhibition at reasonable concentrations.

As shown in Table 3, both G5- and G13 psDiMan-50% and 100% potently blocked cell surface DC-SIGN-promoted cell entry of EBOV_pp_, with EC_50_ values being determined as 0.43 ± 0.17, 0.06 ± 0.03, 0.49 ± 0.13, and 0.18 ± 0.04 nM. Such low EC_50_ values place them among the most potent glycoconjugate inhibitors against DC-SIGN-augmented cell entry of EBOV_pp_ (*e.g*., the virus-like glycodendrinanoparticles, EC_50_: ∼0.91 nM, the giant globular glycofullerenes, EC_50_: ∼0.67 nM, and our previous QD-DiMan, EC_50_: ∼0.70 nM, and G5-EG_2_-EG_2_-DiMan, EC_50_: ∼0.095 nM).^2,12,14,18,20^ Moreover, a higher surface psDiMan content (*e.g*., G*x*-psDiMan-50% *vs* 100%) was found to benefit the antiviral potency for both G5- and G13 psDiMan. These results broadly agree with (but do not match exactly, especially for G13 psDiMan conjugates) their relative DC-SIGN binding affinities measured by GNP fluorescence quenching assay in solution. This may be due to the very different binding environments between those used in the fluorescence quenching assay (in solution with freely diffusing DC-SIGN molecules) and viral inhibition studies (on the cell surface with cell membrane-anchored DC-SIGN molecules with only very limited, in-plane mobility). Therefore, further binding studies using membrane-anchored lectin receptor models are still needed to help resolve such potential controversies.

**Table 3:** Summary of the Fitting Parameters (EC_50_, *n*, and *R*^*2*^) for G*x*-psDiMan Inhibition of DC-SIGN- or DC-SIGNR-Augmented Cell Entry of VSV Particles Pseudotyped with EBOV-GP^a^

	DC-SIGN			DC-SIGNR		
G*x*-psDiMan	EC_50_ (nM)	*n*	*R*^*2*^	EC_50_ (nM)	*n*	*R*^*2*^
G5 psDiMan-50%	0.43 ± 0.17	0.49 ± 0.07	0.960			
G5 psDiMan-100%	0.06 ± 0.03	0.53 ± 0.09	0.915			
G13 psDiMan-50%	0.49 ± 0.13	2.2 ± 0.7	0.978	3.1 ± 0.2	0.67 ± 0.04	0.993
G13 psDiMan-100%	0.18 ± 0.04	1.27 ± 0.26	0.981	3.7 ± 0.6	0.88 ± 0.11	0.982

a Errors represent the fitting errors.

Interestingly, both G5 psDiMan-50% and 100% displayed negative inhibition cooperativity (*n* = ∼0.5), while their G13 counterparts exhibited non- or even positive inhibition cooperativity (*n* ≥ 1, considering the relatively large fitting errors). Thus, a lower EC_50_ value for G5 psDiMan-100% over its G13-counterpart (*e.g.*, 0.06 ± 0.03 *vs* 0.18 ± 0.04 nM) does not necessarily mean that the former is a more effective antiviral than the latter. In fact, G13 psDiMan-100% at 3 nM has completely blocked DC-SIGN-promoted EBOV_pp_ cell entry (its luciferase activity ≤ background signal of the control VSV particle encoding no EBOV-GP gene), while its G5-counterpart has only blocked ∼80% of viral entry under the same concentration. The same trend was also observed for G5- and G13 psDiMan-50%. This result highlighted the importance of “*n*” in determining the efficiency of antivirals: both EC_50_ and “*n*” values should be considered together in order to obtain their true antiviral efficacy. This result also indicates that a large scaffold size is beneficial for the antiviral potencies of glycoconjugates-based entry inhibitors.^18^

The antiviral property of G5 psDiMan was found to be different from that of G5-DiMan (G5 coated with LA-EG_2_-EG_2_-DiMan, DC-SIGN’s natural DiMan ligand of the same overall EG-linker length). The former inhibition displayed negative cooperativity (*n* = ∼0.5), while the latter displayed non-cooperativity (*n* = 1).^14^ Such differences are likely due to the different binding motifs between DiMan and psDiMan in binding to DC-SIGN CRD. The crystal structure of the psDiMan-DC-SIGN CRD complex revealed that psDiMan uses a highly specific mode in binding to CRD: by coordinating to the CRD primary Ca^2+^ site *via* its intact mannose residue and forming multiple hydrophobic interactions with Val351 *via* its cyclohexane framework of the modified mannose.^36^ This binding mode is highly restricted and hence may require psDiMan to be presented at a specific orientation relative to the CRD in order to maximize binding contacts and affinity. However, DiMan can coordinate to the CRD primary Ca^2+^ site using either one of its two mannose residues, resulting in multiple binding modes of comparable affinity. This makes DiMan’s binding highly adaptable and can accommodate potentially a variety of CRD orientations to maintain comparable affinities.^37,60^ Each LA-based dithiol ligand can form two strong Au–S bonds on the GNP surface with an estimated total bonding enthalpy of ∼90 kcal·mol^–1^, similar to that of a typical single C–C covalent bond.^14,53^ The psDiMan ligands on the GNP surface should not be mobile apart from the flexibility offered by the EG_4_ linker. Here, the EG_4_ linker may still not be able to provide enough flexibility to fully compensate for the strict psDiMan orientation requirement on the highly curved G5 surface, allowing us to observe a seemingly contradicting relationship between MLGI affinity and viral inhibition properties for G5 psDiMan and G5-DiMan (*e.g*., a stronger binder being a worse inhibitor). For solution binding assays, both glycan-nanoparticles and DC-SIGN have total freedom of movement in all three dimensions, and thus they both can adapt to each other’s orientation preferences to maximize binding contacts and affinity, where G5 psDiMan binds more strongly to DC-SIGN than G5-DiMan does. Whereas in viral inhibition assays, DC-SIGN molecules are now anchored on cell membranes and are restricted to minor in-plane motions only, this makes them much less adaptable to meet the specific orientation demands of G5 psDiMan to achieve maximal binding and robust viral inhibition. In contrast, the highly flexible nature of G5-DiMan binding can still adapt to cell surface DC-SIGN molecules to achieve optimal binding and hence robust blocking of DC-SIGN-mediated viral entry.

**Figure 5:**
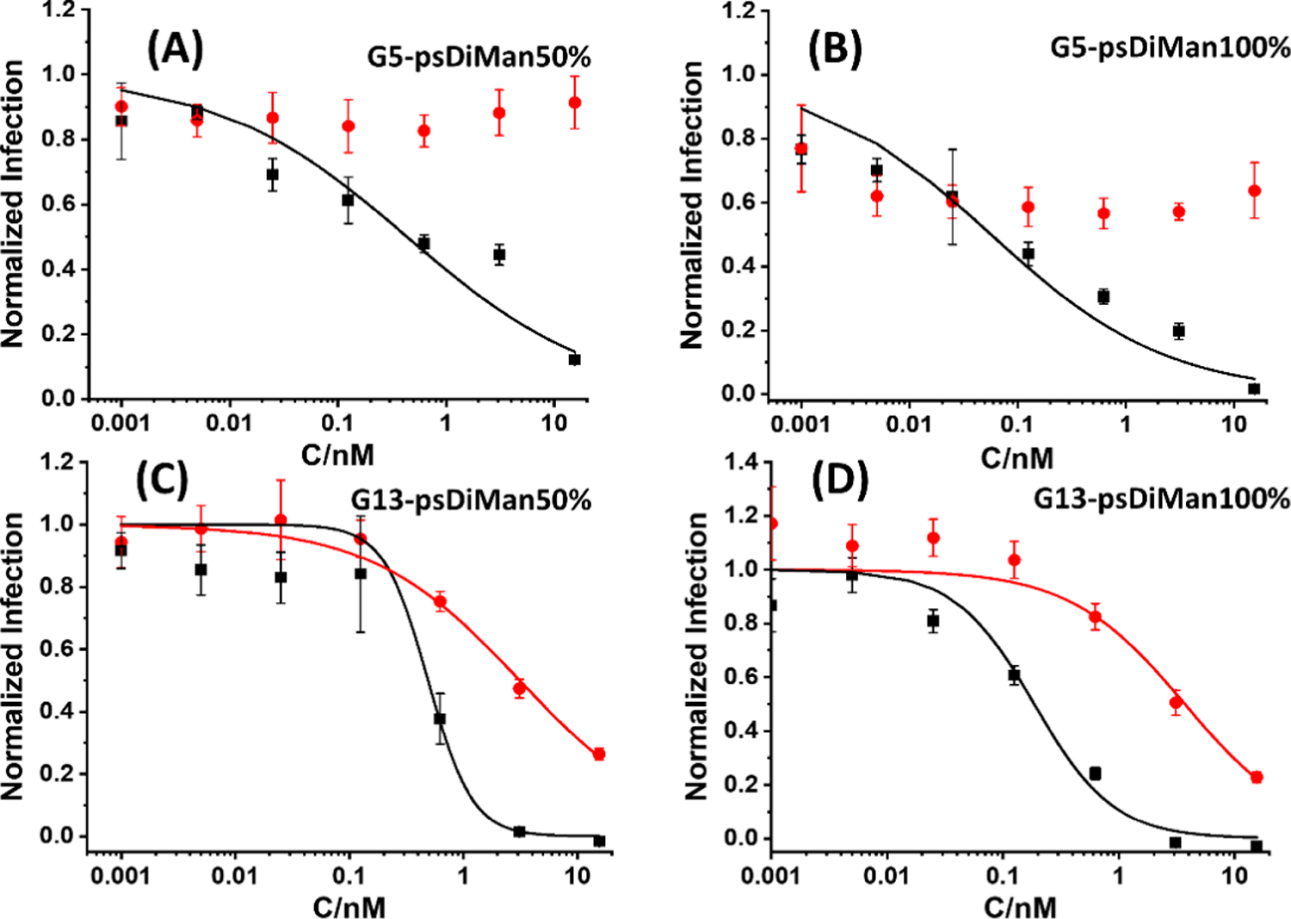
Plots of normalized infection (after background correction of the control particle encoding no viral glycoprotein) *vs* concentrations for G5 psDiMan-50% (A), G5 psDiMan-100% (B), G13 psDiMan-50% (C), and G13 psDiMan-100% (D) against DC-SIGN- (black squares) or DC-SIGNR- (red dots) augmented, EBOV-GP-driven entry into HEK293T cells fitted by eq 6. Error bars represent the standard experimental errors of a single experiment carried out in quadruplicate samples. Similar results were obtained in a separate experiment. The data for G5 psDiMan-50% and 100% were not fitted due to no significant inhibition. The detailed fitting parameters are summarized in Table 3.

Interestingly, the inhibition of DC-SIGNR-augmented EBOV_pp_ entry by G*x*-psDiMan was found to be strongly dependent on the GNP scaffold size, which contrasted sharply with those of DC-SIGN-medicated infections. Here, G5 psDiMan produced no significant inhibition across the whole concentration range studied, while G13 psDiMan gave notable, dose-dependent inhibition at higher concentrations (see Figure 5), albeit still less effective than that against DC-SIGN-promoted infections as evidenced by higher EC_50_ values and *n* < 1 (Table 3). Nonetheless, these results are fully consistent with their no apparent DC-SIGNR binding for G5 psDiMan or weak cross-linking interactions for G13 psDiMan observed in the GNP fluorescence quenching assay (Figure S11) and *D*_h_ analysis of binding-induced GNP–lectin complexes (where only G13 psDiMan, but not G5 psDiMan, exhibited observable cross-linking interactions with DC-SIGNR; see Figures S18 and S19). Together, these results have revealed a critical role of the GNP scaffold size toward G*x*-psDiMan’s MLGI affinities and antiviral properties: displaying psDiMan on the small G5 scaffold is highly beneficial for improving their MLGI selectivity for DC-SIGN and blocking DC-SIGN-augmented viral infections over those of DC-SIGNR, a closely related tetrameric lectin, whereas displaying on the large G13 scaffold can significantly enhance their MLGI affinities and antiviral potencies, but at the expenses of reduced selectivity. Importantly, no significant reduction of cell viabilities was observed for HEK293 cells after treatment of G*x*-psDiMan (*x* = 5 and 13) across the concentration range used in antiviral studies (Figure S20), suggesting that G*x*-psDiMan has good biocompatibility and is well-suited for potential biomedical applications.

## 3 Conclusions

In summary, by exploiting the versatile gold–thiol chemistry, tunable size, and powerful fluorescence quenching properties of GNPs,^40−42^ we have developed glycomimetic functionalized gold nanoparticles, G*x*-psDiMan, as a powerful new biophysical probe for MLGIs. We have found that displaying psDiMan polyvalently onto GNPs greatly enhances their MLGI affinities with DC-SIGN over monovalent binding (with β of ∼5 and ∼8.5 million-fold, and β/*N* of ∼10,500 and ∼8700 for G5 and G13, respectively). This MLGI affinity enhancement (β) is significantly greater (>20-fold, *e.g*., 5 million *vs* 2.3 × 10^5^ for G5 psDiMan *vs* G5-DiMan) than that observed with DiMan, its equivalent natural glycan ligand for DC-SIGN. We have revealed a critical role of GNP scaffold size in controlling their MLGI affinity and selectivity for DC-SIGN/R, two lectins with distinct binding modes, simultaneous tetravalent binding *vs* cross-linking. Where increasing the GNP scaffold size is highly beneficial for improving the MLGI affinity, this leads to reduced selectivity for DC-SIGN over DC-SIGNR, whereas reducing the scaffold size has the opposite effects. We have observed a minimal, GNP-size-dependent, psDiMan content threshold for G*x*-psDiMan in order to form strong MLGI with DC-SIGN, which can be rationalized by the CRD’s footprint. We have developed a new GNP fluorescence quenching assay for quantifying MLGI thermodynamics, revealing that G*x*-psDiMan-DC-SIGN binding is enthalpy-driven, with a binding Δ*H*^0^ of ∼ −95 kJ·mol^–1^, approximately four times that of the monovalent binding, implying that all four binding sites in each DC-SIGN are engaged in binding. Importantly, the binding Δ*H*^0^ values are comparable to those measured by ITC, thus verifying the credibility of our GNP fluorescence quenching method in probing high-affinity MLGI thermodynamics. We have also revealed that the enhanced MLGI affinity between DC-SIGN and G*x*-psDiMan with increasing scaffold size originates from a reduced binding entropy penalty and not from an enhanced binding enthalpy. We have further shown that G*x*-psDiMan can potently block cell surface DC-SIGN-augmented EBOV-GP-driven virus cellular entry with sub-nM to mid-pM level of EC_50_ values. Such low EC_50_ values place them among the most potent glycoconjugate inhibitors against DC-SIGN-mediated virus entry into host cells.^12−14,18,20^ Consistent with their solution MLGI affinities, G*x*-psDiMan exhibits no apparent or only weak inhibition against DC-SIGNR-promoted viral infection. Moreover, we have observed that GNP scaffold size is critical toward the antiviral properties of glycan-nanoparticles. The smaller G5 psDiMan shows negative inhibition cooperativity (*n* = ∼0.5), while the larger G13 psDiMan exhibits non- to positive-inhibition cooperativity (*n* ≥ 1). As a result, the latter has achieved complete inhibition at a lower concentration than the former, despite a higher EC_50_ value (*i.e*., 0.18 ± 0.04 *vs* 0.06 ± 0.03 nM). This result highlights the critical role of inhibition coefficient “*n”* in determining the efficiency and viability of glycoconjugate-based antiviral entry inhibitors.

## 4 Experimental Section

### 4.1 Ligand Synthesis and Characterization

LA-EG*_n_*-C≡CH linker molecules (*n* = 2 and 4) were synthesized by the standard dicyclohexylcarbodiimide/4-*N*,*N*-dimethylaminopyridine-mediated amide coupling between lipoic acid and H_2_N-EG*_m_*-C≡CH (purchased commercially) in dry CH_2_Cl_2_ in good yields, *e.g*., 72% for *n* = 2 and 85% for *n* = 4, as reported previously.^14,48^ psDiMan appending an α-(CH_2_)_2_-N_3_ linker in the pseudoanomeric position (psDiMan-C_2_-N_3_) was synthesized as described previously.^49^ The LA-EG*_n_*-C≡CH linker was then coupled to 1 mol equiv of psDiMan-(CH_2_)_2_-N_3_ (for *n* = 4) or commercial HO-EG_2_-N_3_ (for *n* = 2) *via* the copper-catalyzed click reaction in the presence of catalytic amounts of CuSO_4_ (0.05 mol equiv), sodium ascorbate (for reducing Cu^2+^ to Cu^+^), and tris(benzyltriazolylmethyl)amine (for stabilizing the Cu^+^ catalyst),^12^ using our established protocols.^14,48^ The crude products were purified by size exclusion chromatography *via* Biogel P2 column using 20 mM ammonium formate aqueous solution as an eluent, giving the desired LA-EG_4_-psDiMan and LA-EG_2_-EG_2_-OH ligands in ∼72 and ∼85% yields, respectively. Their ^1^H/^13^C NMR and LC-MS spectra are shown in Figures S1 and S2.

#### 4.1.1 LA-EG_4_-psDiMan

^1^H NMR (D_2_O, 500 MHz): δ = 8.14 (s, 1H, triazole-H), 4.97 (d, 1H, *J* = 1.8 Hz), 4.73 (d, 2H, *J* = 7.2 Hz), 4.66 (m, 1H), 4.03–3.94 (m, 3H), 3.91–3.85 (m, 2H), 3.81–3.68 (m, 18H, PEG repeats), 3.65–3.51 (m, 5H), 3.39 (t, 2H, *J* = 5.2 Hz), 3.28–3.15 (m, 2H), 2.86 (ddd, 1H, *J* = 13.0, 11.5, 3.7 Hz), 2.54–2.40 (m, 2H), 2.26 (t, 2H, *J* = 7.3 Hz), 2.10–1.92 (m, 3H), 1.75 (m, 2H), 1.69–1.46 (m, 4H), 1.42 (m, 2H) ppm. ^13^C NMR (D_2_O, 125 MHz): δ = 177.4, 177.1, 176.9 (3 × C=O), 125.6, 98.5, 73.7, 73.4, 70.8, 70.4 (2), 69.7, 69.6 (2), 69.5, 69.4, 68.9, 66.7, 66.5, 63.1, 61.0, 56.5, 52.5, 50.5, 40.2, 38.9, 38.8, 38.7, 38.0, 35.4, 33.7, 27.8, 26.7, 26.5, 25.0, ppm. LC-MS: calcd *m*/*z* for C_37_H_63_N_4_O_16_S_2_ (M + H)^+^, 883.37; found, 883.59.

#### 4.1.2 LA-EG_2_-EG_2_-OH

^1^H NMR (D_2_O, 500 MHz): δ = 8.01 (s, 1H, triazole-H), 4.62 (s, 2H), 4.65 (t, 2H, *J* = 5.0 Hz), 4.01 (t, 2H, *J* = 5.1 Hz), 3.76–3.60 (m, 13H, EG_*x*_ repeats), 3.55 (t, 2H, *J* = 5.3 Hz), 3.35–3.40 (m, 3H), 3.15–3.25 (m, 2H), 2.49 (dq, 1H, *J* = 12.3, 6.1 Hz), 2.24 (t, 2H, *J* = 7.2 Hz), 1.97 (dq, 1H, *J* = 13.6, 6.8 Hz), 1.57–1.75 (m, 4H), 1.40 (m, 2H) ppm. ^13^C NMR (D_2_O, 125 MHz): δ = 176.9 (C=O), 143.9, 125.5, 71.7, 69.7, 69.4, 69.3, 69.0, 68.9, 68.7, 63.1, 60.3, 56.5, 50.0, 40.2, 38.9, 38.0, 35.4, 33.7, 27.7, 25.0 ppm. LC-MS: calcd *m*/*z* for C_21_H_39_N_4_O_6_S_2_ (M + H)^+^, 507.23; found, 507.04.

### 4.2 Preparation of G*x*-psDiMan Conjugates

5 nm GNPs (G5s) were synthesized in-house using citrate reduction of HAuCl_4_ in the presence of a small amount of tannic acid by following a literature method.^52^ 13 nm GNPs (G13s) were synthesized by the standard citrate reduction method as reported previously.^50^ For G5 psDiMan conjugation, citrate-stabilized G5 was preconcentrated *via* centrifugation by 4000 rpm, 20 min using a 10 kDa MWCO filter. The concentrated G5 aqueous solution was then added to a ligand mixture of LA-EG_4_-psDiMan and LA-EG_2_-EG_2_-OH (with LA-EG_4_-psDiMan content varying from 100, 75, 50, 25, 12.5, 6.3, and 0%) under a fixed total ligand/G5 molar ratio of 1000:1 and incubated at room temperature for 48 h with shaking to make G5 psDiMan *via* self-assembly. Any unbound ligands were then removed by washing the G5 psDiMan conjugates with deionized water using a 10 kDa cutoff MWCO filter *via* centrifugation by 10,000*g*, 5 min, three times. The washing and flowthrough liquids were collected and combined to determine the amount of unbound LA-EG_4_-psDiMan ligand to calculate the GNP surface glycan valency.^14^

For G13 psDiMan conjugation, citrate-stabilized G13 was added to the mixed LA-EG_4_-psDiMan and LA-EG_2_-EG_2_-OH ligands (varying ratios as above) under a fixed total ligand/G13 molar ratio of 3000:1 in a glass vial. The mixture was sonicated for 2 min and then incubated for a further 48 h at room temperature to complete G13 psDiMan conjugation. After that, the G13 psDiMan conjugates were transferred to Eppendorf tubes pretreated by 0.2% Tween 20, centrifuged at 17,000*g*, 30 min, and washed with deionized water three times. The supernatant and washing liquids were collected to measure the amount of unbound LA-EG4 psDiMan ligands and calculate their glycan valency. The G*x*-psDiMan conjugates were dispersed in pure water and their concentrations were calculated from their SPR peak absorbance at ∼515 and ∼520 nm using molar extinction coefficients of 6.3 × 10 ^6^ and 2.32 × 10^8^ M^–1^cm^–1^ for G5 and G13,^14^ respectively.

### 4.3 Binding Studies *via* GNP Fluorescence Quenching Assay

To quantify the binding affinities between DC-SIGN and G*x*-psDiMan of varying psDiMan content (0–100%), Atto-643 labeled DC-SIGN (varying concentrations) was mixed with 1 mol equivalent of G*x*-psDiMan in a binding buffer (20 mM HEPES, 100 mM NaCl, 2 mM CaCl_2_, pH 7.8) containing 1 mg/mL BSA and then incubated for 20 min at room temperature. The protein concentration ranged from 0.1, 0.2, 0.5, 1, 2, 4, 8 to 16 nM for G5, or 0.1, 0.2, 0.4, 0.8, 1.6, 3.2 to 6.4 nM for G13, respectively. Fluorescence emission spectra were recoded over a range of 650–800 nm using a fixed λ_EX_ of 630 nm. Fluorescence spectra of labeled DC-SIGN at the above concentrations without G*x*-psDiMan were also recorded under identical conditions. The fluorescence spectra from 650 to 800 nm were integrated and used to calculate the quenching efficiency (QE) at each concentration (*C*) using eq 1. The obtained QE–*C* plots were fitted by Hill’s equation (eq 2) to derive their apparent binding *K*_d_s. For binding thermodynamic studies, DC-SIGN binding *K*_d_s with both G5- and G13 psDiMan conjugates were measured under three different temperatures using the same method as described above. Then, the obtained ln(*K*_d_) values were plotted against (1/*T*) and fitted by the linear function to obtain the slope and intercept, corresponding to Δ*H*^0^/*R* and −Δ*S*^0^/*R* for the DC-SIGN binding with G*x*-psDiMan conjugates.

### 4.4 Isothermal Titration Calorimetry (ITC) Assay

Wild-type DC-SIGN was dialyzed overnight against the binding buffer (20 mM HEPES, 100 mM NaCl, 2 mM CaCl_2_, pH 7.8) at 4 °C. The postdialysis buffer was stored at 4 °C for subsequent experiments, including preparation of all samples, control titrations, and rinsing the syringe and cell between each measurement. For psDiMan-DC-SIGN monovalent binding, psDiMan was dissolved in the binding buffer to obtain a final concentration of 50 mM. DC-SIGN was concentrated by centrifugal ultrafiltration (10 kDa MWCO filter) to obtain a final concentration of 15 μM. Isothermal titration calorimetry was performed using a MicroCal iTC200, with the psDiMan solution loaded into the syringe, and DC-SIGN loaded into the calorimeter cell. Titrations were conducted at 25 °C with an initial 0.5 μL injection, followed by 19 2 μL injections. A control experiment involving titration of psDiMan into the binding buffer (Figure S9B) was also recorded to measure the heat of dilution, which was then subtracted from the psDiMan-DC-SIGN binding titration to obtain the binding enthalpy change between psDiMan and DC-SIGN as shown in Figure S9(A). The standard MicroCal one set of sites model was used for fitting the plot of enthalpy changes, during which *N* (number of binding sites) was fixed at 4 as there are four CRDs on each DC-SIGN. The binding thermodynamic parameters were obtained as *K*_d_ = 1.1 ± 0.3 mM, Δ*H*^0^ = −23.4 ± 2.7 kJ·mol^–1^, Δ*G*^0^ = −17.0 kJ·mol^–1^, and Δ*S*^0^ = −21.5 J·K^–1^·mol^–1^.

For G*x*-psDiMan-DC-SIGN binding studies, the buffer of G5 psDiMan (100 and 50%) conjugates was exchanged three times with the postdialysis binding buffer using a 10 kDa cutoff centrifugal concentrator to obtain a final concentration of 300 nM. The buffer of G13 psDiMan conjugates was similarly exchanged with the postdialysis buffer using a 30 kDa cutoff centrifugal concentrator to obtain a final concentration of 100 nM. The DC-SIGN concentration used here was 30 μM. The DC-SIGN solution was loaded into the syringe, and G*x*-psDiMan solution was loaded into the calorimeter cell. A titration of DC-SIGN into a buffer was performed as a control titration. Enthalpy changes of G*x*-psDiMan binding to DC-SIGN were obtained by subtracting the average of the last 4–8 data points of the control titration, which have similar heat changes to correct the effect of heat dilution. The titration curve was fitted with the same method described above to obtain Δ*H*° values.

### 4.5 Virus Inhibition

The inhibition effects of G*x*-psDiMan (50% and 100%) on 293T cell entry of particles pseudotyped with the Ebola virus glycoprotein (EBOV_pp_) were assessed using our established procedures.^14,20^ Briefly, 293T cells seeded in 96-well plates were transfected with plasmids encoding DC-SIGN or the control transfected with empty plasmid (pcDNA). The cells were washed at 16 h posttransfection and further cultivated at 37 °C, 5% CO_2_ in Dulbecco’s modified eagle medium (DMEM) containing 10% fetal bovine serum (FBS). At 48 h posttransfection, the cells were exposed to twice the final concentration of G*x*-psDiMan inhibitor in OptiMEM-medium for 30 min in a total volume of 50 μL. Thereafter, the resulting cells were inoculated with 50 μL of preparations of vesicular stomatitis virus (VSV) vector particles encoding the luciferase gene and bearing either EBOV-GP (which can use DC-SIGN/R for augmentation of host cell entry) or the VSV glycoprotein (VSV-G), which cannot use DC-SIGN or DC-SIGNR for the augmentation of the host cell entry. Under these conditions, binding of G*x*-psDiMan particles to 293T cell surface DC-SIGN receptors can block EBOV-GP interactions with these lectin receptors, resulting in a reduced transduction efficiency of the virus particles and hence reducing the cellular luciferase activity. At 16–20 h postinfection, luciferase activities in cell lysates were determined using a commercially available kit (PJK), following the manufacturer’s instructions, as described in our previous publications.^14,20^

### 4.6 Cytotoxicity Assay

1 × 10^4^ HEK293 cells (1 × 10^4^) were seeded to each well in a 96-well plate. After 24 h, G5 psDiMan-50% and 100%, G5-OH control, G13 psDiMan-50 and 100%, and G13-OH were added to the wells sequentially to final concentrations of 3.1 and 15.5 nM (in triplicates for each sample), corresponding to the highest concentrations of G*x*-conjugates used in the viral inhibition assays. The cells added with the phosphate-buffered saline (PBS) buffer without any nanoparticles were used as the positive control. After incubation overnight, the medium was removed and cells were washed gently with PBS to remove any unbound nanoparticles. Then, 100 μL of 0.5 mg/mL MTT (in phenol red-free medium) was added to each well and incubated for 2.5 h at 37 °C. After incubation, free MTT and medium were removed, and 100 μL of DMSO was then added to each well to dissolve the formed formazan. After incubated for another 15 min at 37 °C, the absorbance at 550 nm was read on a CLARIOstar plate reader. The absorbance data were normalized (using the PBS control as 100) to assess the cytotoxicity of the G*x*-glycan conjugates. The data are shown in Figure S20.

## Supporting Information Available

Experimental details including materials and methods; synthesis procedures for LA-EG_4_-DiMan and LA-EG_2_-EG_2-_OH ligand, and their ^1^H/^13^C NMR and LC-MS spectra; determination of glycan conjugation efficiency and valency; surface footprint calculation; protein production; labeling and HR-MS characterization; fluorescence spectra of GNP quenching assays; ITC data; and the original data of viral inhibition studies (PDF)

## Abbreviations

GNP: gold nanoparticle
DC-SIGN: dendritic cell-specific intercellular adhesion molecule-3-grabbing nonintegrin
DC-SIGNR: DC-SIGN-related lectin found on endothelial cells
TLC: thin layer chromatography
HPLC: high-performance liquid chromatography
NMR: nuclear magnetic resonance
MS: mass spectrometry
ITC: isothermal titration calorimetry
DLS: dynamic light scattering
