# Supplementary material for: Polyvalent Glycomimetic-Gold Nanoparticles Revealing Critical Roles of Glycan Display on Multivalent Lectin–Glycan Interaction Biophysics and Antiviral Properties
Source: JACS Au. 2024 Aug 15;4(8):3295–309. doi: 10.1021/jacsau.4c00610 (PMC11350578; doi:10.1021/jacsau.4c00610)
Supplement: Supplementary file 1 — au4c00610_si_001.pdf [file au4c00610_si_001.pdf]

Supporting Information

Polyvalent Glycomimetic-Gold Nanoparticles Revealing Critical Roles of Glycan Display on Multivalent Lectin-Glycan Interaction Biophysics and Antiviral Properties

Xinyu Ning,[†,╬] Darshita Budhadev,[†,╬] Sara Pollastri[#] Inga Nehlmeier,[‡] Amy Kempf,[‡] Iain Manfield,[ɸ] W. Bruce Turnbull,[†] Stefan Pöhlmann[‡,$] Anna Bernardi,[#] Xin Li,[⁋] Yuan Guo[ɧ,]*, and Dejian Zhou[†,]*

[†] School of Chemistry and Astbury Centre for Structural Molecular Biology, University of Leeds, Leeds LS2 9JT, United Kingdom. Email: d.zhou@leeds.ac.uk.

[#] Dipartimento di Chimica, Universita` degli Studi di Milano, via Golgi 19, Milano 20133, Italy.

[‡] Infection Biology Unit, German Primate Center – Leibniz Institute for Primate Research, 37077 Göttingen, Germany.

[$] Faculty of Biology and Psychology, University of Göttingen, 37073 Göttingen, Germany.

[ɧ] School of Food Science & Nutrition and Astbury Centre for Structural Molecular Biology, University of Leeds, Leeds LS2 9JT, United Kingdom. Email: y.guo@leeds.ac.uk.

[⁋] Sphere Fluidics Ltd., Building One, Granta Centre, Granta Park, Great Abington, Cambridge, England, CB21 6AL, United Kingdom.

[ɸ] School of Molecular and Cellular Biology and Astbury Centre for Structural Molecular Biology, University of Leeds, Leeds LS2 9JT, United Kingdom.

[╬] These authors contributed equally to this work.

Contents

1. Materials, instruments and methods S2

2. Synthesis and characterization of glycan ligands, GNPs, and GNP-glycan conjugates S2-10

3. Determination of glycan valency, inter-psDiMan distance and deflection angle S11-12

4. Production and labeling of DC-SIGN and DC-SIGNR S13-14

5. Fluorescence quenching assays for determining MLGI affinity and thermodynamics S15-17

6. Isothermal titration calorimetry studies S18

7. Dynamic light scattering studies S19-22

8. Cytotoxicity assay S23

9. Viral inhibition studies S24-25

9. Supporting References S26

1. Materials, Instruments, and Methods

1.1 Materials

Tris-sodium citrate (99.5%), potassium carbonate anhydrous (K_2CO_3, >99%), sodium hydroxide (NaOH, 99%), sodium chloride (NaCl, ≥99.5%), calcium chloride ($CaCl_2$, ≥96%), 4-(2-hydroxyethyl)-1-piperazineethane-sulfonic acid (HEPES, ≥99 %), hydrochloric acid (HCl, ~37%), and sulfuric acid (H_2SO_4, >95%) were purchased from Fisher Scientific (U.K.). Tannic acid (99.5%), ascorbic acid (99%), nitric acid (HNO_3, 68-70%) and N_3-EG_2-OH (>98%) were purchased from Sigma Aldrich (U.K.). Hydrogen tetrachloroaurate trihydrate ($HAuCl_4 \cdot 3H_2O$, 99.9%) and phenol (>99.5%) were purchased from Alfa Aesar (U.K.). Bovine serum albumin (BSA, 99%) was purchased from BioServ (U.K.). H_2N-EG_4-C≡CH (>97%) and H_2N-EG_2-C≡CH (>99%) linkers were purchased from PurePEG LLC (CA, U.S.A.).https://www.purepeg.com/

Deionised water obtained from the ELGA Purelab classic UVF system (>18.2 mΩ cm) was used in making buffers and all experiments. A binding buffer (20 mM HEPES, 100 mM NaCl, 2 mM $CaCl_2$, pH 7.8) was used for protein storage, dialysis and ITC binding studies. The binding buffer (20 mM HEPES, 100 mM NaCl, 2 mM $CaCl_2$, pH 7.8) with 1 mg/mL BSA was used in all binding affinity and thermodynamic studies by GNP based fluorescence quenching.

1.2 Instruments and methods

Ultraviolet-visible (UV-vis) absorption spectra were recorded on a Cary 60 UV-vis Spectrophotometer over 200-800 nm using 1 mL disposable plastic cuvette (optical path length is 1 cm), or on a Thermo Scientific Nanodrop 2000 spectrophotometer using 1 drop of solution (optical path length is 1 mm). Centrifugations were performed on a Thermo Scientific Heraeus Fresco 17 microcentrifuge using 1.5 mL Eppendorfs and Sartorius Vivaspin 500 centrifugal concentrators (cut-off MWCO filter) at room temperature, or on a Hettich Universal 320 benchtop centrifuge using Merck Amicon centrifugal filter (cut-off MWCO filter). Dynamic light scattering (DLS) studies were performed on Malvern Zetasizer Nano in disposable cuvettes at 25°C. NMR spectra were recorded on a Bruker Avance DPX300 (500 MHz for ^{1}H, 125 MHz for ^{13}C) in deuterated solvents. High resolution mass spectra (HR-MS) were recorded on a Bruker Daltonics MicroTOF mass spectrometer. Fluorescence spectra were recorded on a Jobin Yvon FluoroMax 5 Spectrofluorometer in a 0.7 mL quartz cuvette, using a fixed excitation wavelength of 630 nm, and fluorescence emission spectra were recorded from 650 to 800 nm. For thermodynamics study, a Labnet digital dry bath incubator was used to maintain all samples' temperature during incubation, while the cuvette temperature was maintained by a circulating water bath. Isothermal titration calorimetry (ITC) measurement was recorded at 25°C on the MicroCal iTC200 isothermal titration calorimeter, and the data was analysed on Origin 5 software. All other data were analysed using Microsoft Excel and OriginPro 2019 and 2022 versions.

2. Synthesis of LA - psDiMan ligands, GNPs, and preparation of GNP-psDiMan conjugates

2.1 Synthesis of LA-EG_4-psDiMan and LA-EG_2-EG_2-OH[1, 2]

(1) LA-EG_4-C≡CH: H_2N-EG_4-C≡CH (2.00 g, 8.65 mmol), lipoic acid, **4** (1.80 g, 8.65 mmol) and DMAP (0.21g, 1.73 mmol) were added anhydrous CH_2Cl_2 (25 mL) and the reaction mixture was stirred at 0 °C under nitrogen for 15 min. After that, DCC (1.82g, 10.2 mmol) in anhydrous CH_2Cl_2 (5 mL) was added dropwise to the reaction mixture over 20 min. The resulting reaction was stirred at 0 °C for another hour before being allowed to warm to room temperature gradually and then stirred overnight. Upon completion, the reaction mixture was filtered over celite to remove the DCU byproduct and the solvent evaporated. The crude product was purified by flash silica chromatography ($CHCl_3$: CH_3OH = 10:1) to furnish the desired product, **LA-EG_4-C≡CH**, as a yellowish oil (3.08 g, yield 85%).[2]

^{1}H NMR ($CDCl_3$, 500 MHz): δ = 6.50 (t, 1H, *J*=5.7 Hz, amide N*H*), 4.00 (d, 2H, *J*=2.4 Hz), 3.52-3.45 (m, 8H), 3.45-3.30 (m, 7H), 3.24 (td, 2H, *J*=5.6, 4.5 Hz), 3.05 – 2.90 (m, 2H), 2.30 (s, 1H), 2.28 – 2.20 (m, 1H), 2.01 (t, 2H, *J*=7.5), 1.75 – 1.65 (m, 1H), 1.60 – 1.40 (m, 4H), 1.32 – 1.18 (m, 2H) ppm. ^{13}C NMR ($CDCl_3$, 125 MHz): δ 172.4 (C=O), 79.0, 74.4, 69.9(2), 69.8, 69.7, 69.5, 69.2, 68.4, 57.7, 55.8, 39.6, 38.6, 37.9, 35.6, 34.1, 28.3, 24.8 ppm. **HRMS**: calculated *m/z* for $C_{19}H_{34}NO_5S_2$ $(M+H)^+$ 420.1878; found 420.1873.

(2) LA-EG4-psDiMan:[1] To a 1:1 (v:v) mixed THF: H_2O solution (2.0) containing the psDiMan-$(CH_2)_2$-N_3 (20 mg, 0.040 mmol, synthesised via the method reported previously[3]) and LA-EG_4-C≡CH (18.1 mg, 0.040 mmol), was added $CuSO_4.5H_2O$ (0.4 mg, 1.6 μmol), TBTA (1.4 mg, 2.7 μmol) followed by sodium ascorbate (1.2 mg, 5.8 μmol) and the resulting solution was stirred at room temperature. After 6 hours, TLC confirmed the complete consumption of all starting materials. The organic solvent was evaporated and then it was freeze-dried to get rid of water. The crude product was purified by size exclusion chromatography via Biogel P2 column using 20 mM ammonium formate aqueous solution as an eluent. The fractions containing the pure product (checked with TLC) were combined and freeze-dried to afford the desired product, LA-EG_4-psDiMan, as a yellowish solid (23 mg, 72%).

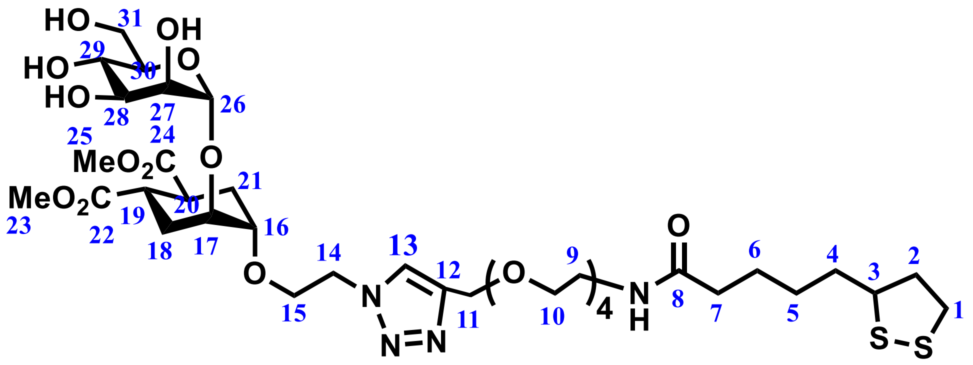

^{1}H NMR (D_2O, 500 MHz): δ = 8.14 (s, 1H, H13), 4.97 (d, 1H, *J*=1.8 Hz, H26), 4.73 (d, 2H, *J*=7.2 Hz, H14), 4.66 (m, 1H, H30), 4.03 – 3.94 (m, 3H, H27,28,29), 3.91 – 3.85 (m, 2H, H16,17), 3.81-3.68 (m, 18H, H23 & 25 and H10,9 in PEG repeats), 3.65 – 3.51 (m, 5H, H11,H14,H3), 3.39 (t, 2H, *J*=5.2 Hz, H15), 3.28 – 3.15 (m, 2H, H1), 2.86 (ddd, 1H, *J*=13.0, 11.5, 3.7 Hz, H2), 2.54 – 2.40 (m, 2H, H19, 21), 2.26 (t, 2H, *J*=7.3 Hz, H7), 2.10 – 1.92 (m, 3H, H18,H2), 1.75 (m, 2H, H6), 1.69 – 1.46 (m, 4H, H4,6), 1.42 (m, 2H, H5) ppm. ^{13}C NMR (D_2O, 125 MHz): δ = 177.4, 177.1, 176.9 (3x C=O), 125.6 (C12), 98.5 (C13), 73.7, 73.4, 70.8, 70.4 (2), 69.7, 69.6 (2), 69.5, 69.4, 68.9, 66.7, 66.5, 63.1, 61.0, 56.5, 52.5, 50.5, 40.2, 38.9, 38.8, 38.7, 38.0, 35.4, 33.7, 27.8, 26.7, 26.5, 25.0 (C5), ppm. LC-MS: calculated *m/z* for $C_{37}H_{63}N_4O_{16}S_2$ $(M+H)^+$ 883.37; found 883.59.

(3) **LA-EG_2-EG_2-OH** was synthesized using LA-EG_2-C≡CH (previously synthesised in house)[1] and HO-EG_2-N_3 (commercial) *via* the Cu-catalyzed click reaction and purified using the same method as above in ~85% yield.

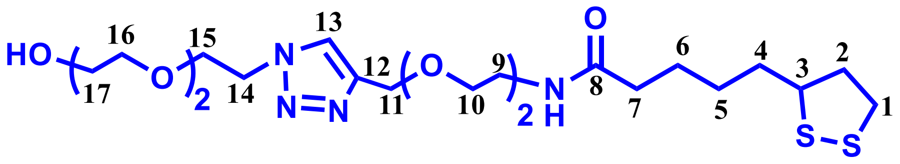

^{1}H NMR (D_2O, 500 MHz): δ = 8.01 (s, 1H, H13), 4.62 (s, 2H, H11), 4.65 (t, 2H, *J*=5.0 Hz, H14), 4.01 (t, 2H, *J*=5.1 Hz, H15), 3.76 – 3.60 (m, 13H, PEG repeats of H10,16,17), 3.55 (t, 2H, *J*=5.3 Hz, H9), 3.35-3.40 (m, 3H, H3,H9), 3.15 – 3.25 (m, 2H, H1), 2.49 (dq, 1H, *J*=12.3, 6.1 Hz, H2), 2.24 (t, 2H, *J*=7.2 Hz, H7), 1.97 (dq, 1H, *J*=13.6, 6.8 Hz, H2), 1.57-1.75 (m, 4H, H4,H6), 1.40 (m, 2H, H5) ppm. ^{13}C NMR (D_2O, 125 MHz): δ = 176.9 (C=O, C8), 143.9 (C12), 125.5 (C13), 71.7 (C11), 69.7 (C14), 69.4, 69.3, 69.0, 68.9, 68.7, 63.1, 60.3 (PEG repeat Cs), 56.5 (C17), 50.0 (C9), 40.2 (C3), 38.9 (C1), 38.0 (C7), 35.4 (C2), 33.7 (C4), 27.7 (C6), 25.0 (C5) ppm. LC-MS: calculated *m/z* for $C_{21}H_{39}N_4O_6S_2$ $(M+H)^+$ 507.23; found 507.04.

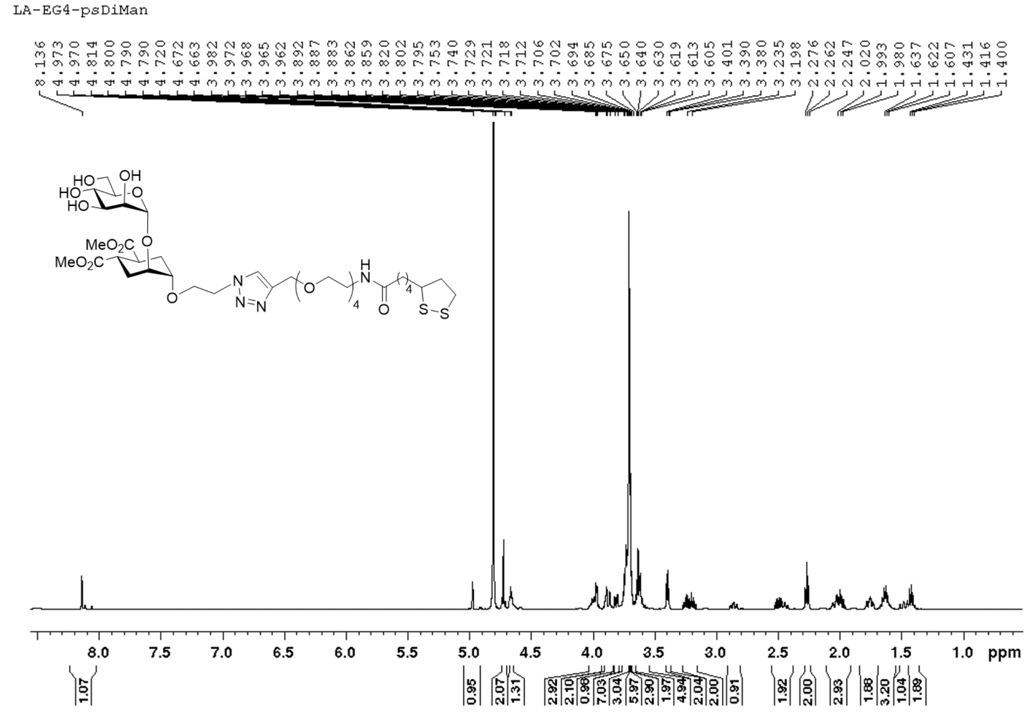

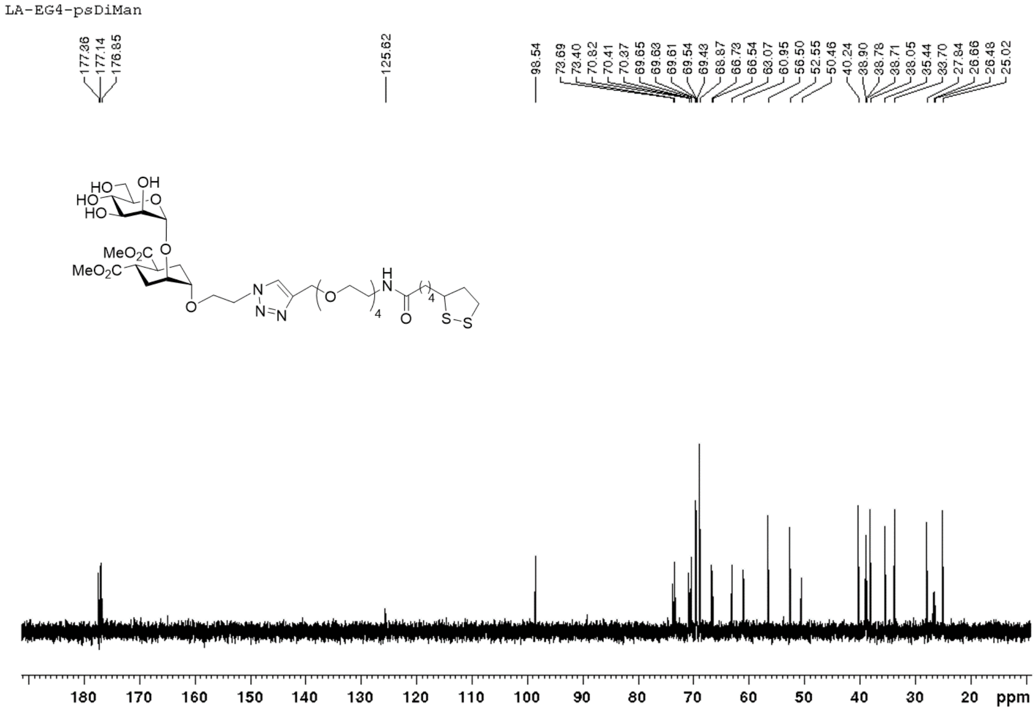

Figure S1. ^{1}H (top panel) and ^{13}C (bottom panel) NMR spectra of LA-EG$_4$-psDiMan in D_2O.

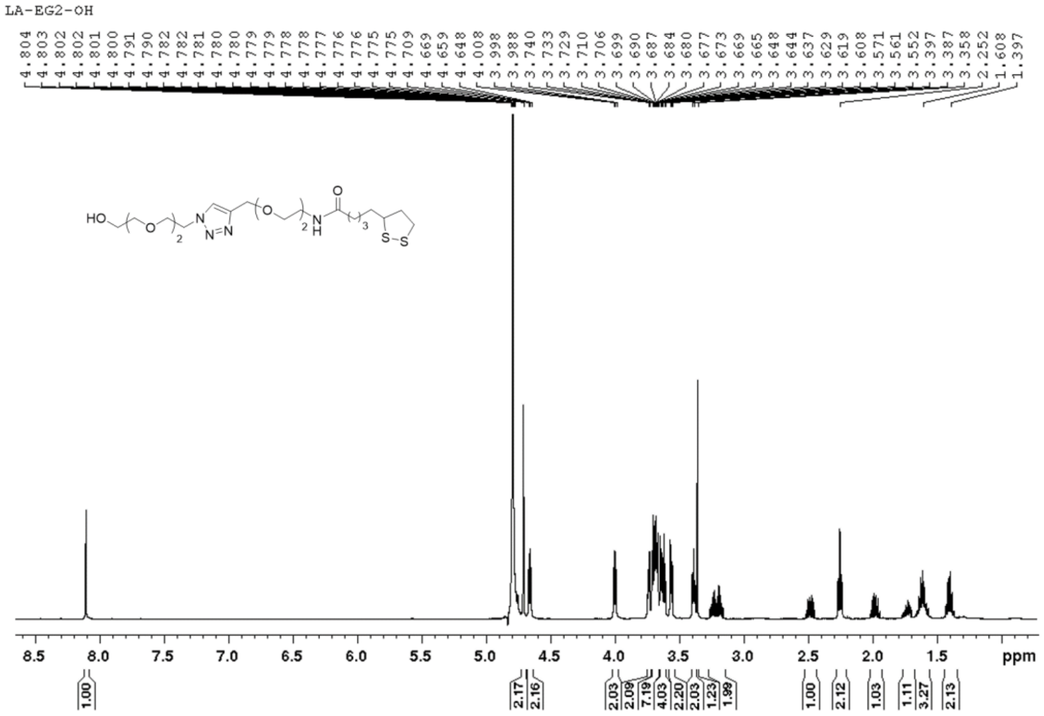

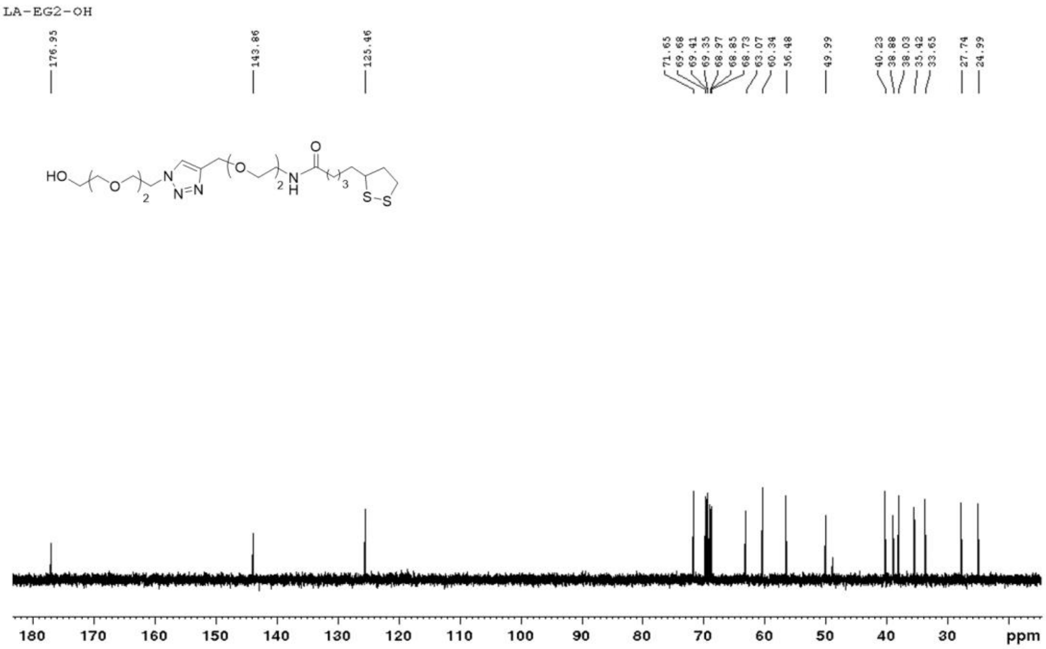

Figure S2. ^{1}H (top panel) and ^{13}C (bottom panel) NMR spectra of LA-EG_2-OH in D_2O.

2.2 Synthesis of gold nanoparticles[3]

The 5 nm gold nanoparticles (G5s) were synthesised in house by following a reported method.[3] The 13 nm gold nanoparticles (G13s) were synthesised by following the standard Turkevich method via citrate reduction of $HAuCl_4$ as reported previously.[4] All glassware used for the synthesis of gold nanoparticles were pre-cleaned with aqua regia, rinsed by deionised water for more than 3 times and dried. The average GNP core sizes were determined from TEM images following statistical analysis of randomly selected particles. The concentrations of G5 and G13 were calculated using the Beer-Lambert law from their UV-vis absorption peak at ~515 and ~520 nm, using the molar extinction coefficients of 6.3 x 10^6 $M^{-1}cm^{-1}$, and 2.32 x 10^8 $M^{-1}cm^{-1}$ for G5 and G13, respectively.[1]

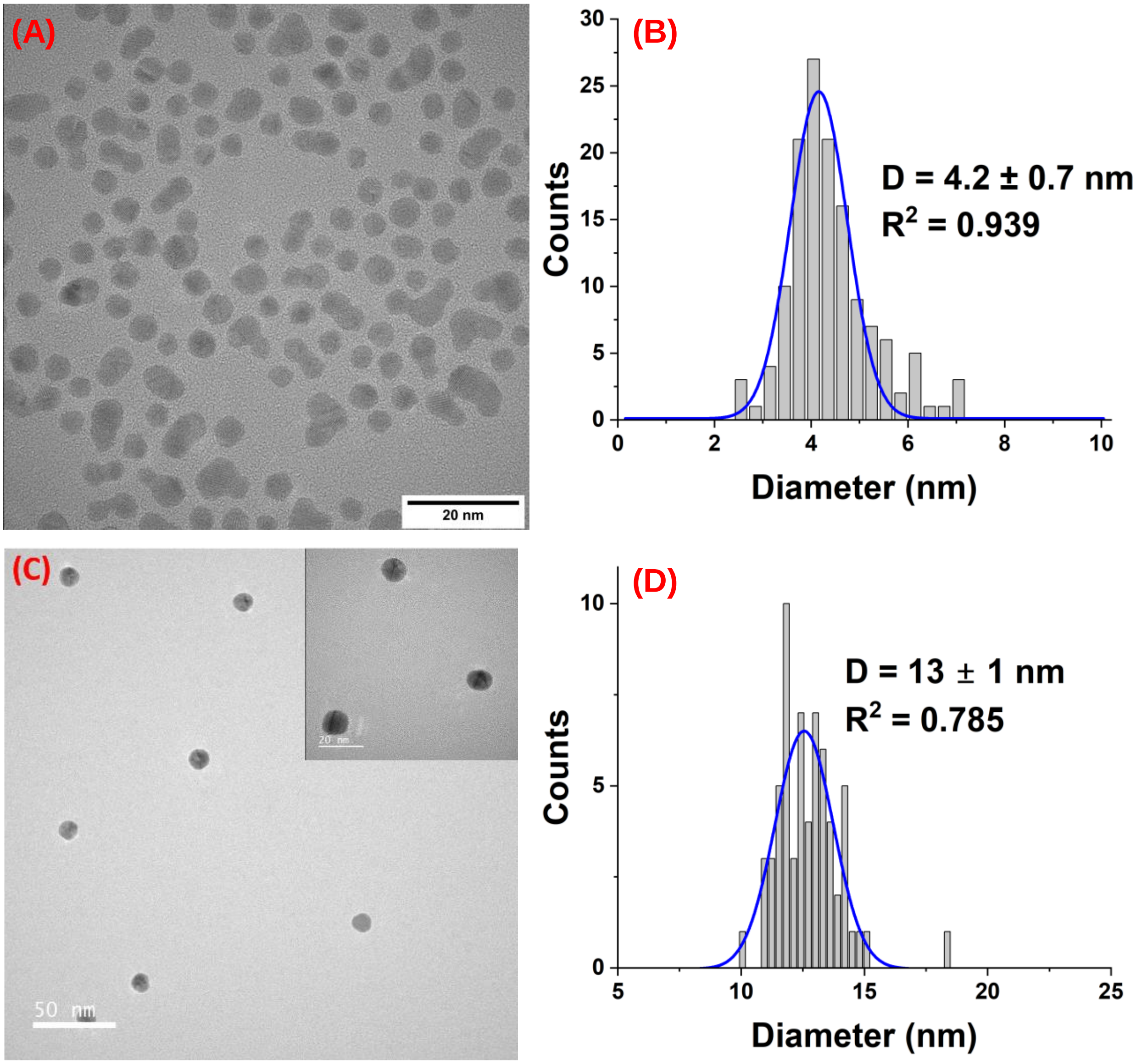

Figure S3. Representative TEM images and their core size distribution histograms with Gaussian distribution fits for nominal 5 nm (G5, **A, B**) and 13 nm (G13, **C, D**) gold nanoparticles, giving an average core diameter of ~4.2 ± 0.7 and 13 ± 1 nm, respectively.

2.3 Preparation and characterisation of Gx-psDiMan conjugates

2.3.1: Estimation of the LA-ligand ratio to fully coat G13

Total gold surface area of one G13, $A_{G13} = 4 \times \pi \times r^2 = 4 \times 3.14 \times (13\ nm/2)^2 = 530\ nm^2$

Assuming each thiol occupies a 3 fold hollow site on the Au(111) surface, the same as that in self-assembled monolayer of alkanethiols on the Au(111) surface,[5] then each thiol occupies a surface area,

$A_{Th} = 0.214\ nm^2$

Each LA ligand forms two Au-S bonds, and thus occupies twice the surface area of an alkanethiol,

$A_{LA} = 2\ A_{Th} = 2 \times 0.214\ nm^2 = 0.428\ nm^2$

The number of LA-based ligand to fully coat each G13:

$N = A_{G13}/A_{TA} = 530/0.428 = \sim1240$

Hence, a total LA-ligand: G13 molar ratio of 3000:1 gives ~2.4 times (e.g., 3000/1240 = ~2.4) the amounts of ligands required to coat the G13 surface by forming a complete self-assembled monolayer.

2.3.2: Procedures for preparing Gx-psDiMan conjugates[1]

For G5-psDiMan conjugates, citrate stabilized G5 was pre-concentrated *via* centrifugation by 4000 rpm, 20 min using a 10 kDa cut-off MWCO filter. The concentrated G5 aqueous solution in an Eppendorf tube was added with the (LA-EG_4-psDiMan + LA-EG_2-EG_2-OH) ligand mixture (total ligand: G5 molar ratio = 1000:1) and incubated at room temperature for 48 h with shaking to make G5-psDiMan conjugates via self-assembly. By systematically varying the LA-EG_4-psDiMan content in the ligand mixture from 100%, 75%, 50%, 25%, 12.5%, 6.3% to 0% (with the rest ligand being LA-EG_2-EG_2-OH), the G5 surface psDiMan density was systematically varied from 100% (capped with 100% LA-EG_4-psDiMan ligand) to 0% (capped with 100% LA-EG_2-EG_2-OH control ligand). Any unbound ligands were then removed by washing G5-psDiMan conjugates with deionised water *via* centrifugation by 10 k ×g, 5 min for 3 times using the 10 kDa cut-off MWCO filter.

For G13-psDiMan conjugates, citrate stabilized G13 was mixed with the (LA-EG_4-psDiMan + LA-EG_2-EG_2-OH) ligand mixture (total ligand: G13 molar ratio = 3000: 1) in a glass vial and the mixture was sonicated for 2 min, then incubated for a further 48 hrs at toom temperature to make G13-psDiMan conjugates via self-assembly. By systematically varying the LA-EG_4-psDiMan content in the ligand mixture from 100%, 75%, 50%, 25%, 12.5%, 6.3% to 0% (with the rest ligand being LA-EG_2-EG_2-OH), the G13 surface psDiMan density was systematically varied from 100% (capped with 100% LA-EG_4-psDiMan ligand) to 0% (capped with 100% LA-EG_2-EG_2-OH control ligand). After incubation, the resulting G13-psDiMan conjugates (with varying psDiMan% content) were transferred to 0.2% Tween 20 pre-treated Eppendorf tubes, then washed with deionised water *via* centrifugation by 17k ×g, 30 min 3 times. The UV-vis absorption spectra of the prepared Gx-psDiMan conjugates were recorded and shown in **Fig. S4A** and their hydrodynamic diameters (D_hs) were characterized by DLS and shown in **Fig. S4B** (for G5-conjugates) and **Fig. S4C** (for G13-conjugates), respectively.

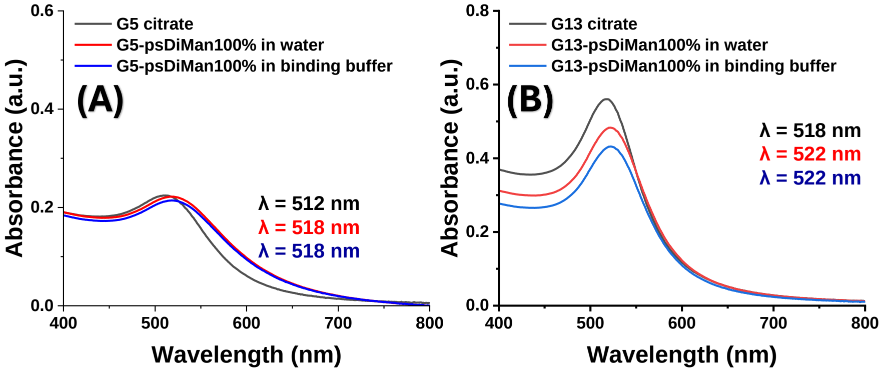

Fig. S4A. UV-vis absorption spectra of (**A**) G5-citrate (dark grey), G5-psDiMan in pure water (red) and in binding buffer (blue); (**B**) G13-citrate (dark grey), G13-psDiMan in pure water (red) and in binding buffer (blue).

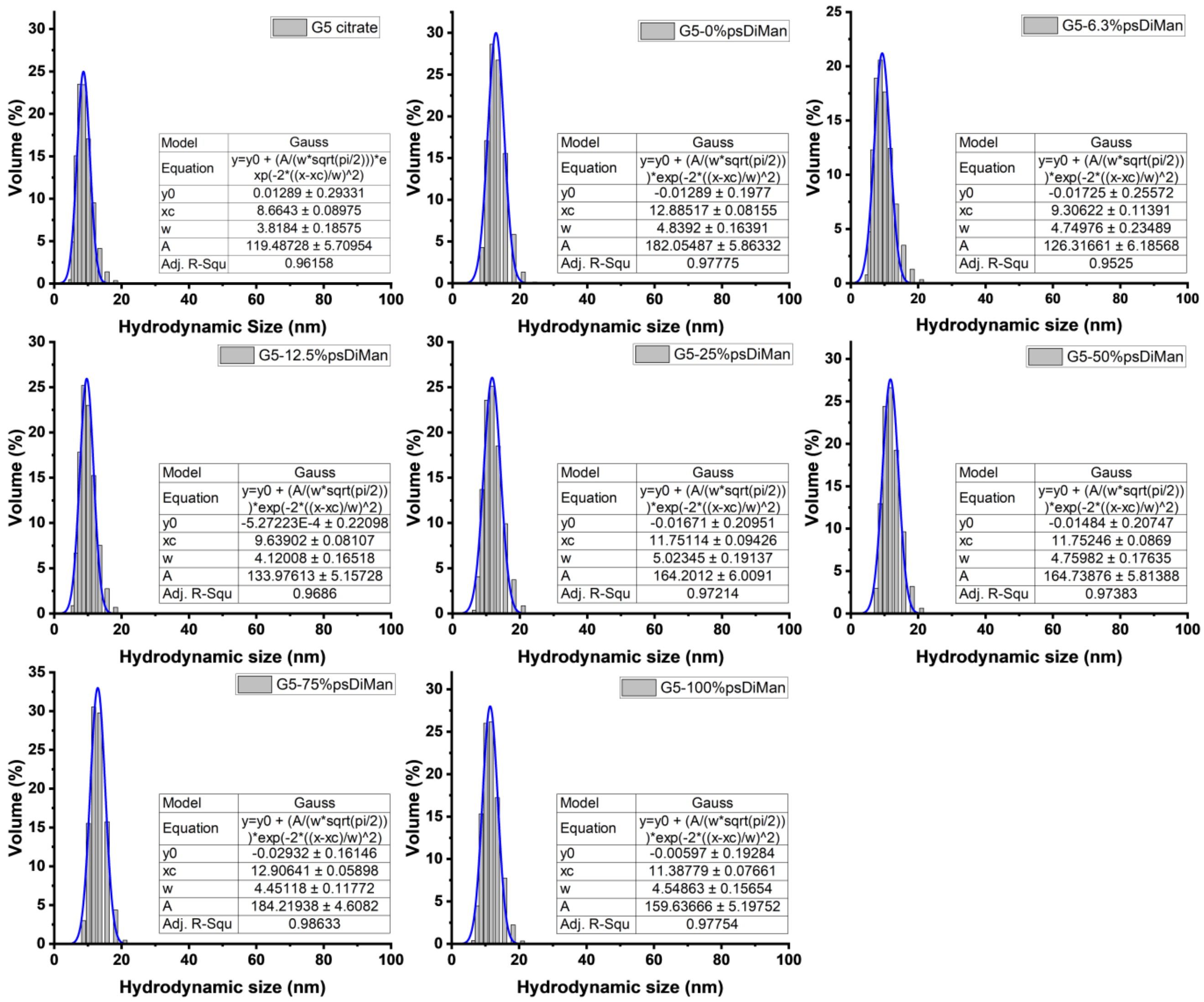

Figure S4B. D_h (volume) distribution histograms with Gaussian distribution fittings of 5 nm GNPs (G5) coated with citrate and varying psDiMan densities, ranging from 0% (coated with 100% LA-EG_2-EG_2-OH spacer ligand), 6.3%, 12.5%, 25%, 50%, 75% and 100% (coated with 100% LA-EG_4-psDiMan ligand).

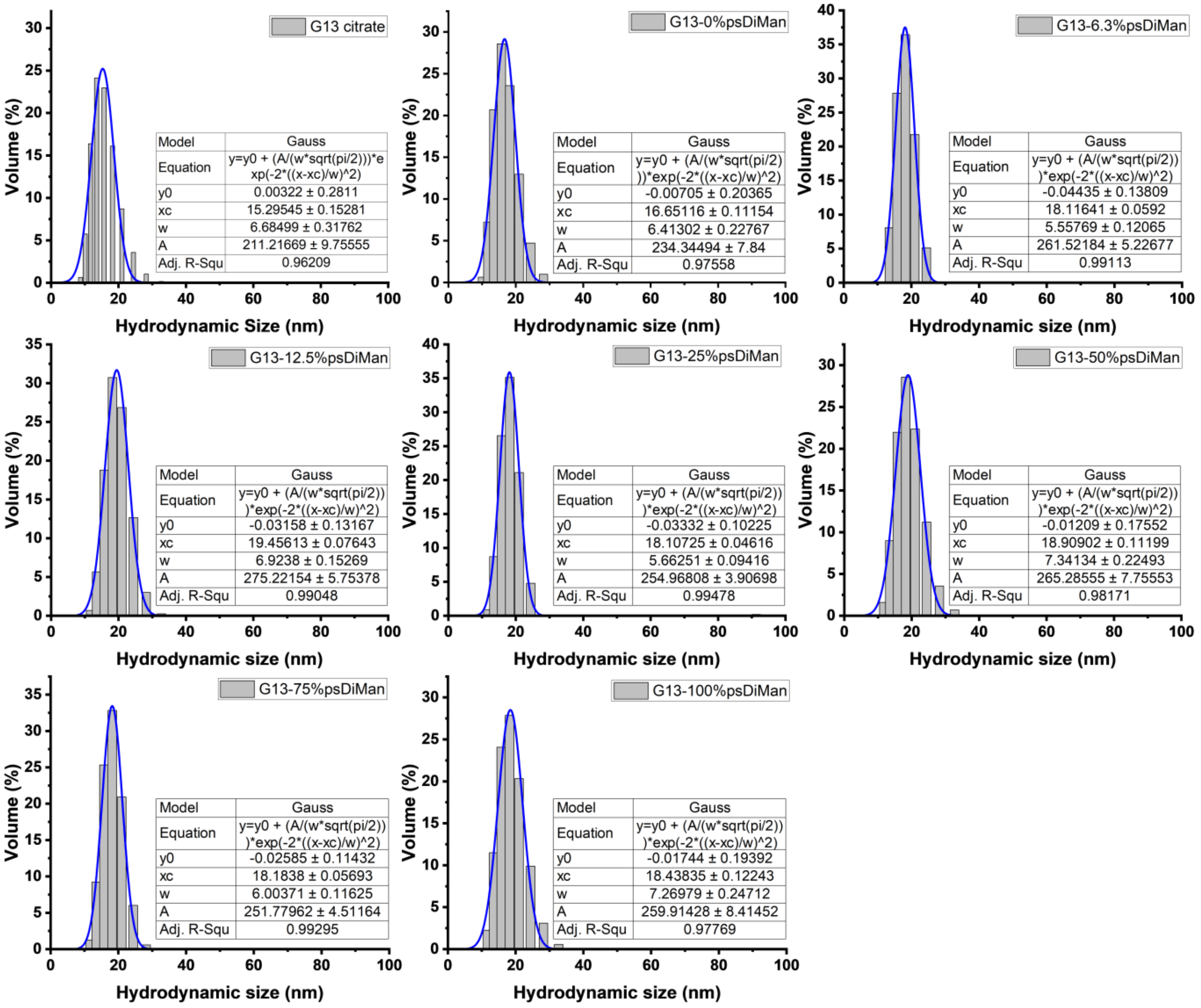

Figure S4C. D_h (volume) distribution histograms with Gaussian distribution fits of 13 nm GNPs coated with citrate and varying psDiMan densities, ranging from 0% (coated with 100% LA-EG_2-EG_2-OH spacer ligand), 6.3%, 12.5%, 25%, 50%, 75% and 100% (coated with 100% LA-EG_4-psDiMan ligand).

2.4 Long-term stability of Gx-psDiMan

All prepared Gx-psDiMan were stored in the refrigerator. To determine the stability of Gx-psDiMan over a long time period, the Gx-psDiMan100% that had been stored over 2 years were checked again by UV-vis spectrometry and DLS. For both G5-psDiMan100% and G13-psDiMan100%, there were no changes on their UV-vis absorption spectra after 2 years storage comparing to the spectra obtained when they freshly prepared, as shown in **Fig.**

S5A. The D_h distribution histograms in **Fig. S5B** indicate that there were no significant aggregations for either G5-psDiMan100% or G13-psDiMan100% over a period of 2 years.

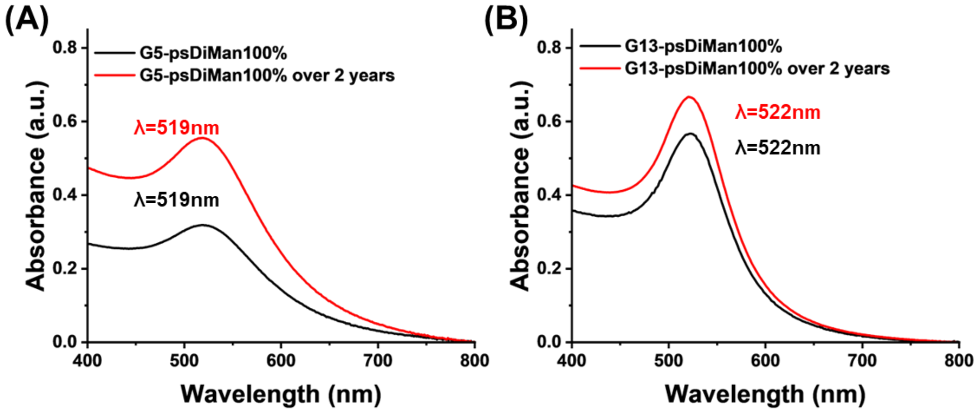

Fig. S5A. UV-vis absorption spectra of (**A**) G5-psDiMan100% (black line, after preparation) and G5-psDiMan100% stored over 2 years (red line); (**B**) G13-psDiMan100% (black line, after preparation) and G13-psDiMan stored over 2 years (red line).

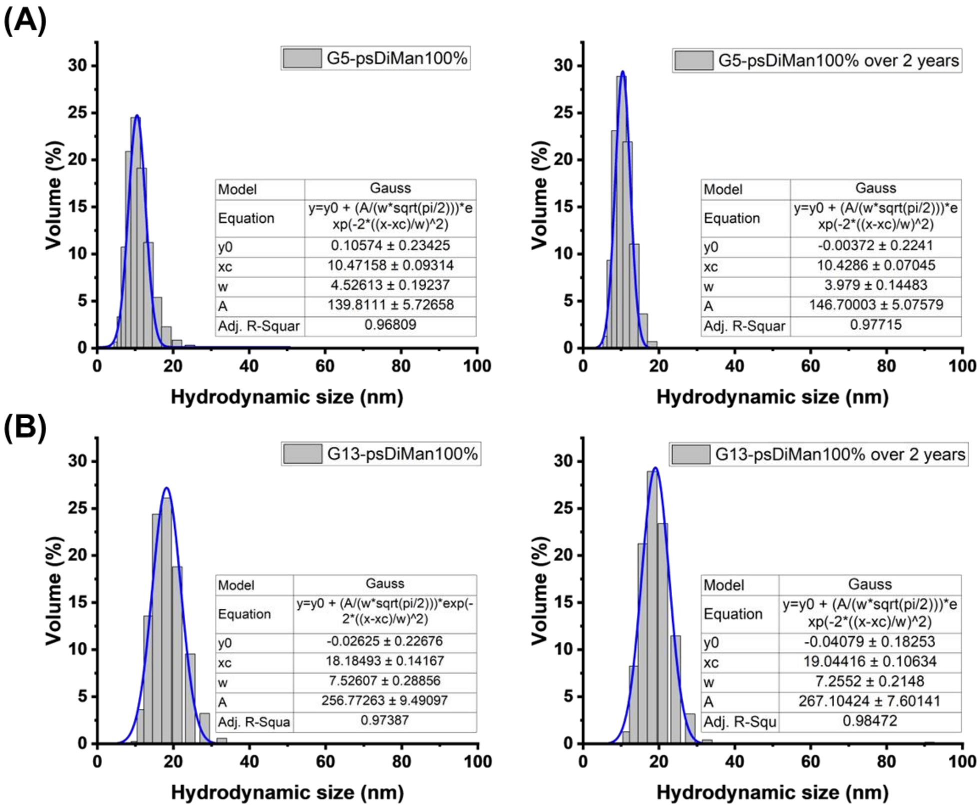

Figure S5B. D_h (volume) distribution histograms with Gaussian distribution fittings of (**A**) G5-psDiMan100% (left) and G5-psDiMan100% stored over 2 years (right); (**B**) G13-psDiMan100% (left) and G13-psDiMan stored over 2 years (right).

3. Determination of glycan amount and inter-glycan distance on GNPs[1,6]

A) The amount of attached glycans on G5 or G13 was determined by performing phenol-sulphuric acid assay. The calibration curve was obtained by mixing different amounts of LA-EG$_4$-psDiMan in 80 µL water, with 80 µL 5% (w/w) phenol and 400 µL H_2SO_4. The reaction was performed in duplicate. After incubation for 30 min, their UV-vis absorbance at 490 nm (A_{490}) was recorded. A baseline correction was performed by adding 80 µL of water with phenol and H_2SO_4 and incubated under the same conditions.

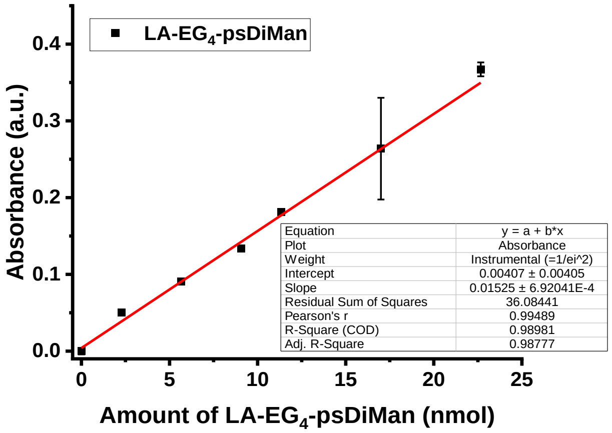

Figure S6. The standard calibration plot for the absorbance *v.s.* LA-EG$_4$-psDiMan amount with linear fit. The fitting parameters are, a = 0.004 ± 0.004; b = 0.0153 ± 0.0007; R^2 = 0.988. The large error for the 16 nmole data point is likely due to pipetting error.

In order to determine the attached glycans amount on GNPs, the unbound LA-EG$_4$-DiMan ligands collected from the supernatant generated from the GNP-psDiMan100% preparation were freeze-dried, and redissolve in 1 mL deionised water. 20 µL of this solution was diluted to 80 µL with deionised water, and then added with 80 µL 5% (w/w) phenol and 400 µL H_2SO_4 and the incubated as above. Each sample was conducted in duplicate. Their UV-vis absorbances at 490 nm were recorded and combined with the calibration curve above to determine the amount of unbound glycan-ligand. The amount of GNP bound glycan ligand was obtained by subtracting the unbound amount from the overall ligand amount added in GNP-psDiMan conjugation. The psDiMan valency per GNP was calculated by dividing the bound ligand amount with the GNP amount.

B) Calculation of inter-glycan distance (X).

The inter-glycan distance (X) on GNPs was estimated from the hydrodynamic size of GNP-conjugates (SI, Fig. S4) and the glycan valency per GNP determined using the method above by following the method first reported by the Mirkin group.[7] Assuming no difference in the GNP anchoring properties between the LA-EG$_4$-psDiMan and LA-EG$_2$-EG$_2$-OH spacer ligand, then the glycan ligand content (%) coated on the GNP should be the same as the LA-EG$_4$-psDiMan ligand content% used in Gx-psDiMan conjugation. Thus, parameters for describing glycan presenting on GNP surface include (1) the surface area occupied by each glycan (glycan footprint k, in nm^2), (2) the angle of two nearest glycans (average deflection angle ϑ, in degrees), and (3) the inter-glycan distance (X, in nm) can be calculated by the following equations:[1, 7]

$$k = \frac{\pi D_h^2}{N} \qquad (S1)$$

$$\theta = \frac{360\sqrt{\frac{k}{\pi}}}{\frac{D_h}{2}\pi} \qquad (S2)$$

$$X = D_h \sin\left(\frac{\theta}{2}\right) \qquad (S3)$$

Where D_h is the hydrodynamic diameter of GNP-glycan conjugates, and *N* is the average number of glycans on each GNP.

Table S1. Summary of key parameters of Gx-psDiMan conjugates under different glycan densities. D_h = hydrodynamic diameter (mean ± ½ FWHM); N = glycan valency per GNP (assuming identical binding properties for LA-EG_4-psDiMan and LA-EG_2-EG_2-OH ligand); k = footprint of per glycan, Θ = average glycan deflection angle, and X = average inter-glycan distance

Gx	psDiMan content (%)	D_h (nm)	psDiMan valency (N)	k (nm^2)	Θ (°)	X (nm)
G5	0	12.9 ± 2.4	0	-	-	-
	6.3	9.3 ± 2.4	30 ± 3	9.1 ± 0.8	41.8 ± 1.9	3.4 ± 0.2
	12.5	9.6 ± 2.1	60 ± 5	4.9 ± 0.4	29.7 ± 1.3	2.5 ± 0.1
	25	11.8 ± 2.5	119 ± 11	3.7 ± 0.3	21.0 ± 1.0	2.2 ± 0.1
	50	11.8 ± 2.4	238 ± 22	1.8 ± 0.2	14.9 ± 0.7	1.53 ± 0.07
	75	12.9 ± 2.2	357 ± 32	1.5 ± 0.1	12.1 ± 0.6	1.37 ± 0.06
	100	11.4 ± 2.3	476 ± 43	0.9 ± 0.1	10.5 ± 0.5	1.05 ± 0.05
G13	0	16.7 ± 3.2	0	-	-	-
	6.3	18.1 ± 2.8	124 ± 12	8.3 ± 0.8	20.6 ± 1.0	3.3 ± 0.2
	12.5	19.5 ± 3.3	245 ± 23	4.9 ± 0.5	14.6 ± 0.7	2.5 ± 0.1
	25	18.1 ± 2.9	491 ± 47	2.1 ± 0.2	10.4 ± 0.5	1.6 ± 0.1
	50	18.9 ± 3.7	982 ± 93	1.1 ± 0.1	7.3 ± 0.4	1.21 ± 0.06
	75	18.2 ± 3.0	1472 ± 140	0.71 ± 0.07	6.0 ± 0.3	0.95 ± 0.04
	100	18.4 ± 3.6	1963 ± 186	0.54 ± 0.05	5.2 ± 0.3	0.83 ± 0.04

4. Production and labeling of DC-SIGN and DC-SIGNR[1, 6, 8]

4.1. The wild-type (WT) DC-SIGN, DC-SIGNR and Atto-643 labelled DC-SIGN and DC-SIGNR were produced by our established protocols as published previously.[4] The concentration of WT DC-SIGN and DC-SIGNR was determined by recoding its UV-vis absorption at 280 nm (A_{280}) on a Nanodrop instrument with an optical path length of 1 mm using the Beer-Lambert law. The extinction coefficients used protein concentration calculation were 2.82 x 10^5 and 2.44 x 10^5 M^{-1} cm^{-1} for WT DC-SIGN and DC-SIGNR tetramers, respectively. For Atto-643 labelled DC-SIGN and DC-SIGNR, their concentrations were determined by recording their UV absorption at 280 nm (A_{280}) and 643 nm (A_{643}), then calculated using equations S4 and S5, respectively:

$$C = \frac{A_{280} - A_{643} \times 0.04}{282000} \tag{S4}$$

$$C = \frac{A_{280} - A_{643} \times 0.04}{244000} \tag{S5}$$

Where 0.04 is a correction factor for Atto-643 absorbance at 280 nm.

4.2. Confirmation of protein identity and Atto-643 labeling by HRMS[2]

WT DC-SIGN: calculated molecular weight based on its amino acid sequence with no disulphide bond formation: 39197.22, found 39201.38. Atto-643 labelled DC-SIGN-Q274C: calculated molecular weight based on no disulphide bond formation: 40130.22, found 40136.45.

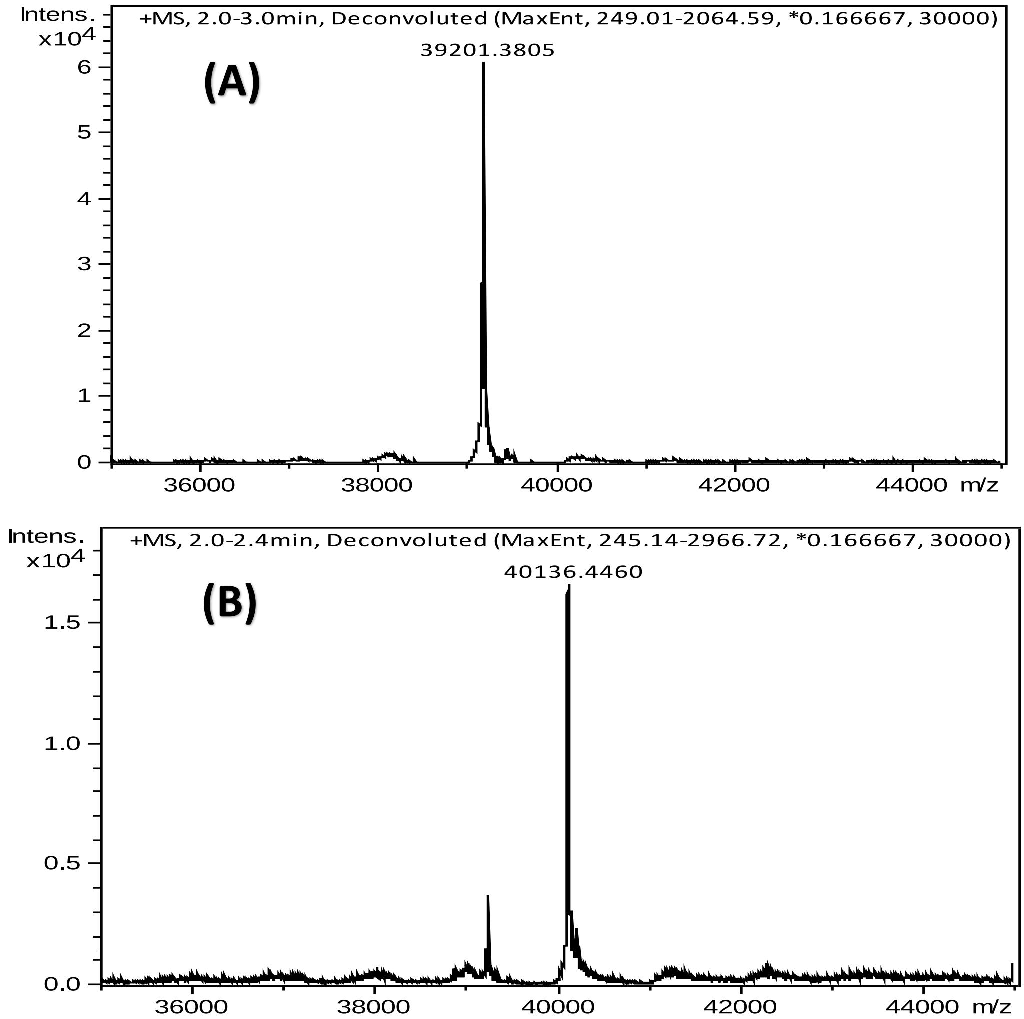

Figure S7. HRMS spectrum of (**A**) wild-type DC-SIGN and (**B**) Atto-643 labelled DC-SIGNQ274C. Labeling efficiency was calculated as 92.3% using the molecular mass peak area of the unlabeled and labeled protein.

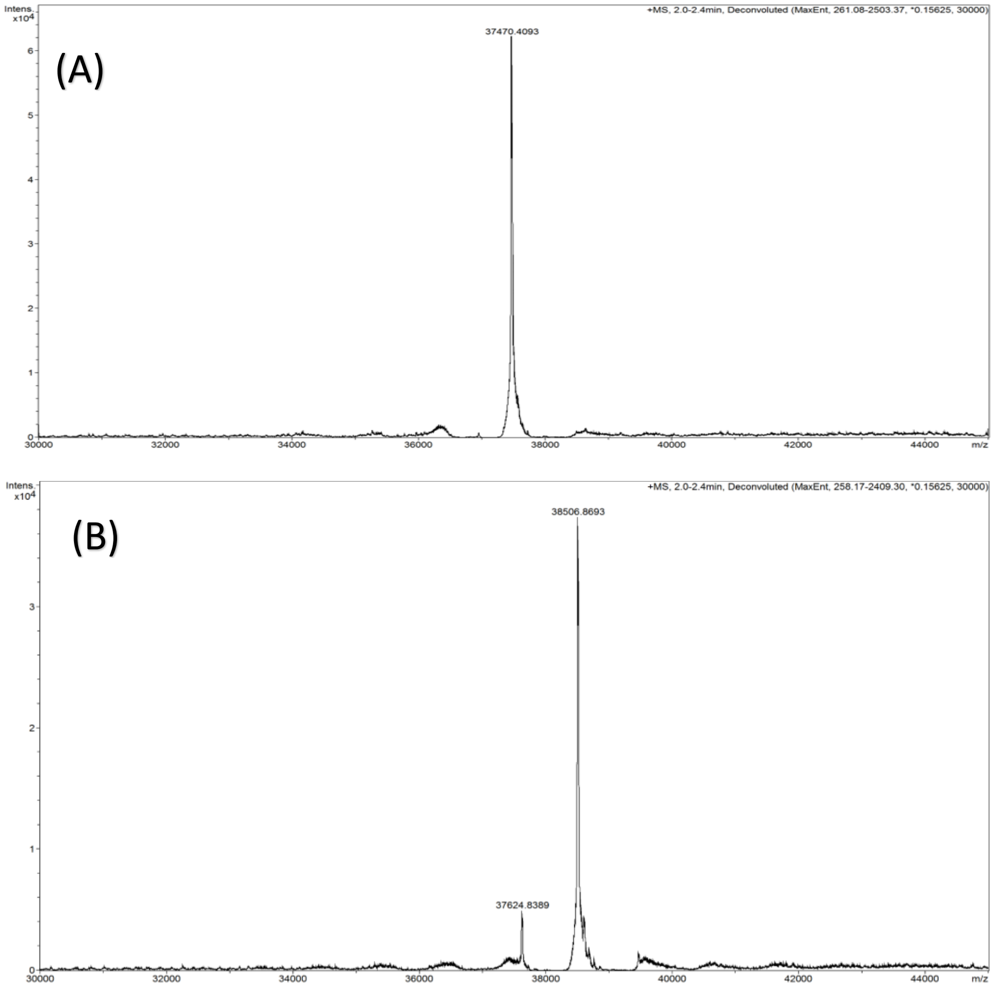

Figure S8. HR-MS spectra of (**A**) wild-type DC-SIGNR and (**B**) Atto-643 labelled DC-SIGNR-R287C. Using the peak areas of the unlabeled and labeled peaks, Atto-643 labeling efficiency per protein monomer was estimated as ~90%.

5. GNP Fluorescence quenching assay [1]

5.1. MLGI Binding Affinity

DC-SIGN and G5-psDiMan (with psDiMan density of 0%, 6.3%, 12.5%, 25%, 50%, 75% and 100%) were mixed at a fixed molar ratio of 1: 1, with varying concentrations of their mixtures of 0.1, 0.2, 0.5, 1, 2, 4, 8 and 16 nM in binding buffer (20 mM HEPES, 100 mM NaCl, 2 mM $CaCl_2$, pH 7.8) containing 1 mg/mL BSA, respectively. DC-SIGN and G13-psDiMan (with psDiMan density of 0%, 6.3%, 12.5%, 25%, 50%, 75% and 100%) were mixed at a fixed molar ratio of 1: 1, with varying concentrations of their mixtures of 0.1, 0.2, 0.4, 0.8, 1.6, 3.2 and 6.4 nM in the binding buffer, respectively. The samples were incubated for 20 min at room temperature, then transferred to a quartz cuvette and recorded their fluorescence spectra immediately, with the excitation at 630 nm and emission recorded from 650 to 800 nm.

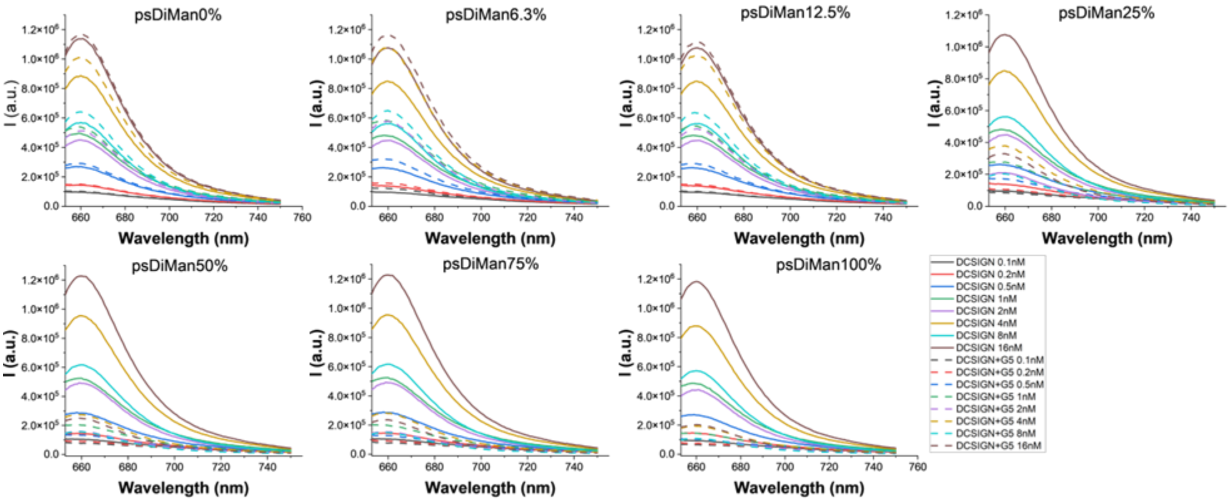

Figure S9. Fluorescence spectra of 1:1 molar mixed G5-psDiMan (with varying psDiMan contents of 0%, 6.3%, 12.5%, 25%, 50%, 75% and 100%) and Atto-643 labeled DC-SIGN at a variety of concentrations ranging from 0.1 to 16 nM.

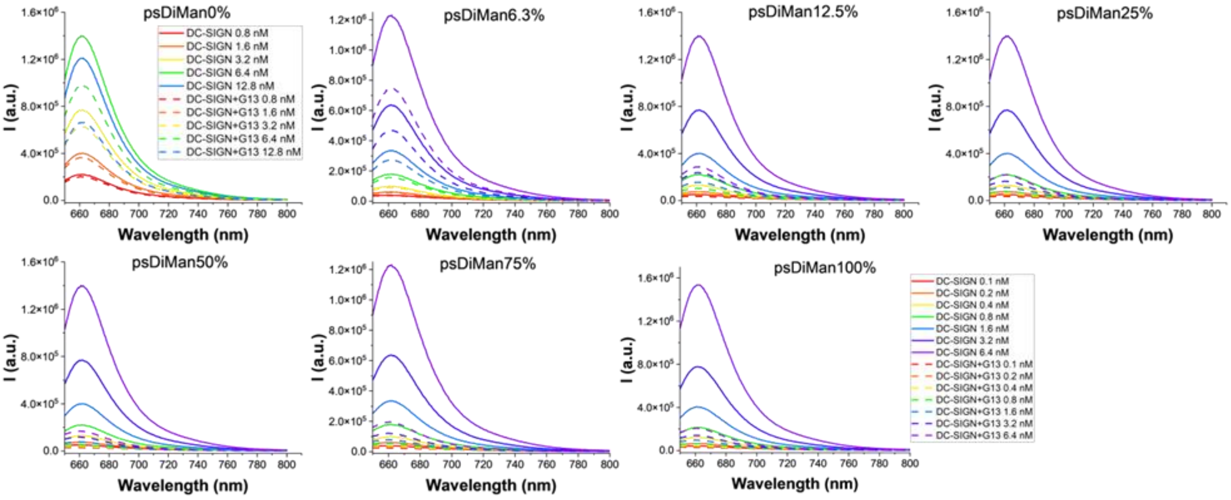

Figure S10. Fluorescence spectra of 1:1 molar mixed G13-psDiMan (with varying psDiMan contents of 0%, 6.3%, 12.5%, 25%, 50%, 75% and 100%) and Atto-643 labelled DC-SIGN at a variety of concentrations (from 0.1 to 6.4 nM).

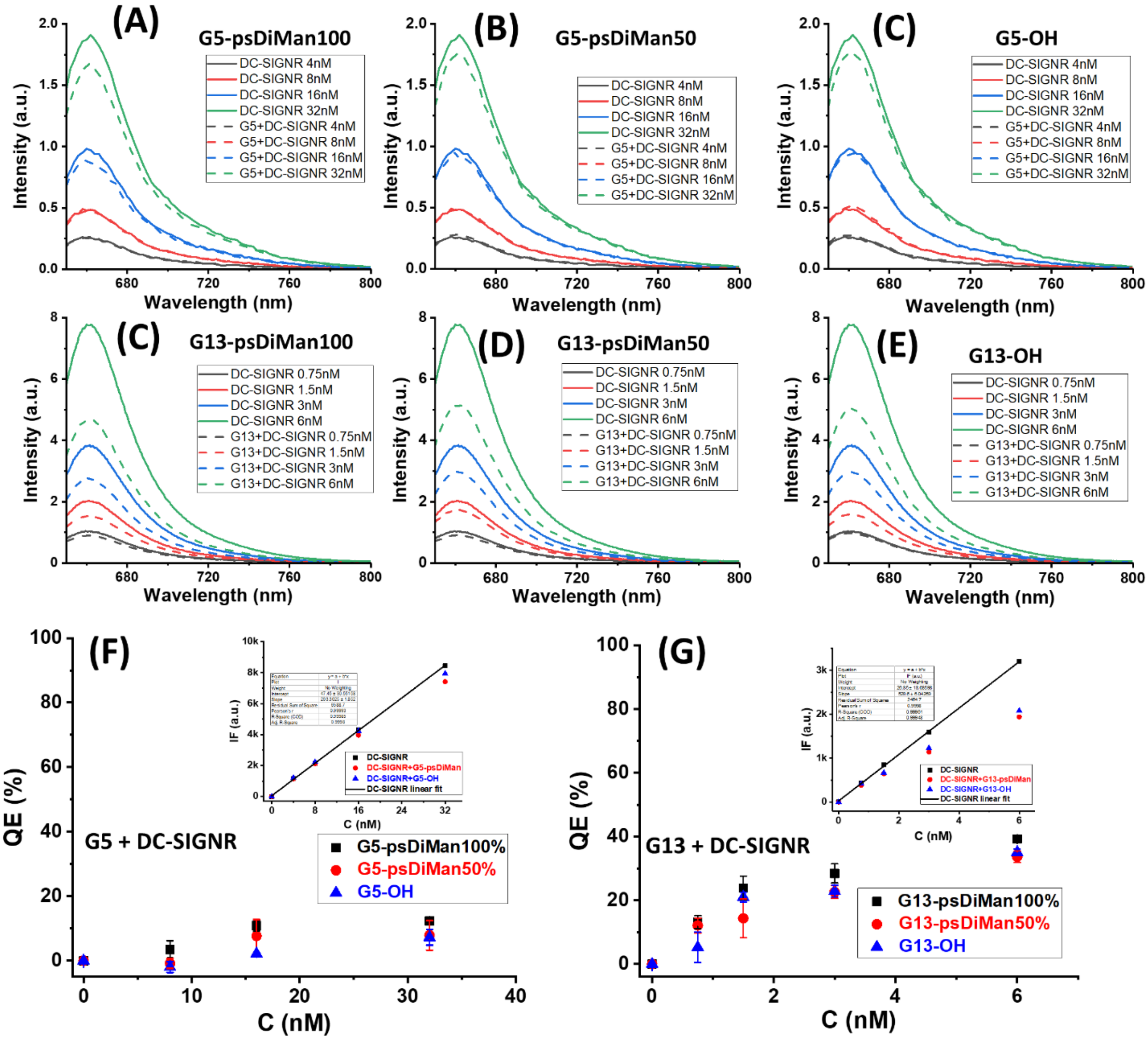

Figure S11. Fluorescence spectra of varying concentrations of Atto-643 labeled DC-SIGNR (4 to 32 nM for G5; 0.75 to 6 nM for G13) after mixing with 1 molar equivalent of G5-psDiMan100% (**A**), G5-psDiMan50% (**B**), G5-OH control (**C**), G13-psDiMan100% (**D**), G13-psDiMan50% (**E**) or G13-OH control (**F**) in a binding buffer. Please note that the fluorescence spectra for G5 and G13 sample series were collected under different instrument sensitivities, hence have different absolute fluorescence signals, although this had no effect on their QEs. Comparison of QE *vs.* concentration plot for DC-SIGNR after mixing with 1 molar equivalent of G5-conjugates (**F**) or G13-conjugates (**G**) with insets showing their integrated fluorescence *vs.* concentration relationships.

In the absence of Gx-conjugates, DC-SIGNR fluorescence increases linearly with concentration in both cases, while the presence of Gx-psDiMan only quenched DC-SIGNR fluorescence at relatively high concentrations (e.g., 32 nM for G5 or ≥ 1.5 nM for G13). Due to the far greater plasmon absorption extinction coefficient of G13 over that of G5 (e.g., 2.3×10^8 *vs.* 1.1×10^7 $M^{-1}cm^{-1}$), G13's inner filter effect is >20 times stronger than that of G5. This result is consistent with ~20-fold concentration difference in obtaining observable QEs between G5- and G13- conjugates. Importantly, the QEs observed with the Gx-OH controls and Gx-psDiMan conjugates are very similar across the concentration range studied; suggesting that the observed quenching here are non-specific and mainly due to Gx's inner filter effect.

5.2. MLGI Binding Thermodynamics

For thermodynamics study, DC-SIGN and G5-psDiMan were mixed at a fixed molar ratio of 1: 1, with varying concentrations of their mixtures of 0.1, 0.2, 0.5, 1, 2, 4, 8, 16, 32 and 64 nM in binding buffer, respectively. DC-SIGN and G13-psDiMan were mixed at a fixed molar ratio of 1: 1, with varying concentrations of their mixtures of 0.1, 0.2, 0.4, 0.8, 1.6, 3.2 and 6.4 nM in binding buffer, respectively. Once the samples were prepared, they were incubated on a dry bath or a water bath to maintain the specified temperature for samples for 20 min (G5-psDiMan at 20°C, 25°C and 30°C and G13-psDiMan at 25°C, 30°C and 35°C). Thus, the temperature related binding K_Ds were then obtained from Hill's equation fitting and plotted by Van 't Hoff equation as well as Gibbs equations to analyse the thermodynamic parameters.

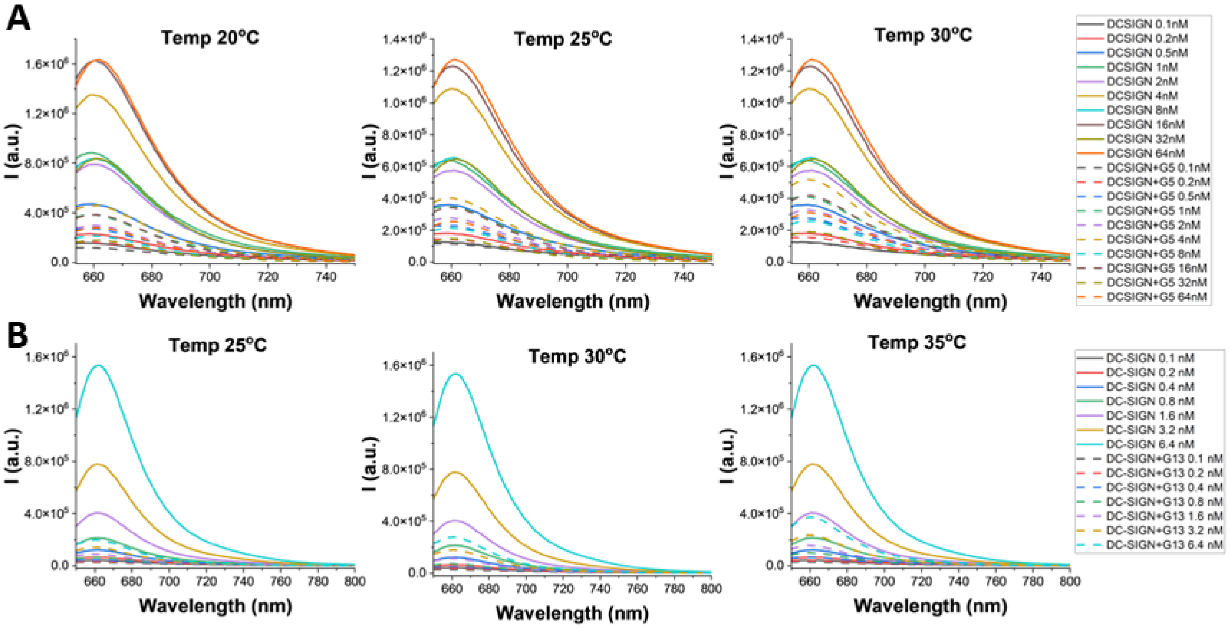

Figure S12. Fluorescence spectra of 1:1 mixed (**A**) G5-psDiMan and Atto-643 labelled DC-SIGN at 20°C, 25°C and 30 °C and (**B**) G13-psDiMan and Atto-643 labelled DC-SIGN at 25°C, 30°C and 35°C at varying concentrations.

6. Isothermal Titration Calorimetry (ITC)[9]

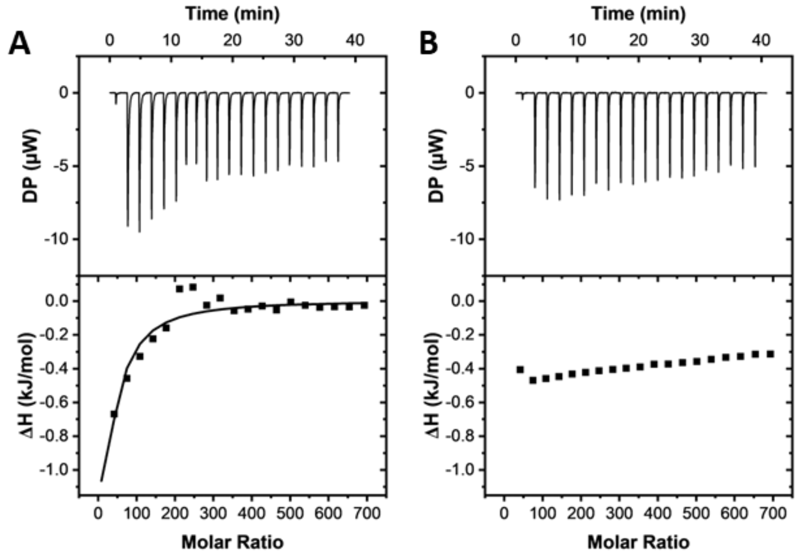

Figure S13. ITC titration curves of monovalent psDiMan (50 mM) titration into (**A**) wild type DC-SIGN (15 μM) and (**B**) HEPES dialysis buffer. The following binding thermodynamic parameters were obtained: K_d = 1.1 ± 0.3 mM, ΔH° = -23.4 ± 2.7 kJ mol^{-1}, ΔG° = -17.0 kJ mol^{-1} and ΔS° = -21.5 J K^{-1} mol^{-1}.

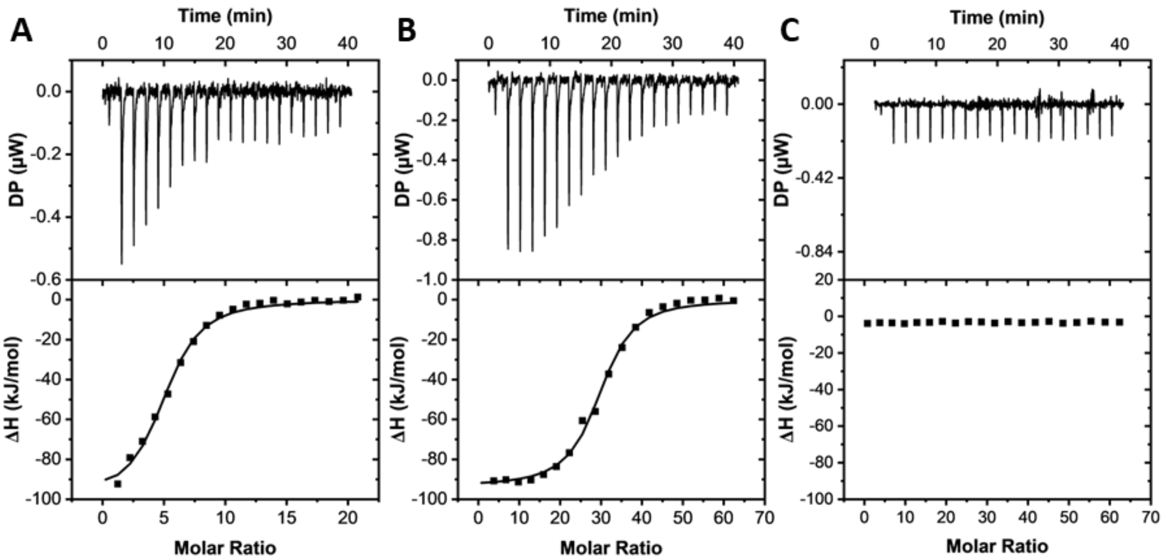

Figure S14. ITC titration curves of DC-SIGN (30 μM) titration into (**A**) G5-psDiMan50% (300 nM), (**B**) G13-psDiMan50% (100 nM), and (**C**) HEPES dialysis buffer. The titration curve was fitted with the same method described above to obtain ΔH° values for G5-psDiMan50% and G13-psDiMan50% of -99.4 ± 2.7 kJ mol^{-1} and -93.6 ± 1.5 kJ mol^{-1}, respectively.

7. DLS studies of Gx-psDiMan 100% interaction with DC-SIGN and DC-SIGNR[1, 8]

To determine the hydrodynamic diameters (*D*hs) of the formed G5-psDiMan100%-DC-SIGN/R complexes, 10 nM G5-psDiMan100% was mixed with varying concentrations of DC-SIGN in a binding buffer (20 mM HEPES, 100 mM NaCl, 10 mM $CaCl_2$, pH 7.8) to obtain a protein: G5 molar ratio (PGR) of 1, 2, 4, 5, 6, 8 and 10, respectively. The samples were incubated at room temperature for 20 minutes. The *D*hs were recorded on a Malvern Zetasizer and the volume distribution histograms obtained from the instrument software were fitted by Gaussian function using OriginPro. The *D*hs of DC-SIGN and G5-psDiMan100% alone in the binding buffer were measured as ~12 and ~13 nm, respectively. Mixing 1 molar equivalent of DC-SIGN with G5-psDiMan gave a large increase of D_h to ~41 nm, indicating the formation of G5-psDiMan100%-DC-SIGN complexes, and further increasing DC-SIGN ratio produced further but slower increase of D_h which eventually plateaued at ~55 nm as a PGR to ~5, indicating DC-SIGN binding saturation on each G5-psDiMan surface. However, the D_h drop down to ~16 nm as PGR was > 8 times to G5, it is due to the excess DC-SIGN that was detected and counted as the majority particles by the instrument, thus causing the decrease to the overall D_h distributions.

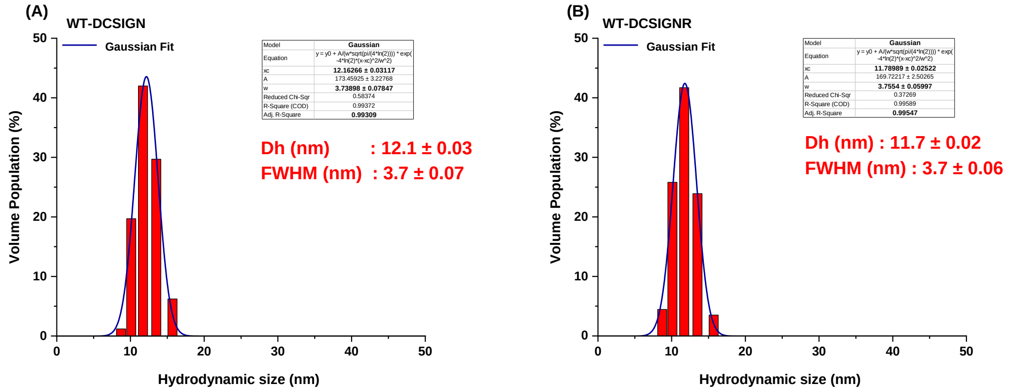

Figure S15. Representative D_h (volume) distribution histograms of (**A**) wild-type DC-SIGN and (**B**) wild-type DC-SIGNR in a binding buffer fitted by Gaussian function, giving a D_h and FWHM of ~ 12 and ~3.7 nm for DC-SIGN, and ~11.7 and ~3.7 nm for DC-SIGNR, respectively.

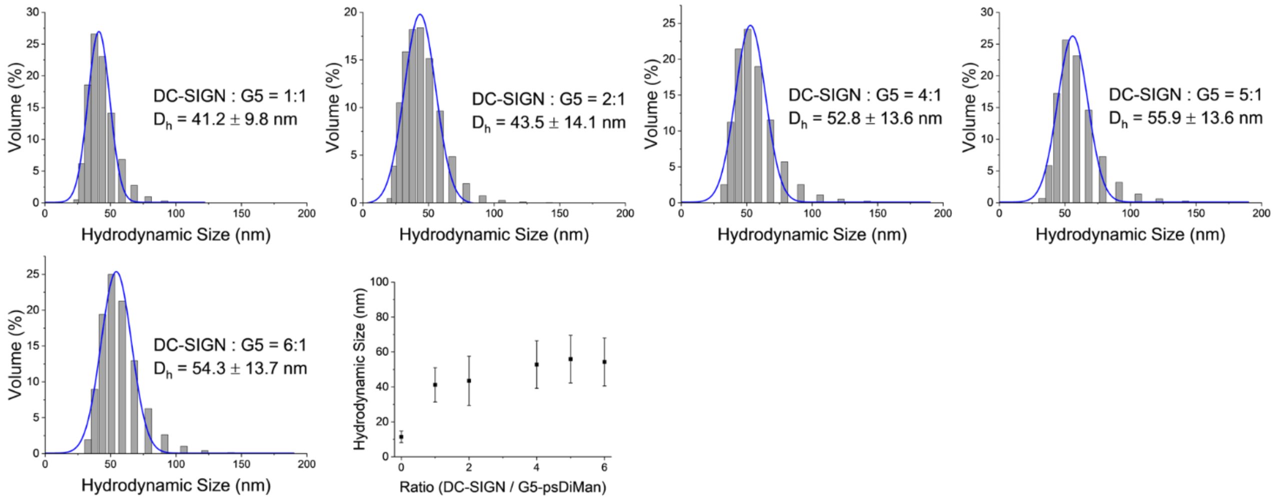

Figure S16. D_h (volume population) distribution histograms of G5-psDiMan100% (10 nM) after mixing wild-type DC-SIGN under a range of DC-SIGN: G5 molar ratios, and the corresponding plot of D_h *vs.* Ratio (WT DC-SIGN / G5-psDiMan100%). (D_h is given as mean ± ½ FWHM, FWHM = full width at half maximum of the Guassian fit). D_h was found to increase with the increasing protein: GNP molar ratio, and plateaued at ~5:1)

To determine the hydrodynamic sizes of the G13-psDiMan100%-DC-SIGN complexes, 4 nM G13-psDiMan100% each was mixed with varying concentrations of wild-type DC-SIGN or DC-SIGNR in a binding buffer (20 mM HEPES, 100 mM NaCl, 10 mM $CaCl_2$, pH 7.8) to give the protein: G13 molar ratios of 4:1, 8:1, 16:1, 32:1, 48:1, and 60:1, respectively. The samples were incubated at room temperature for 20 minutes. The hydrodynamic size distributions were recorded on Malvern Zetasizer and then analyzed on OriginPro by fitting the distribution histograms using the standard Gaussian function (single or multiple peak, depending on their distribution). For some DC-SIGNR binding samples showing two distribution peaks, the histograms were fitted by two Gaussian peaks and the linear average of the two species based on their abundances were used to calculate their average D_h and FWHM values. For example, Mean $D_h = D_{h1} \times [A_1/(A_1 + A_2] + D_{h2} \times [A_2/(A_1 + A_2]$; mean $FWHM = FWHM_1 \times [A_1/(A_1 + A_2] + FWHM_1 \times [A_2/(A_1 + A_2]$, where A_1 and A_2 are the peak area of species 1 and species 2 obtained from Gaussian fitting, respectively.

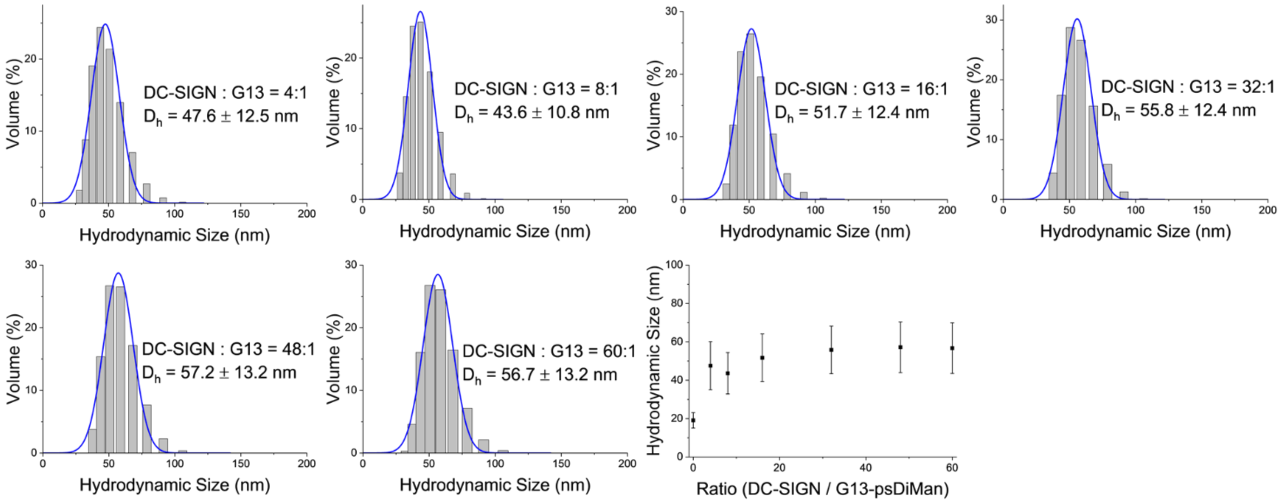

Figure S17. D_h (volume population) distribution histograms of G13-psDiMan100% (4 nM) after mixing with wild-type DC-SIGN under a variety of DC-SIGN: G13 molar ratios, and the plot of D_h vs. Ratio (WT DC-SIGN / G13-psDiMan100%). (D_h is given as mean ± ½ FWHM, FWHM = full width at half maximum of the Gaussian fit). D_h was found to increase with the increasing protein: GNP molar ratio and plateaued at ~32:1.

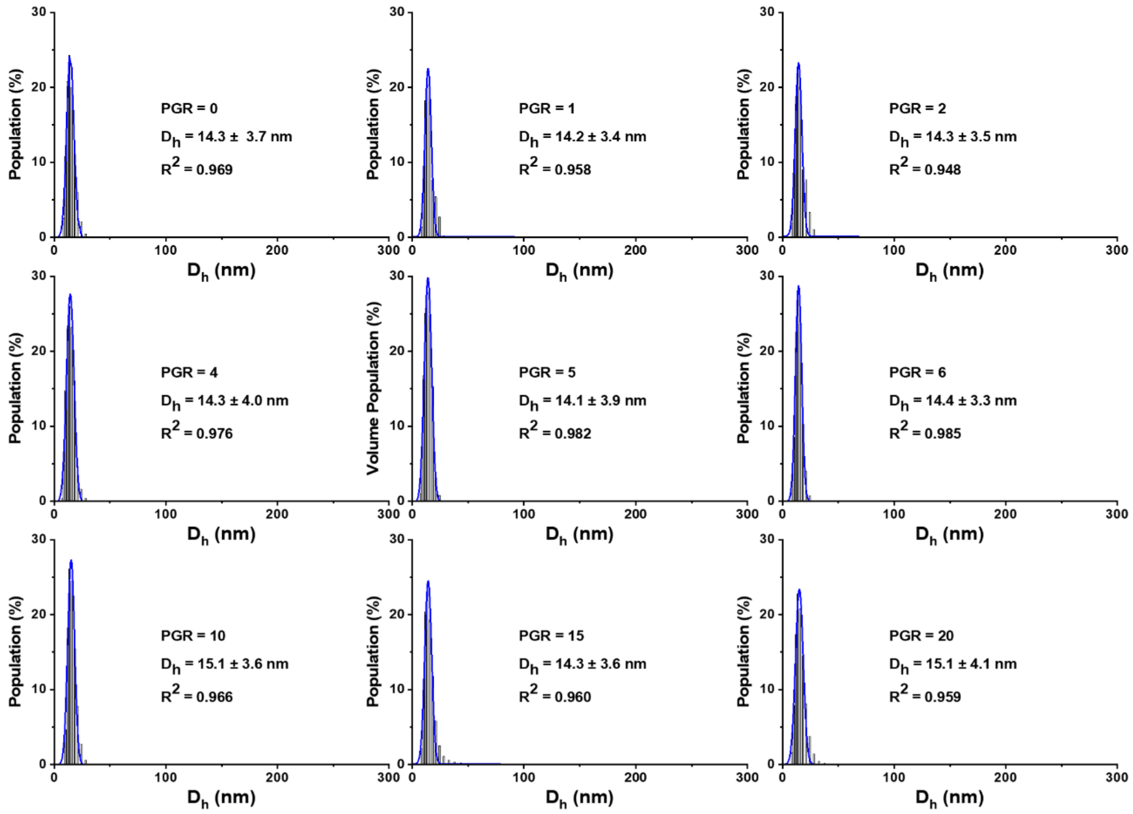

Figure S18. D_h (volume) distribution of G5-psDiMan100% (10 nM) after mixing with varying concentrations of the wild-type DC-SIGNR to give a final protein: G5 molar ratio (PGR) over a range of 0 to 20. All samples effectively displayed the same D_h size (14-15 nm) as the G5-psDiMan100% alone (~14 nm), suggesting no apparent binding throughout the concentration range studied.

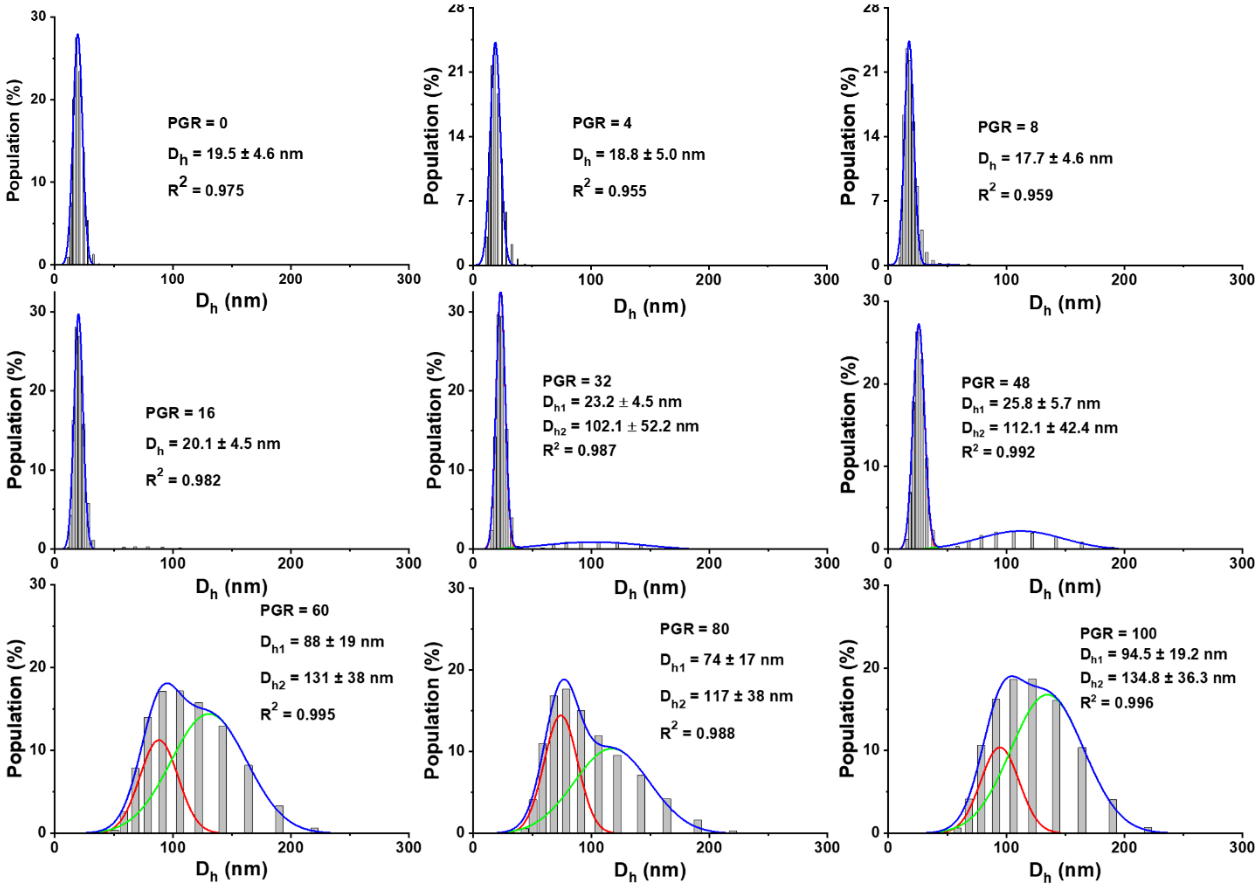

Figure S19. D_h (volume) distribution of G13-psDiMan100% (4 nM) after mixing with varying concentrations of wild-type DC-SIGNR to give a protein: G13 molar ratio (PGR) over a range of 0 to 100. Samples at low PGRs (≤16) effectively have the same D_h as G13-psDiMan100% alone; those at intermediate PGRs (e.g., 32 and 48) display two distinct species with D_hs of ~25 and ~110 nm, corresponding to isolated G13-psDiMan and clustered DC-SGNR-G13-psDiMan complexes, respectively; while those at the high PGRs (e.g., 60-100) exhibit only large D_hs (~95 and ~135 nm), corresponding to clustered DC-SGNR-G13-psDiMan complexes. These results indicate that DC-SIGNR shows weak crosslinking interactions with G13-psDiMan100%, detectable only at high concentrations.

8. Gx-psDiMan Cytotoxicity Assay

1 × 10^4 HEK 293 cells were seeded to each well in a 96-well plate. After 24 hours, G5-psDiMan100%, G5-psDiMan50%, G5-OH, G13-psDiMan100%, G13-psDiMan50%, and G13-OH were added to wells respectively to final concentrations of 3.1 and 15.5 nM (triplicates for each), corresponding to the highest concentrations of Gx-conjugates used in the viral inhibition assays. The cells without adding any nanoparticles were used as the negative control. After incubation overnight, the medium was removed, cells were washed gently with PBS to remove any unbound nanoparticles. Then 100 uL of 0.5 mg/mL MTT (in phenol red-free medium) were added to each well and incubated for 2.5 hours at 37 °C. After incubation, free MTT and medium were removed, 100 uL DMSO then added to each well to dissolve the formed formazan. After incubated for another 15 mins at 37 °C, the absorbance at 550 nm were read on CLARIOstar plate reader. The absorbance data were normalised to assess the cytotoxicity of the Gx-glycan conjugates as shown in Figure S17 below.

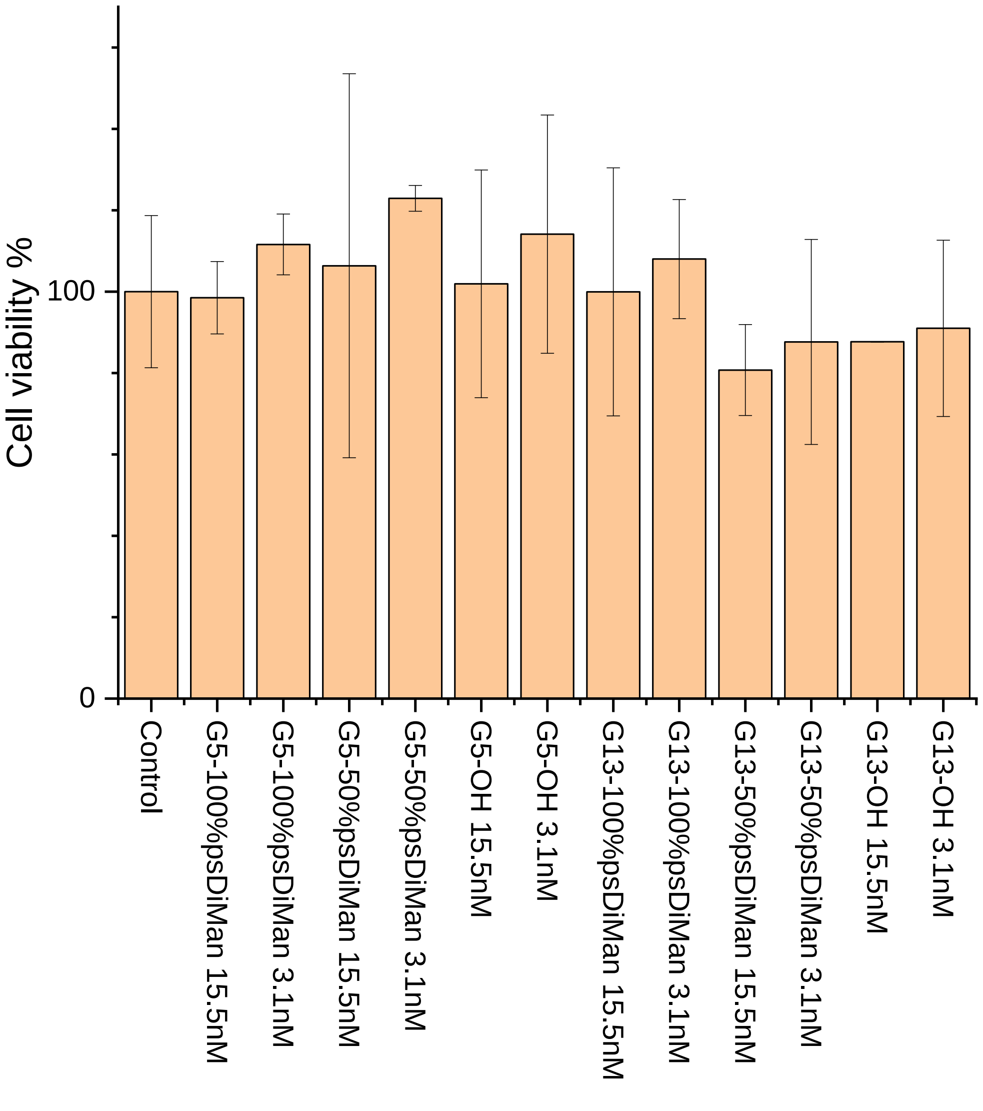

Figure S20. Normalised HEK 293 cell viabilities after treatment with G5-psDiMan, G5-OH, G13-psDiMan and G13-OH against the PBS control assessed by using MTT assay. No significant cell viability differences are observed, indicating no measurable cytotoxicity of the Gx-glycan conjugates.

8. Virus Inhibition[1, 8]

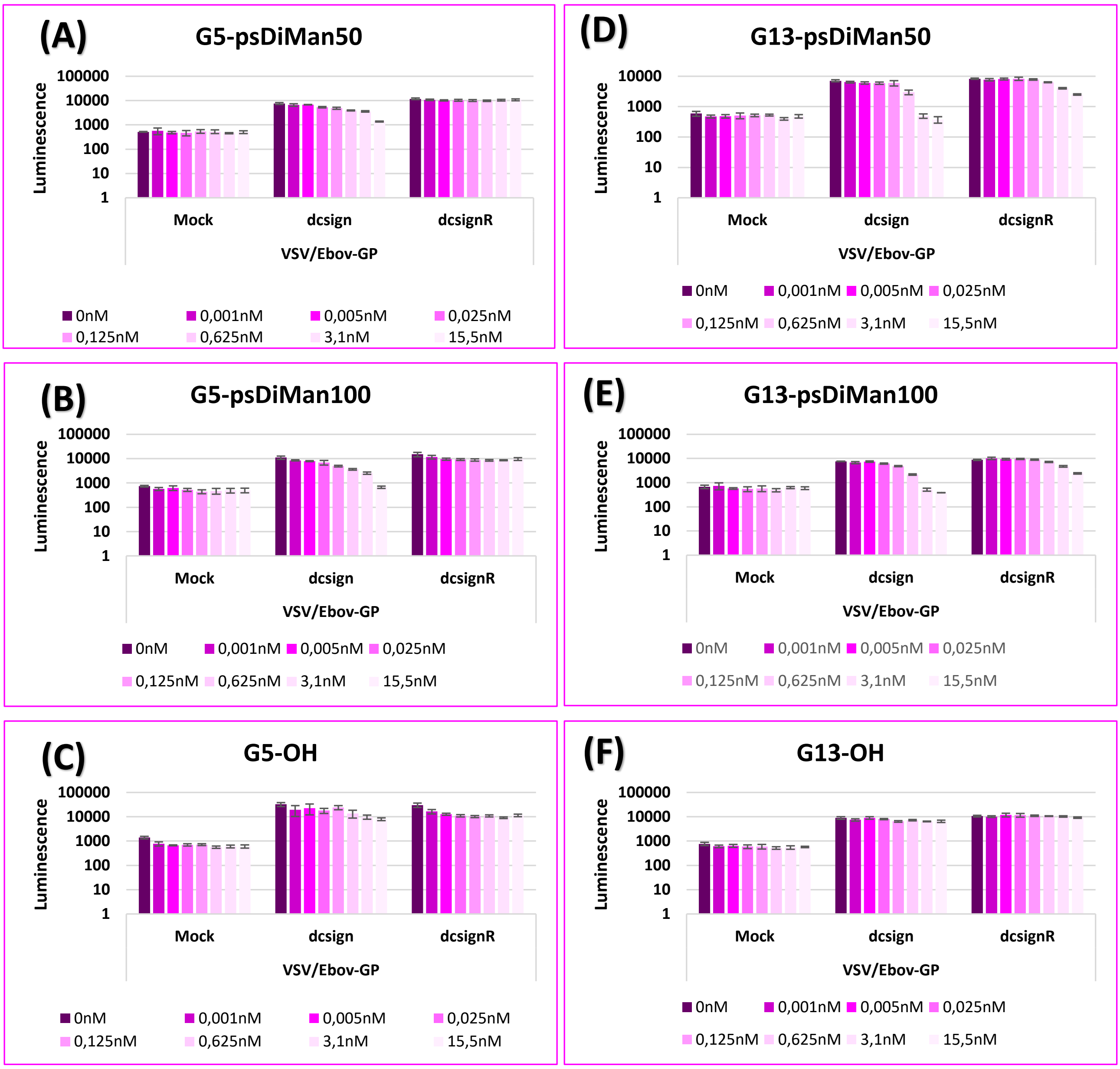

Figure S21. Unprocessed cellular luciferase activities of 293T cells transfected to express DC-SIGN or DC-SIGNR pre-incubated with G5-psDiMan50 (**A**), G5-psDiMan100 (**B**), G5-OH control (**C**), G13-psDiMan50 (**D**), G13-psDiMan100 (**E**) or G13-OH control (**F**) at escalating concentrations and finally inoculated with VSV reporter particles bearing the EBOV-GP. The concentrations of GNPs shown were the final concentrations after addition of particles. The results of a single experiment performed with technical quadruplicates are shown. Error bars indicate standard deviations.

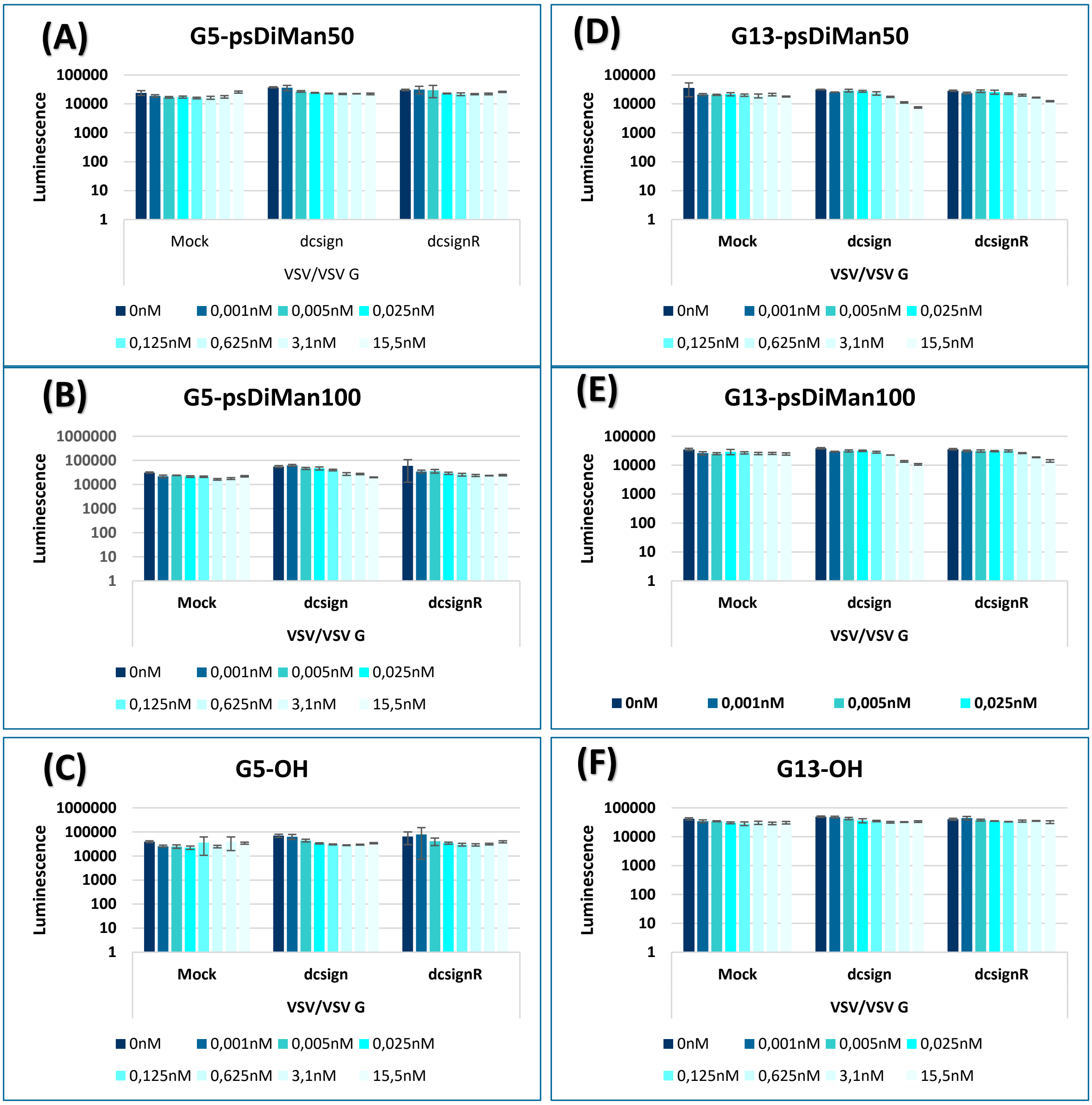

Figure S22. Unprocessed cellular luciferase activities of 293T cells transfected to express DC-SIGN or DC-SIGNR after pre-incubation with G5-psDiMan50 (**A**), G5-psDiMan100 (**B**), G5-OH control (**C**), G13-psDiMan50 (**D**), G13-psDiMan100 (**E**) or G13-OH control (**F**) at escalating concentrations followed by inoculation with VSV reporter particles bearing the VSV-G protein. The GNP concentrations were attained after addition of particles. The results of a single experiment performed with technical quadruplicates are shown. Error bars indicate standard deviations.

9. References

(1) Budhadev, D.; Poole, E.; Nehlmeier, I.; Liu, Y.; Hooper, J.; Kalverda, E.; Akshath, U. S.; Hondow, N.; Turnbull, W. B.; Pohlmann, S.; et al. Glycan-Gold Nanoparticles as Multifunctional Probes for Multivalent Lectin-Carbohydrate Binding: Implications for Blocking Virus Infection and Nanoparticle Assembly. *J. Am. Chem. Soc.* **2020**, *142* (42), 18022-18034.

(2) Budhadev, D.; Hooper, J.; Rocha, C.; Nehlmeier, I.; Kempf, A. M.; Hoffmann, M.; Krüger, N.; Zhou, D. J.; Pöhlmann, S.; Guo, Y. Polyvalent Nano-Lectin Potently Neutralizes SARS-CoV-2 by Targeting Glycans on the Viral Spike Protein. *JACS Au* **2023**, *3* (6), 1755-1766.

(3) Reina, J. J.; Sattin, S.; Invernizzi, D.; Mari, S.; Martínez-Prats, L.; Tabarani, G.; Fieschi, F.; Delgado, R.; Nieto, P. M.; Rojo, J.; et al. 1,2-mannobioside mimic:: Synthesis, DC-SIGN interaction by NMR and docking, and antiviral activity. *ChemMedChem* **2007**, *2* (7), 1030-1036.

(4) Song, L.; Ho, V. H. B.; Chen, C.; Yang, Z. Q.; Liu, D. S.; Chen, R. J.; Zhou, D. J. Efficient, pH-Triggered Drug Delivery Using a pH-Responsive DNA-Conjugated Gold Nanoparticle. *Adv. Healthc. Mater.* **2013**, *2* (2), 275-280. Song, L.; Guo, Y.; Roebuck, D.; Chen, C.; Yang, M.; Yang, Z. Q.; Sreedharan, S.; Glover, C.; Thomas, J. A.; Liu, D. S.; et al. Terminal PEGylated DNA-Gold Nanoparticle Conjugates Offering High Resistance to Nuclease Degradation and Efficient Intracellular Delivery of DNA Binding Agents. *ACS Appl. Mater. Interfaces* **2015**, *7* (33), 18707-18716.

(5) Ulman, A. Formation and structure of self-assembled monolayers. *Chem. Rev.* **1996**, *96* (4), 1533-1554.

(6) Guo, Y.; Sakonsinsiri, C.; Nehlmeier, I.; Fascione, M. A.; Zhang, H.; Wang, W.; Pöhlmann, S.; Turnbull, W. B.; Zhou, D. Compact, polyvalent mannose quantum dots as sensitive, ratiometric FRET probes for multivalent protein–ligand interactions. *Angew. Chem. Int. Ed.* **2016**, *55* (15), 4738-4742.

(7) Hill, H. D.; Millstone, J. E.; Banholzer, M. J.; Mirkin, C. A. The Role Radius of Curvature Plays in Thiolated Oligonucleotide Loading on Gold Nanoparticles. *ACS Nano* **2009**, *3* (2), 418-424.

(8) Guo, Y.; Nehlmeier, I.; Poole, E.; Sakonsinsiri, C.; Hondow, N.; Brown, A.; Li, Q.; Li, S.; Whitworth, J.; Li, Z.; et al. Dissecting Multivalent Lectin-Carbohydrate Recognition Using Polyvalent Multifunctional Glycan-Quantum Dots. *J. Am. Chem. Soc.* **2017**, *139* (34), 11833-11844.

(9) Turnbull, W. B.; Daranas, A. H. On the value of c: can low affinity systems be studied by isothermal titration calorimetry? *J. Am. Chem. Soc.* **2003**, *125* (48), 14859-14866.
